# Roles of Pyroptosis-Related Genes in the Diagnosis and Subtype Classification of Periodontitis

**DOI:** 10.1155/2023/8757233

**Published:** 2023-04-10

**Authors:** Xiaofan Cheng, Yifang Hu, Guan Gui, Xiaoya Hu, Jie Zhu, Bowei Shi, Shoushan Bu

**Affiliations:** ^1^Department of Stomatology, The First Affiliated Hospital of Nanjing Medical University, Nanjing, China; ^2^Department of Geriatric Endocrinology, The First Affiliated Hospital of Nanjing Medical University, Nanjing, China

## Abstract

Pyroptosis is widely involved in many diseases, including periodontitis. Nonetheless, the functions of pyroptosis-related genes (PRGs) in periodontitis are still not fully elucidated. Therefore, we aimed to investigate the role of PRGs in periodontitis. Three datasets (GSE10334, GSE16134, and GSE173078) from the Gene Expression Omnibus (GEO) were selected to analyze the differences in expression values of the PRGs between nonperiodontitis and periodontitis tissue samples using difference analysis. Following this, five hub PRGs (charged multivesicular body protein 2B, granzyme B, Z-DNA-binding protein 1, interleukin-1*β*, and interferon regulatory factor 1) predicting periodontitis susceptibility were screened by establishing a random forest model, and a predictive nomogram model was constructed on the basis of these genes. Decision curve analysis suggested that the PRG-based predictive nomogram model could provide clinical benefits to patients. Three distinct PRG patterns (cluster A, cluster B, and cluster C) in the periodontitis samples were revealed according to the 48 significant PRGs, and the difference in the immune cell infiltration among the three patterns was explored. We observed that all infiltrating immune cells, except type 2 T helper cells, differ significantly among the three patterns. To quantify the PRG patterns, the PRG score was calculated by principal component analysis. According to the results, cluster B had the highest PRG score, followed by cluster A and cluster C. In conclusion, PRGs significantly contribute to the development of periodontitis. Our study of PRG patterns might open up a new avenue to guide individualized treatment plans for patients with periodontitis.

## 1. Introduction

Periodontitis is a chronic multifactorial inflammatory disease linked to the dental plaque accumulation, host immune response, and environmental and systemic factors. The disease occurs in periodontal supporting tissue and causes irreversible damage, including gingival recession, clinical attachment loss, and alveolar bone resorption, ultimately resulting in the loss of teeth [1]. Periodontal infection is one of the most prevalent oral diseases, with approximately 11% of the global population suffering from severe periodontitis, not only posing a considerable threat to oral and general human health, but also placing massive burdens on the healthcare system and social economy [2]. The complex dynamic interaction between host immune defense mechanisms and the microbes in dental plaque biofilms contributes to periodontal inflammation. Although bacteria are crucial in periodontal inflammation initiation, the host's immune response determines the disease progression and severity [3–5]. Therefore, figuring out the regulating mechanisms of immunological and inflammatory responses is critical for revealing the pathological mechanisms of periodontitis.

Pyroptosis is predominantly mediated by the Gasdermin family. It is mainly manifested by the formation of pores in the cell membrane, the rapid expansion of each cell, and eventually cell lysis. When cells rupture, large amounts of intracellular contents are released, including danger-associated molecular patterns and cytokines, and an intense inflammatory response is induced. Based on the different signaling pathways, pyroptosis can be divided into the canonical pyroptotic death that depends on caspase-1 and the noncanonical pyroptotic death that depends on human caspase-4, 5 and murine orthologs caspase-11 [6–8]. Pyroptosis is critical to the development and progression of infectious diseases, neurological diseases, atherosclerotic diseases, and tumors [7, 9–12]. Reportedly, an association exists between pyroptosis and periodontitis [13–15]. Jun et al. [16] reported that Td92, *Treponema denticola*'s surface protein, triggers caspase-4 activation and pyroptosis in human gingival fibroblasts. Moreover, NLRP6 induced pyroptosis of human gingival fibroblasts by activating caspase-1 and promoted the production of IL-1*β*, the level of which may reflect the severity of periodontitis [17]. In-depth research on pyroptosis will help provide novel targets and ideas for clinical prevention and treatment of periodontitis.

**Figure 1:**
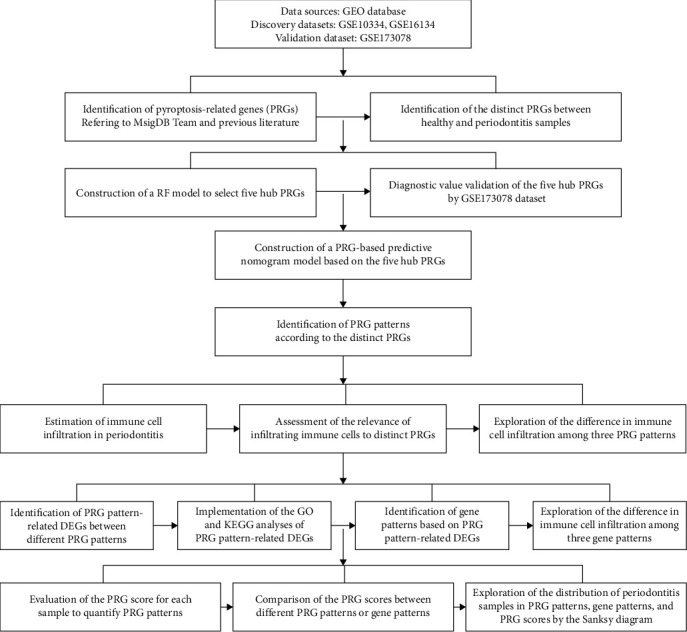
The workflow of analysis process. GEO, Gene Expression Omnibus; PRG, pyroptosis-related gene; RF, random forest; DEG, differentially expressed gene.

Recently, with the advances in sequencing technology and microarray, bioinformatics has been widely used to identify the promising biomarkers for disease diagnosis and prognosis, and to explore the pathogenesis at the genetic level, thus providing new targets for intervention and new treatments for diseases [18, 19]. This study systematically analyzed the functions of pyroptosis-related genes (PRGs) in the diagnosis and subtype classification of periodontitis. We developed a PRG-based predictive model for predicting the probability of periodontitis on the basis of five hub PRGs (charged multivesicular body protein 2B (CHMP2B), granzyme B (GZMB), Z-DNA-binding protein 1 (ZBP1), interleukin (IL)-1*β*, and interferon regulatory factor 1 (IRF1)) and found that patients could benefit from the model. Our study is the first to construct a PRG-based predictive nomogram model to predict the risk of periodontitis. Furthermore, we identified three distinct PRG patterns that are crucial in regulating periodontitis immune microenvironments, indicating that PRG patterns might help classify periodontitis from a molecular perspective and guide personalized therapeutic methods. The concise workflow of this study is illustrated in Figure 1.

## 2. Materials and Methods

### 2.1. Data Acquisition and Process

The keywords “periodontitis”, “periodontal tissues”, “gene expression”, and “*Homo sapiens*” were used to search the Gene Expression Omnibus (GEO) database for gene expression profiles of the patients. The following criteria were used to screen the obtained datasets. First, both health and disease groups should be included in the profile information. Second, all samples must be from gingival tissues. Third, these datasets are supposed to provide raw data that can be further studied. Eventually, three datasets (GSE10334, GSE16134, and GSE173078) were selected for the next research. GSE10334 contains expression data of 183 periodontitis and 64 healthy samples, GSE16134 includes 241 periodontitis and 69 healthy samples, and GSE173078 is comprised of 12 periodontitis and 12 healthy samples [20–22]. The researchers deployed the GSE10334 and GSE16134 datasets as the discovery datasets for the entire study and used the GSE173078 dataset as the external verification dataset to validate the selected hub PRGs.

### 2.2. Landscape of the PRGs

We referred to the MsigDB Team (GOBP-PYROPTOSIS, REACTOME-PYROPTOSIS) and previous literature, and retrieved 55 PRGs, of which 53 coincided with the genes from GSE10334 to GSE16134 [23]. These genes are listed in Table S1 (see *Supplementary 2*). The differences in PRGs expression values between the healthy and periodontitis samples were analyzed utilizing the “limma” package in R. The expression relationships among 53 PRGs were investigated using linear regression analysis in all and periodontitis samples.

### 2.3. Construction of a Model for Selecting Hub PRGs

We developed two training models to predict the onset of periodontitis, utilizing random forest (RF) and support vector machines (SVM). The models were evaluated using the “Reverse Cumulative Distribution of Residual,” “Residual Boxplots,” and receiver operating characteristic (ROC) curve. The optimal prediction model was selected based on a lower residual value and a higher area under the ROC curve (AUC). RF is an ensemble method based on multiple decision trees. The “RandomForest” package was performed to create an RF model to screen hub PRGs among the 53 PRGs to estimate the probability of periodontitis. In our study, the optimal number of trees was 150, and the number of variables randomly sampled as candidates at each split was set at three. Then, we evaluated and ranked the importance of the 48 significant PRGs and considered the top five PRGs as the hub PRGs. SVM is mainly used for binary classification. Every data in our research were represented by a dot in the *n*-dimensional space (*n* is 53 in our research). A perfect hyperplane in the space was then discovered that could effectively separate the two classes (healthy and periodontitis) [24]. Furthermore, the prediction ability of the selected hub PRGs was validated by GSE173078 dataset.

### 2.4. Construction of a PRG-Based Predictive Model

We constructed a predictive nomogram model with the selected five hub PRGs using the “rms” package in R. Each variable was assigned a corresponding score in the nomogram scoring system, and the total score for each sample was determined by summing the scores of all variables. Then, the prevalence of periodontitis was estimated according to the total score. The calibration curve was conducted to assess the agreement between our predicted values and actual values. To determine whether decisions made using the model could benefit the patient, we conducted a decision curve analysis (DCA) and generated a clinical impact curve [24].

### 2.5. Identification of PRG Patterns

According to the significant PRGs, we utilized the “ConsensusClusterPlus” package in R software to perform consensus clustering analysis in order to categorize periodontitis samples into distinct molecular patterns [25]. The optimal clustering number was mainly decided on the basis of a smooth and progressive increase in the cumulative distribution function (CDF) curve and the absence of any groups with too small sample size.

### 2.6. Estimation of Immune Cell Infiltration

We used single-sample gene-set enrichment analysis (ssGSEA) to determine the levels of specific infiltrating immune cells [26]. The gene sets for infiltrating immune cells were obtained from published literature [27]. The enrichment scores representing immune cell abundance were compared among PRG patterns. We performed a Spearman correlation analysis to assess the correlation between PRGs and immune cell fractions.

### 2.7. Identification of PRG Pattern-Related Differentially Expressed Genes (DEGs) and Functional Annotation

We utilized the “limma” package in R to identify DEGs between different PRG patterns, with a criteria of log|fold change| > 0.2 and adjusted *p*-value < 0.0001. To explore the potential mechanisms of these significant DEGs, we conducted Gene Ontology (GO) and Kyoto Encyclopedia of Genes and Genomes (KEGG) enrichment analyses using the “clusterProfiler” package in R. Enrichment results with an adjusted *p*-value < 0.05 were considered significant [28].

### 2.8. Estimation of PRG Signature

To quantify the PRG patterns, we evaluated the PRG score for each sample via principal component analysis (PCA). We first performed PCA to differentiate the PRG patterns and then determined the PRG score using the following equation: PRG score = PC1_i_, where PC1 represents principal component 1, and i represents DEG expression [27].

## 3. Results

### 3.1. The Landscape of the PRGs between the Healthy and Periodontitis Samples

**Figure 2:**
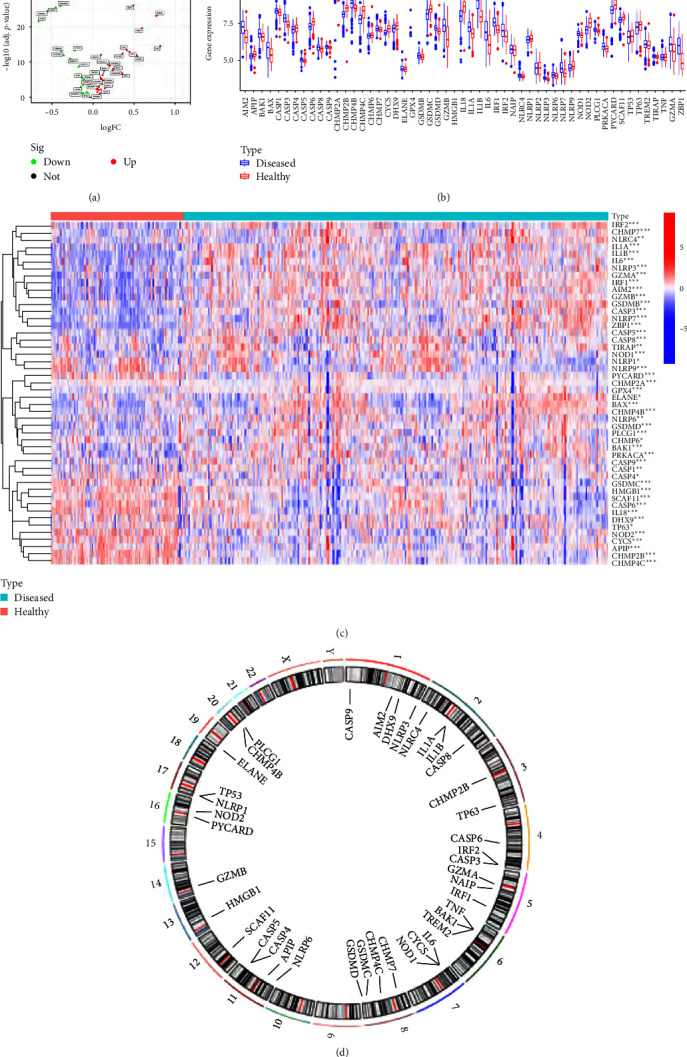
Landscape of the PRGs. (a, b) The volcano map and the histogram showed the expression distributions of 53 PRGs between healthy and periodontitis samples. (c) The heat map presented the expression status of 48 distinct PRGs between healthy and periodontitis samples. (d) Chromosomal locations of the 53 PRGs.  ^*∗*^*p* < 0.05,  ^*∗∗*^*p* < 0.01, and  ^*∗∗∗*^*p* < 0.001. PRG, pyroptosis-related gene.

Gene expression profiles from the whole periodontal tissue samples in 436 periodontitis and 145 healthy controls were obtained from the GSE10334, GSE16134, and GSE173078 datasets. Fifty-three PRGs were involved in our study, of which 48 demonstrated significantly differential expression between the healthy and periodontitis samples. The distinct PRGs were displayed by a volcano map, a heat map, and a histogram. Compared with the healthy samples, we discovered 30 upregulated PRGs and 18 downregulated PRGs in the periodontitis samples (Figure 2(a)–2(c)). Visualization of chromosomal positions in PRGs was achieved using the “RCircos” package (Figure 2(d)).

### 3.2. Correlation Analysis between PRGs in All Samples and Periodontitis Samples

**Figure 3:**
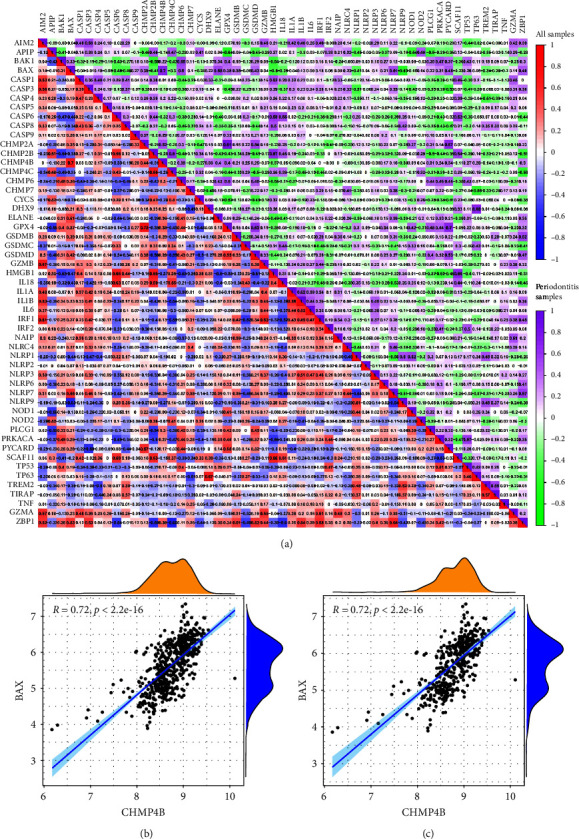
Correlations among 53 PRGs in all samples and periodontitis samples. (a) Correlations among 53 PRGs in all samples and periodontitis samples. (b) CHMP4B and BAX had the highest significant correlation in all samples. (c) CHMP4B and BAX had the highest significant correlation in periodontitis samples. PRG, pyroptosis-related gene.

The “corrplot” package was used to implement the correlation analysis among the PRGs in all samples and periodontitis samples. Results revealed a significant correlation between most PRGs in all samples and periodontitis samples, suggesting that there may be potential synergistic effects between PRGs and that pyroptosis is crucial in periodontitis process (Figure 3(a)). The CHMP4B and BAX had the highest significant correlation in both two cohorts indicating that they might function together (Figures 3(b) and 3(c)).

### 3.3. Construction of the Model for Selecting Hub PRGs

**Figure 4:**
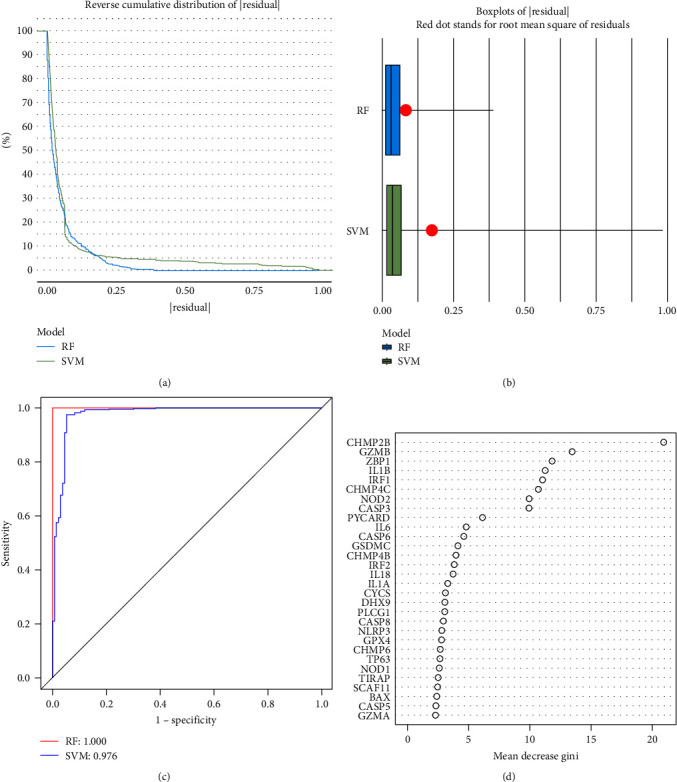
Construction of the RF model. (a, b) The residual distribution of the RF and SVM models. (c) The accuracy of the RF and SVM models was evaluated by the area under the ROC curves. (d) The top 30 PRGs was selected according to the importance ranking. RF, random forest; SVM, support vector machines; ROC, receiver operating characteristic.

RF and SVM models were constructed to screen hub PRGs from the 48 distinct PRGs. In comparison with the SVM model, the RF model has smaller residuals and is better at predicting periodontitis susceptibility, as presented by the reverse cumulative distribution of residual curve (Figure 4(a)) and the residual boxplots (Figure 4(b)). We depicted the ROC curve to further evaluate both models and the higher AUC value of the RF model proved its superiority (Figure 4(c)). Thus, the optimal model for predicting the occurrence of periodontitis was the RF model. We showed the top 30 genes after ranking the significant 48 PRGs genes on the grounds of their importance (Figure 4(d)). The top five genes (CHMP2B, GZMB, ZBP1, IL1*β*, and IRF1) in the importance ranking were deemed as the hub genes. Furthermore, the external validation conducted on independent dataset GSE173078 also showed that the selected five hub genes have good prediction ability (*Supplementary 1*).

### 3.4. Construction of the PRG-Based Predictive Model

**Figure 5:**
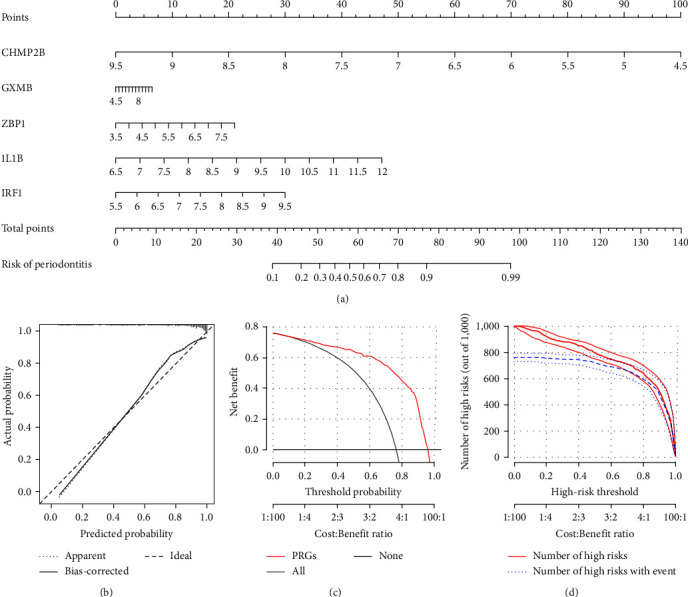
Building of the PRG-based predictive model. (a) The predictive model was constructed on the basis of the five hub PRGs. (b) The calibration curve demonstrated the predictive ability of the model. (c) Patients of periodontitis may derive benefits from the model. (d) The clinical impact curve assessed the clinical impact of the model. PRG, pyroptosis-related gene.

Based on the five hub PRGs, a predictive nomogram model was created to predict the probability of periodontitis (Figure 5(a)). The calibration curve demonstrated the accurate predictive ability of the PRG-based predictive model (Figure 5(b)). The DCA curve suggested that decisions on the basis of the PRG-based predictive model could be advantageous to patients with periodontitis (Figure 5(c)). In addition, the clinical impact curve demonstrated that the nomogram model had prominent predictive power (Figure 5(d)).

### 3.5. Three Distinct PRG Patterns Identified by PRGs

**Figure 6:**
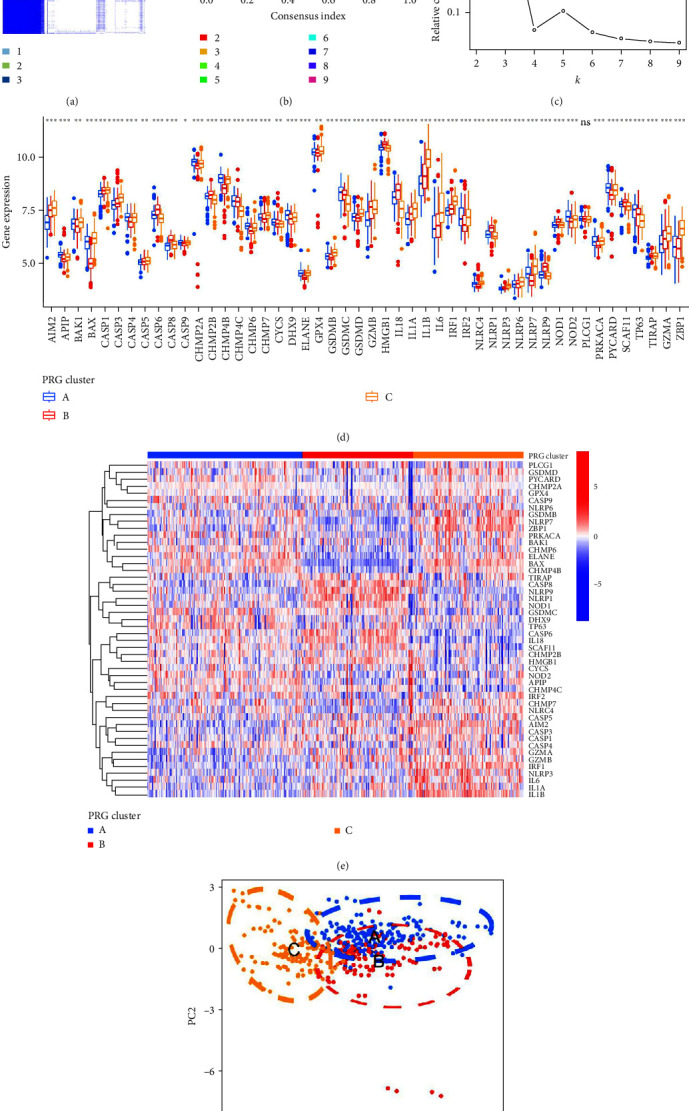
Consensus clustering of the 48 distinct PRGs in periodontitis. (a) Consensus matrices of the 48 distinct PRGs for *k* = 3. (b) CDF for *k* = 2–9. (c) Relative change in area under CDF curve for *k* = 2–9. (d) Differential expression levels of the 48 distinct PRGs in three PRG patterns. (e) Expression heat map of the 48 significant PRGs in three PRG patterns. (f) PCA for the expression profiles of the three PRG patterns.  ^*∗*^*p* < 0.05,  ^*∗∗*^*p* < 0.01, and  ^*∗∗∗*^*p* < 0.001. PRG, pyroptosis-related gene; CDF, cumulative distribution function; PCA, principal component analysis.

Unsupervised clustering analysis for periodontitis samples was conducted according to the expression of 48 distinct PRGs to investigate PRG patterns in periodontitis. We identified three different PRG patterns (cluster A, cluster B, and cluster C) (Figure 6(a)–6(c)). Cluster A contained 175 samples, cluster B involved 125 samples, and cluster C included 124 samples. The histogram and heat map were generated to present the significant differences in expression values of the 48 distinct PRGs among the three clusters (Figures 6(d) and 6(e)), and PCA was performed to further verify the role of significant PRGs in differentiating distinct PRG patterns. With the exception of PLCG1, all 48 significant PRGs were significantly differentially expressed among the three PRG patterns (Figure 6(d)), confirming the diversity of PRG patterns in periodontitis. PCA for the transcriptome profiles of three PRG patterns revealed that the 48 significant PRGs could well distinguish the three different PRG patterns (Figure 6(f)).

### 3.6. Estimation of Immune Cell Infiltration

**Figure 7:**
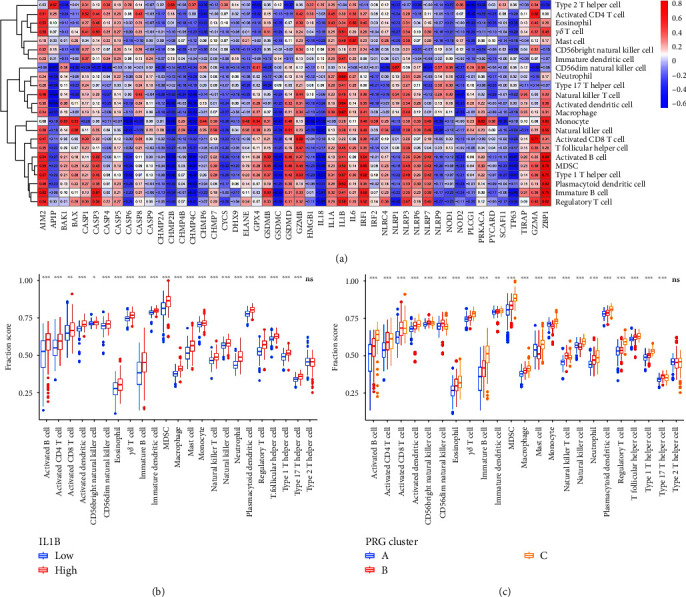
Estimation of immune cell infiltration. (a) Relevance of infiltrating immune cells to 48 different PRGs; (b) Differential immune cells infiltration between high and low IL-1*β* level groups. (c) The abundance differences of immune cell infiltration among three PRG patterns.  ^*∗*^*p* < 0.05,  ^*∗∗*^*p* < 0.01, and  ^*∗∗∗*^*p* < 0.001. PRG, pyroptosis-related gene.

The infiltrating immune cell abundance in periodontitis was evaluated using ssGSEA, and the relevance of infiltrating immune cells to 48 different PRGs were assessed. Our study identified that PRGs are closely related to different infiltrating immune cells and IL-1*β* positively correlates with a variety of immune cells (Figure 7(a)). We examined the differences in immune cell infiltration between patients with high and low IL-1*β* levels. The results indicated that patients with high IL-1*β* levels had greater immune cell infiltration compared with those with low IL-1*β* levels (Figure 7(b)). Lastly, the difference in immune cell infiltration among the three PRG patterns was explored. We found that all immune cells, except type 2 T helper cells, differ significantly among the three patterns. Compared with cluster B and cluster C, cluster A exhibited relatively low immune cell infiltration. Cluster B was enriched in CD56dim natural killer cells, natural killer T cells, and type-17 T helper cells, while cluster C had higher levels of infiltrated activated B cells, activated CD4 T cells, activated dendritic cells, eosinophils, gamma delta T cells, immature B cells, MDSCs, macrophages, mast cells, monocytes, natural killer cells, neutrophils, plasmacytoid dendritic cells, regulatory T cells, T follicular helper cells, and type-1 T helper cells (Figure 7(c)). These findings suggested cluster A mediates a modest immune reaction in periodontitis while cluster B and cluster C generate an active immune reaction, and the immune reaction caused by cluster B and cluster C is different. The above findings once again proved that PRGs are essential for regulating the immune microenvironments in periodontitis.

### 3.7. Identification of Gene Patterns Based on PRG Pattern-Related DEGs

**Figure 8:**
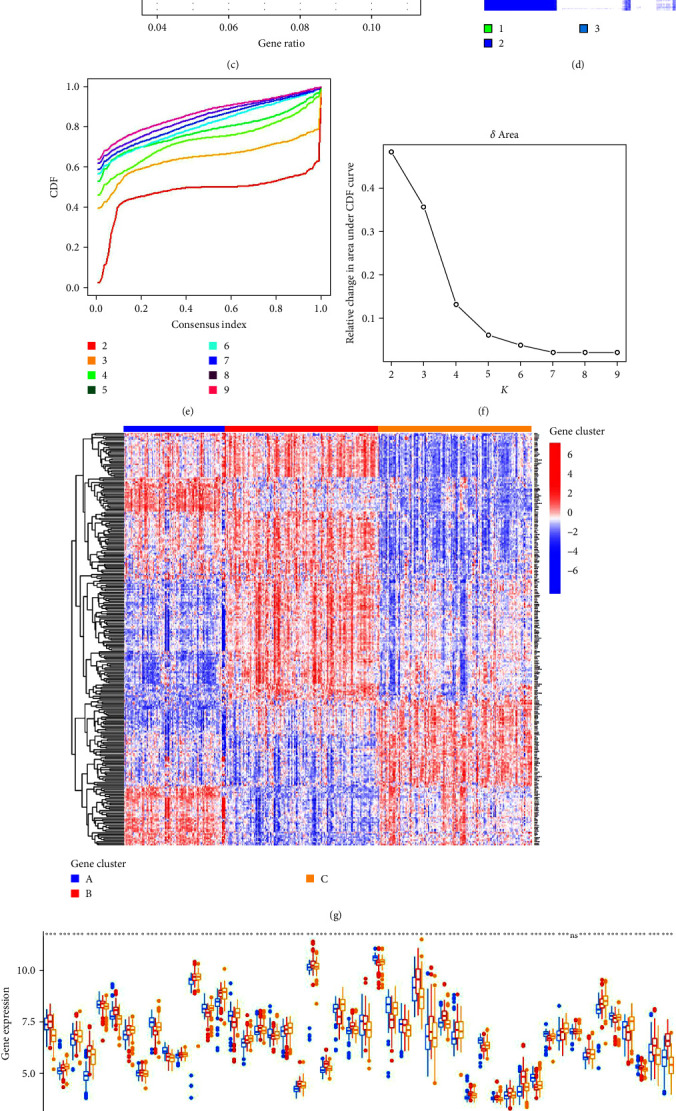
Consensus clustering of the 278 PRG pattern-related DEGs in periodontitis. (a) The 278 PRG pattern-related DEGs shown in Venn diagram. (b, c) GO and KEGG analyses that explored possible mechanisms of the 278 PRG pattern-related DEGs in periodontitis. (d) Consensus matrices of the 278 PRG pattern-related DEGs for *k* = 3. (e) Consensus clustering CDF for *k* = 2–9. (f) Relative change in area under CDF curve for *k* = 2–9. (g) Expression heat map of the 278 PRG pattern-related DEGs in three gene patterns. (h) Difference in expression of the 278 PRG pattern-related DEGs in three gene patterns. (i) Difference in immune cell infiltration between distinct gene patterns.  ^*∗*^*p* < 0.05,  ^*∗∗*^*p* < 0.01, and  ^*∗∗∗*^*p* < 0.001. PRG, pyroptosis-related gene; DEG, differentially expressed gene; GO, gene ontology; KEGG, Kyoto Encyclopedia of Genes and Genomes; CDF, cumulative distribution function.

**Figure 9:**
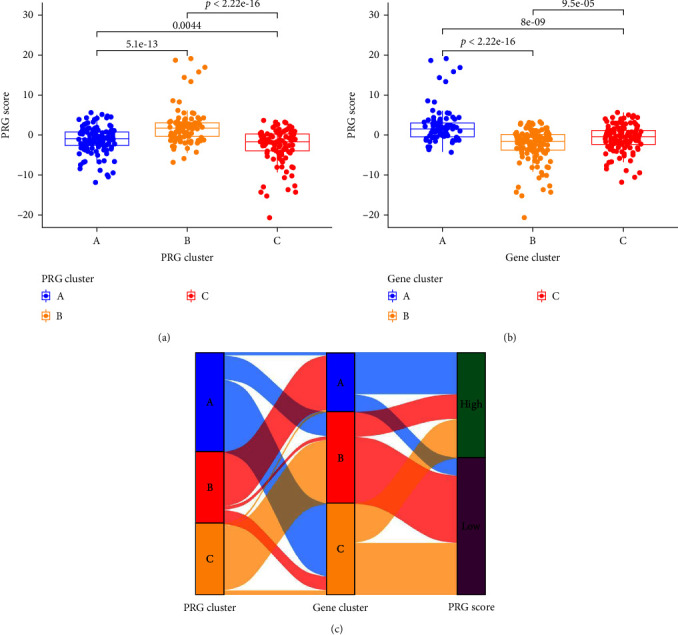
The relationship between PRG patterns, PRG gene patterns, and PRG scores. (a) Differential PRG score between distinct PRG patterns. (b) Differential PRG score between distinct gene patterns. (c) Sankey diagram showing the distribution of periodontitis samples in PRG patterns, gene patterns, and PRG scores. PRG, pyroptosis-related gene.

A total of 278 PRG pattern-related DEGs were singled out between the three PRG patterns, which underwent both GO and KEGG enrichment analyses to explore the potential biological behavior of each PRG pattern (Figure 8(a)–8(c)). The results demonstrated that the DEGs were mostly enriched in biological processes including GO:0070661, GO:0046651, GO:0032943, GO:1903131, G0:0002460, and GO:0030098, all of which are closely associated with immune cell differentiation, activation, and proliferation (Figure 8(b)). The KEGG analysis showed enrichment of immune pathways, including viral protein interaction with cytokines and cytokine receptors, primary immunodeficiency, etc. (Figure 8(c)). To further verify the PRG patterns, periodontitis samples were divided into three gene patterns (gene clusters A, B, and C) on the basis of the 278 PRG pattern-related DEGs using consensus clustering algorithm, which was in line with the PRG patterns (Figure 8(d)–8(f)). The expression levels of the 48 significant PRGs in different gene patterns are shown in Figures 8(g) and 8(h). All 48 significant PRGs, except PLCG1, were significantly differentially expressed in the three gene patterns, which was consistent with the result of differential expression analysis of 48 significant PRGs in the three PRG patterns. However, the abundance difference in immune cell infiltration among gene patterns differs subtly from those among PRG patterns (Figure 8(i)). We applied PCA to evaluate the PRG score for each sample to quantify PRG patterns and then compared the PRG score between the different PRG patterns or gene patterns (Figures 9(a)) and 9(b)). The findings indicated cluster A or gene cluster A got a higher PRG score than cluster C or gene cluster C, but cluster B got the highest score in PRG patterns while gene cluster B got the lowest score in gene patterns. The Sankey diagram showed the distribution of periodontitis samples in PRG patterns, gene patterns, and PRG scores and indicated directly the relationship between PRG patterns, gene patterns, and PRG scores (Figure 9(c)).

## 4. Discussion

Periodontitis is an immune inflammatory disease initiated by microorganisms in the dental plaque and causes the irreversible destruction of bone and connective tissue. As the disease progresses, the teeth loosen, become dysfunctional, and eventually fall out [1]. Increasing evidence has shown that pyroptosis contributes to various diseases, including infectious disease, nervous system-related diseases, atherosclerotic diseases, and tumors [9–12]. However, the functions of PRGs in periodontitis are still not fully elucidated. Our study aimed to investigate the possible mechanisms of PRGs in periodontitis.

Using the difference analysis, we found that 48 distinct PRGs among 53 PRGs differed significantly in expression between healthy and periodontitis samples, indicating that PRGs are involved in the pathogenesis of periodontitis. The machine-learning method, RF, was performed to create an ideal model to predict the occurrence of periodontitis and selected five hub PRGs (CHMP2B, GZMB, ZBP1, IL1B, and IRF1). This finding differs from that of Chen et al. [29], who conducted least absolute shrinkage and selection operator (LASSO) regression and logistic regression analysis to identify the four hub PRGs (cytochrome c, somatic (CYCS), caspase 3 (CASP3), nucleotide-binding oligomerization domain 2 (NOD2), and charged multivesicular body protein 4b (CHMP4B)). Differences in the discovery datasets and research methods may be responsible for the discrepancy between the results. Based on the five hub PRGs, we then developed a PRG-based predictive model to estimate the probability of periodontitis and decisions on the basis of the predictive model may be advantageous to patients with periodontitis, as presented by the DCA curve. Our study is the first to establish a PRG-based predictive nomogram model to predict the risk of periodontitis from a molecular perspective. CHMP2B, a nuclear member of the endosomal sorting required for transport complex III, is integral to endolysosomal trafficking, vesicle fusion, and autophagic degradation [30]. It resides in chromosome 3p11-12 region near VGLL3 gene, which shows amplification in diverse sarcomas [31]. GZMB, a serine protease found in the cytoplasmic granules of natural killer cells and cytotoxic T lymphocytes, is a key regulator of skin damage, inflammation, and repair. The level of GZMB is low in healthy skin but is significantly increased in inflammatory and chronic skin diseases, such as cutaneous leishmaniasis, diabetic ulcers, hypertrophic scarring, and autoimmune skin disorders. GZMB is essential for many physiological activities in cells, including proapoptotic activity, cleavage of extracellular matrix proteins, disruption of epithelial barrier, fibrosis, vascular permeability, anoikis, inflammation, and autoimmunity [32, 33]. ZBP1 is an essential innate immune sensor of endogenous nucleic acid ligands and viral RNA products. ZBP1 sensing of virus infection can induce pyroptosis, apoptosis, and necroptosis (PANoptosis). It is reported that adenosine deaminase acting on RNA1 (ADAR1) suppresses ZBP1-mediated PANoptosis, promoting tumorigenesis [34, 35]. IL-1*β*, a proinflammatory cytokine, is produced primarily by blood monocytes, tissue macrophages, skin dendritic cells, and brain microglia [36]. IL-1*β* in primary tumors is reportedly a promising biomarker for predicting the increased risk of bone metastasis in breast cancer patients [37]. Besides, there exists clinical evidence on the correlation of IL-1*β* with periodontitis. Increased IL-1*β* triggers inflammation and promotes bone resorption in periodontitis [38]. IRF1, the first identified IRF, has been demonstrated to be implicated in varieties of physical and pathological processes, including viral infection, tumor immunosurveillance, proinflammatory injury, and immune diseases [39, 40]. IRF1 loss combined with other genetic alterations prominently increases tumor incidence of many organs in mice [41]. Emerging evidence has shown that the five hub PRGs extensively participate in the development and progression of tumors, including proliferation, invasion, radiotherapy resistance, and prognosis [31, 35, 37, 42]. However, the roles of the five hub PRGs in the pathogenesis of periodontitis are rarely reported and our study may provide new directions for future researches on these genes.

In our research, three PRG subtypes (clusters A, B, and C) were discovered according to the 48 significant PRGs using the unsupervised clustering analysis, and each subtype has its specific immune properties. Cluster A has relatively low immune cell infiltration compared with cluster B and cluster C, suggesting that cluster A generates a mild immune reaction in periodontitis, and cluster B and cluster C mediate more active immune reactions, while the immune reaction mediated by cluster B and cluster C is different. GO and KEGG enrichment analyses on the 278 PRG pattern-related DEGs showed that the genes were maximally and significantly enriched in mononuclear cell differentiation and cytokine–cytokine receptor interaction, indicating that mononuclear cell differentiation and cytokine–cytokine receptor interaction are essential for pyroptosis regulation in periodontitis. The periodontitis samples were successfully distinguished into three gene patterns on the basis of the 278 PRG pattern-related DEGs, and we found that the differential expression levels of the 48 unique PRGs in the gene patterns were comparable to those in the PRG patterns, providing additional evidence for the accuracy of the PRG subtyping results. Lastly, we utilized PCA to measure the PRG score for each sample and quantify the PRG patterns. Our analysis revealed that the PRG score in cluster A or gene cluster A was higher than that in cluster C or gene cluster C. However, the PRG score in cluster B was highest in the PRG patterns while the PRG score in gene cluster B was lowest in the gene patterns, which might be attributed to the inconsistent distribution of periodontitis samples in the PRG patterns and gene patterns. The specific immune properties of each pattern validated the accuracy of our classification of a molecular perspective. This classification method is widely implemented in the field of oncology and contributed significantly to tumor classification and the accurate prediction of immunotherapy outcome. Song et al. [43] performed this method to identify two distinct molecular subtypes and develop a prognosis model in colorectal cancer, discovering the vital roles of PRGs in tumor immune microenvironment, clinicopathological features, and prognosis. As for the field of periodontitis, Chen et al. [29] also applied this classification strategy to classify periodontitis samples into three distinct patterns with different immune characteristics based on the significant PRGs, which is similar to our results. This subtyping strategy can help us elucidate the possible mechanisms of PRGs implicated in regulating the immune microenvirionment so that precise and personalized treatments can be applied.

However, there are still some limitations to this study. First, the database lacked some important variables such as the clinical characteristics of the samples, microbial information and serum detection. Therefore, in our investigation, analyzing the functions of the PRGs in periodontitis from multiple perspectives is incredibly tough and the results may be biased. Second, the results were obtained by bioinformatics analysis and still demand experimental verification in vivo and in vitro. In addition, the clinical features of the three distinct PRG subtypes of periodontitis should be elaborated. Unfortunately, owing to the limitations of clinical data in the database, we are currently unable to explore their clinical characteristics. Finally, further experiments are necessary to understand the specific mechanisms of the PRGs in the pathogenesis of periodontitis.

## 5. Conclusion

This study uncovered the close relationship between PRGs and periodontitis and further identified the association between PRGs and immune response. The diversity of PRG patterns is crucial in regulating the complex immune microenvironment of periodontitis. These findings shed some light on understanding the pathogenesis of periodontitis and open up a new avenue to guide individualized immunotherapy strategies for patients with periodontitis.

## Data Availability

The datasets analyzed in this study are available in GEO database (https://www.ncbi.nlm.nih.gov/geo/).

## Supplementary Materials

Supplementary 1 The prediction ability of the five hub PRGs validated by GSE173078 dataset. ROC curves indicate the good prediction ability of the five hub PRGs. PRG, pyroptosis-related gene; ROC, receiver operating characteristic.

Supplementary 2 Table S1: PRGs from database and published literature. Table S2: Expression diversity of PRGs. Table S3: Correlations between PRGs in all samples. Table S4: Correlations between PRGs in periodontitis samples. Table S5: The three distinct PRG patterns based on 48 significant PRGs. Table S6: PRG pattern-related DEGs. Table S7: GO enrichment results based on 278 PRG pattern-related DEGs. Table S8: KEGG enrichment results based on 278 PRG pattern-related DEGs. Table S9: The three distinct gene patterns based on 278 PRG pattern-related DEGs.
