# Supplementary figures and images for: Roles of Pyroptosis-Related Genes in the Diagnosis and Subtype Classification of Periodontitis
Source: J Immunol Res. 2023 Apr 10;2023:8757233. doi: 10.1155/2023/8757233 (PMC10114156; doi:10.1155/2023/8757233)

**CHMP2B**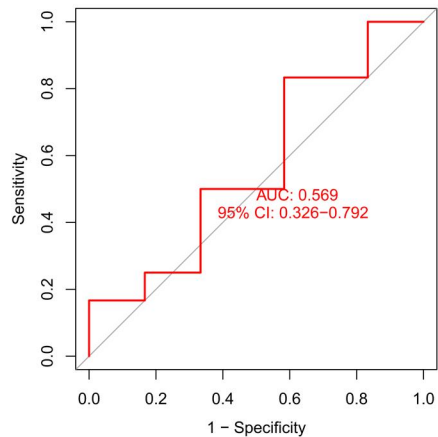**GZMB**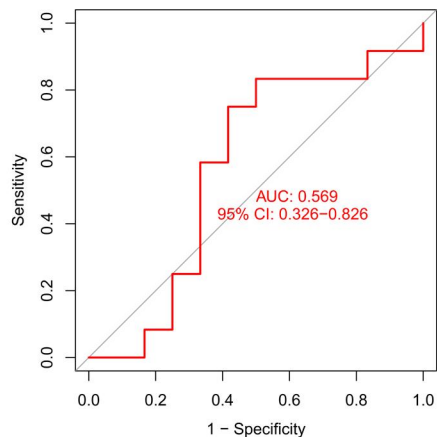**ZBP1**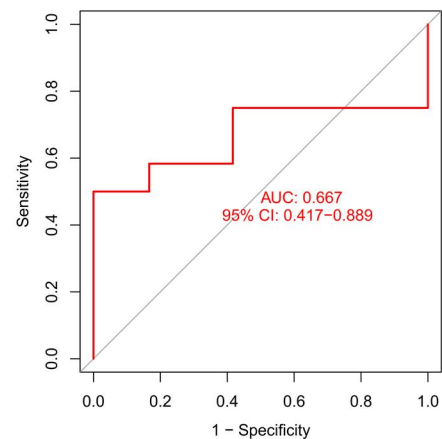**IL1B**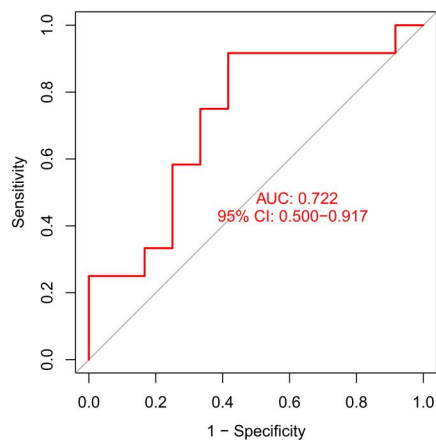**IRF1**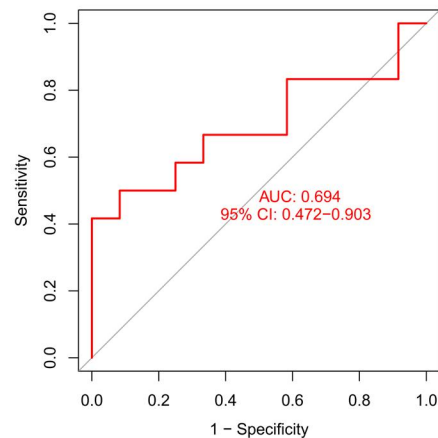

Supplement: Supplementary 1 — The prediction ability of the five hub PRGs validated by GSE173078 dataset. ROC curves indicate the good prediction ability of the five hub PRGs. PRG, pyroptosis-related gene; ROC, receiver operating characteristic. [file 8757233.f1.pdf]
