# Supplementary material for: Roles of Pyroptosis-Related Genes in the Diagnosis and Subtype Classification of Periodontitis
Source: J Immunol Res. 2023 Apr 10;2023:8757233. doi: 10.1155/2023/8757233 (PMC10114156; doi:10.1155/2023/8757233)
Supplement: Supplementary 2 — Table S1: PRGs from database and published literature. Table S2: Expression diversity of PRGs. Table S3: Correlations between PRGs in all samples. Table S4: Correlations between PRGs in periodontitis samples. Table S5: The three distinct PRG patterns based on 48 significant PRGs. Table S6: PRG pattern-related DEGs. Table S7: GO enrichment results based on 278 PRG pattern-related DEGs. Table S8: KEGG enrichment results based on 278 PRG pattern-related DEGs. Table S9: The three distinct gene patterns based on 278 PRG pattern-related DEGs. [file 8757233.f2.pdf]

**Table S1. Pyroptosis-related genes from database and published literature**

AIM2
APIP
BAK1
BAX
CASP1
CASP3
CASP4
CASP5
CASP6
CASP8
CASP9
CHMP2A
CHMP2B
CHMP4A
CHMP4B
CHMP4C
CHMP3
CHMP6
CHMP7
CYCS
DHX9
ELANE
GPX4
GSDMB
GSDMC

GSDMD
GZMB
HMGB1
IL18
IL1A
IL1B
IL6
IRF1
IRF2
NAIP
NLRC4
NLRP1
NLRP2
NLRP3
NLRP6
NLRP7
NLRP9
NOD1
NOD2
PLCG1
PRKACA
PYCARD
SCAF11
TP53
TP63
TREM2

TIRAP
TNF
GZMA
ZBP1

**Table S2. Expression diversity of pyroptosis-related genes**

| ID | logFC | AveExpr | t | P.Value | adj.P.Val | B |
|---|---|---|---|---|---|---|
| IL1B | 1.093264333 | 8.884718375 | 13.59613225 | 1.43E-36 | 6.57E-35 | 72.15442255 |
| ZBP1 | 0.998155383 | 5.735227327 | 13.54246306 | 2.48E-36 | 6.57E-35 | 71.60761663 |
| CASP3 | 0.451563593 | 7.760657355 | 12.09916811 | 4.33E-30 | 7.65E-29 | 57.33993773 |
| IRF1 | 0.489458202 | 7.514940438 | 11.60576418 | 4.78E-28 | 6.34E-27 | 52.67405983 |
| CHMP2B | -0.394698753 | 8.186940135 | -11.50805466 | 1.20E-27 | 1.27E-26 | 51.7640155 |
| NOD2 | -0.448732071 | 7.16482093 | -11.41547642 | 2.85E-27 | 2.51E-26 | 50.90613698 |
| CHMP4C | -0.552444794 | 7.867333826 | -11.1669149 | 2.85E-26 | 2.15E-25 | 48.62426451 |
| GZMB | 0.766788375 | 7.186122648 | 10.77772463 | 9.82E-25 | 6.50E-24 | 45.11593642 |
| IL18 | -0.659236659 | 8.134149469 | -10.35584767 | 4.16E-23 | 2.45E-22 | 41.40564776 |
| AIM2 | 0.594845939 | 7.108461794 | 9.858853565 | 3.03E-21 | 1.60E-20 | 37.16455227 |
| IL6 | 0.815298956 | 6.856079973 | 8.401442813 | 3.67E-16 | 1.77E-15 | 25.60709852 |
| IL1A | 0.411300419 | 7.167036858 | 8.004151105 | 7.01E-15 | 3.10E-14 | 22.70174952 |
| CYCS | -0.225098173 | 6.967687605 | -7.941403303 | 1.11E-14 | 4.51E-14 | 22.25304947 |
| GSDMC | -0.380194144 | 8.18064351 | -7.878958438 | 1.74E-14 | 6.57E-14 | 21.80931006 |
| IRF2 | 0.441118498 | 6.969535174 | 7.846253086 | 2.20E-14 | 7.76E-14 | 21.5780198 |
| NLRP7 | 0.357221973 | 4.448973068 | 7.678340215 | 7.26E-14 | 2.40E-13 | 20.40273187 |
| NLRP3 | 0.100003494 | 3.826389366 | 7.559369759 | 1.67E-13 | 5.21E-13 | 19.58246097 |
| PYCARD | -0.351681938 | 8.485971099 | -7.470373385 | 3.10E-13 | 9.13E-13 | 18.97567333 |

| | | | | | | |
|---|---|---|---|---|---|---|
| GZMA | 0.530994571 | 5.995783088 | 7.448197687 | 3.61E-13 | 1.01E-12 | 18.82539076 |
| CASP6 | -0.279853953 | 7.351517741 | -7.170384234 | 2.38E-12 | 6.32E-12 | 16.97388591 |
| PRKACA | 0.184867905 | 5.945140269 | 6.535325614 | 1.43E-10 | 3.62E-10 | 12.96378381 |
| GSDMD | 0.204015432 | 7.158637864 | 6.405177053 | 3.19E-10 | 7.69E-10 | 12.18111825 |
| SCAF11 | -0.17501792 | 7.776686925 | -6.274984358 | 7.02E-10 | 1.62E-09 | 11.41179511 |
| BAK1 | 0.246426943 | 6.76250081 | 6.098521308 | 2.00E-09 | 4.42E-09 | 10.39099331 |
| CHMP4B | 0.236544531 | 8.732067571 | 5.898183203 | 6.37E-09 | 1.35E-08 | 9.263022361 |
| BAX | 0.355814128 | 5.636637876 | 5.551126359 | 4.39E-08 | 8.95E-08 | 7.388044522 |
| NOD1 | 0.115036585 | 6.801653725 | 5.318123795 | 1.52E-07 | 2.98E-07 | 6.186392229 |
| APIP | -0.132162839 | 5.342158842 | -4.88504006 | 1.35E-06 | 2.56E-06 | 4.077234191 |
| PLCG1 | 0.092064368 | 7.059553713 | 4.860158321 | 1.53E-06 | 2.79E-06 | 3.961032616 |
| GSDMB | 0.107022201 | 5.331340641 | 4.792971369 | 2.11E-06 | 3.73E-06 | 3.649984787 |
| HMGB1 | -0.112029775 | 10.49026243 | -4.649206725 | 4.16E-06 | 7.12E-06 | 2.99783076 |
| CASP8 | 0.125050178 | 5.864874499 | 4.579715819 | 5.75E-06 | 9.52E-06 | 2.689188814 |
| CHMP7 | 0.109328258 | 7.174982932 | 4.044928401 | 5.97E-05 | 9.59E-05 | 0.459322999 |
| NLRP9 | -0.125059817 | 4.595633998 | -3.824771775 | 0.000145654 | 0.000227048 | -0.382935326 |
| DHX9 | -0.144718533 | 7.156178232 | -3.594756344 | 0.000353438 | 0.000535207 | -1.215073759 |
| CASP5 | 0.068116518 | 5.058479013 | 3.520631284 | 0.000465687 | 0.000685594 | -1.472751404 |
| GPX4 | 0.14484723 | 10.17762815 | 3.269958481 | 0.001141915 | 0.001635716 | -2.306008831 |
| TIRAP | 0.054922132 | 5.29976763 | 3.199984465 | 0.001452403 | 0.002025721 | -2.528045946 |
| CASP1 | 0.093724748 | 8.30379802 | 3.046162308 | 0.002427303 | 0.003298643 | -2.999849185 |
| NLRC4 | 0.050623796 | 3.98937867 | 3.038157401 | 0.002491628 | 0.003301407 | -3.023787035 |
| CASP9 | 0.044312033 | 5.931721889 | 3.006026865 | 0.002765862 | 0.003575383 | -3.119256762 |
| NLRP6 | 0.069716466 | 4.03207328 | 2.884400477 | 0.004072976 | 0.005139708 | -3.471736565 |
| CHMP6 | -0.073655375 | 6.686033002 | -2.510872725 | 0.012324778 | 0.015191006 | -4.465645515 |
| ELANE | 0.051284806 | 4.458036235 | 2.43886544 | 0.015044018 | 0.018121203 | -4.641795414 |

| | | | | | | |
|---|---|---|---|---|---|---|
| CASP4 | -0.082211543 | 7.086383147 | -2.280957765 | 0.022926848 | 0.027002732 | -5.01049949 |
| TP63 | -0.119790614 | 7.214297709 | -2.16599317 | 0.030734323 | 0.035411285 | -5.263697813 |
| NLRP1 | -0.060098184 | 6.43197677 | -2.153882686 | 0.031676767 | 0.035720609 | -5.289620797 |
| CHMP2A | -0.13188881 | 9.624202837 | -2.145007535 | 0.032383118 | 0.035756359 | -5.308527652 |
| NAIP | 0.084937848 | 5.737104632 | 2.0624792 | 0.039624228 | 0.042858858 | -5.480659869 |
| TREM2 | 0.083858311 | 6.100085452 | 2.029804086 | 0.042850226 | 0.045421239 | -5.54697387 |
| TP53 | -0.037683517 | 6.604711138 | -0.71640703 | 0.474039659 | 0.49262945 | -7.341724629 |
| TNF | 0.017000249 | 5.246058086 | 0.625801786 | 0.531700737 | 0.541925751 | -7.402439697 |
| NLRP2 | 0.009323966 | 4.515350461 | 0.189969794 | 0.84940186 | 0.84940186 | -7.580020784 |

**Table S3. Correlations between pyroptosis-related genes in all samples**

| Gene1 | Gene2 | cor | pvalue |
|---|---|---|---|
| AIM2 | APIP | -0.170338315 | 5.33E-05 |
| AIM2 | BAX | 0.114030767 | 0.00706067 |
| AIM2 | CASP1 | 0.544084291 | 3.08E-44 |
| AIM2 | CASP3 | 0.622563033 | 4.36E-61 |
| AIM2 | CASP4 | 0.217408933 | 2.20E-07 |
| AIM2 | CASP5 | 0.221094292 | 1.35E-07 |
| AIM2 | CASP6 | -0.165531421 | 8.67E-05 |
| AIM2 | CASP8 | 0.327231976 | 2.28E-15 |
| AIM2 | CASP9 | 0.107695276 | 0.010978236 |
| AIM2 | CHMP2B | -0.303826924 | 2.33E-13 |
| AIM2 | CHMP4C | -0.381647872 | 9.37E-21 |
| AIM2 | CHMP6 | -0.284785581 | 7.47E-12 |
| AIM2 | CHMP7 | 0.171341444 | 4.81E-05 |
| AIM2 | CYCS | -0.203421884 | 1.29E-06 |

| | | | |
|---|---|---|---|
| AIM2 | DHX9 | -0.172412841 | 4.30E-05 |
| AIM2 | GSDMB | 0.274989042 | 4.03E-11 |
| AIM2 | GSDMC | -0.378447039 | 2.08E-20 |
| AIM2 | GSDMD | 0.3279171 | 1.98E-15 |
| AIM2 | GZMB | 0.585529555 | 1.46E-52 |
| AIM2 | IL18 | -0.345903329 | 4.24E-17 |
| AIM2 | IL1A | 0.455010338 | 8.16E-30 |
| AIM2 | IL1B | 0.531829412 | 5.43E-42 |
| AIM2 | IL6 | 0.370524678 | 1.44E-19 |
| AIM2 | IRF1 | 0.592920631 | 3.55E-54 |
| AIM2 | NAIP | 0.288581638 | 3.82E-12 |
| AIM2 | NLRC4 | 0.21131082 | 4.83E-07 |
| AIM2 | NLRP1 | -0.253653317 | 1.26E-09 |
| AIM2 | NLRP3 | 0.402439067 | 4.24E-23 |
| AIM2 | NLRP6 | 0.112338344 | 0.007960778 |
| AIM2 | NLRP7 | 0.303603812 | 2.43E-13 |
| AIM2 | NLRP9 | -0.145573689 | 0.000568278 |
| AIM2 | NOD2 | -0.296682625 | 8.82E-13 |
| AIM2 | PYCARD | -0.341841126 | 1.03E-16 |
| AIM2 | TP53 | -0.415591249 | 1.14E-24 |
| AIM2 | TP63 | -0.383532397 | 5.83E-21 |
| AIM2 | GZMA | 0.520678682 | 5.02E-40 |
| AIM2 | ZBP1 | 0.512410108 | 1.29E-38 |
| APIP | BAK1 | -0.37725813 | 2.79E-20 |
| APIP | BAX | 0.094279422 | 0.02607898 |
| APIP | CASP1 | 0.142710609 | 0.000730604 |

| | | | |
|---|---|---|---|
| APIP | CASP4 | 0.379691906 | 1.53E-20 |
| APIP | CASP5 | 0.084178605 | 0.047061208 |
| APIP | CASP6 | 0.224940011 | 8.06E-08 |
| APIP | CHMP2A | 0.189952138 | 6.36E-06 |
| APIP | CHMP2B | 0.476380591 | 6.74E-33 |
| APIP | CHMP4C | 0.392213982 | 6.33E-22 |
| APIP | CHMP6 | -0.197805992 | 2.55E-06 |
| APIP | CHMP7 | -0.188919691 | 7.15E-06 |
| APIP | CYCS | 0.295180312 | 1.16E-12 |
| APIP | DHX9 | 0.156786819 | 0.000203213 |
| APIP | ELANE | 0.106832123 | 0.01163954 |
| APIP | GPX4 | -0.382985761 | 6.70E-21 |
| APIP | GSDMB | 0.190578086 | 5.92E-06 |
| APIP | GSDMC | 0.109619425 | 0.009622548 |
| APIP | GSDMD | -0.299178878 | 5.56E-13 |
| APIP | GZMB | -0.283307908 | 9.67E-12 |
| APIP | HMGB1 | 0.218492851 | 1.91E-07 |
| APIP | IL18 | 0.256783093 | 7.74E-10 |
| APIP | IL1B | -0.373270297 | 7.41E-20 |
| APIP | IL6 | -0.146149975 | 0.000539954 |
| APIP | IRF1 | -0.241090706 | 8.29E-09 |
| APIP | NAIP | 0.193646135 | 4.15E-06 |
| APIP | NLRC4 | 0.096943214 | 0.022126231 |
| APIP | NLRP1 | -0.313205238 | 3.83E-14 |
| APIP | NLRP3 | -0.243760641 | 5.60E-09 |
| APIP | NLRP6 | -0.30244722 | 3.02E-13 |

| | | | |
|---|---|---|---|
| APIP | NLRP9 | -0.219547769 | 1.66E-07 |
| APIP | NOD1 | -0.495437524 | 7.75E-36 |
| APIP | NOD2 | 0.436934082 | 2.26E-27 |
| APIP | PLCG1 | -0.475018625 | 1.08E-32 |
| APIP | PRKACA | -0.289623316 | 3.17E-12 |
| APIP | SCAF11 | 0.244596864 | 4.95E-09 |
| APIP | TREM2 | -0.424268449 | 9.58E-26 |
| APIP | TNF | -0.266232927 | 1.72E-10 |
| APIP | GZMA | 0.165802427 | 8.44E-05 |
| APIP | ZBP1 | -0.281748693 | 1.27E-11 |
| BAK1 | BAX | 0.275208198 | 3.88E-11 |
| BAK1 | CASP1 | -0.22425679 | 8.84E-08 |
| BAK1 | CASP4 | -0.281387276 | 1.35E-11 |
| BAK1 | CASP6 | -0.463806142 | 4.67E-31 |
| BAK1 | CASP8 | -0.143136165 | 0.000704026 |
| BAK1 | CHMP2A | -0.135540002 | 0.00134369 |
| BAK1 | CHMP2B | -0.527011392 | 3.92E-41 |
| BAK1 | CHMP4B | 0.168091656 | 6.70E-05 |
| BAK1 | CHMP4C | -0.428622021 | 2.69E-26 |
| BAK1 | CHMP6 | 0.427205635 | 4.08E-26 |
| BAK1 | CHMP7 | 0.175878571 | 2.99E-05 |
| BAK1 | CYCS | -0.095190267 | 0.024663835 |
| BAK1 | DHX9 | -0.093078585 | 0.028051249 |
| BAK1 | ELANE | 0.232093428 | 3.01E-08 |
| BAK1 | GPX4 | 0.393495312 | 4.53E-22 |
| BAK1 | GSDMC | -0.189592698 | 6.62E-06 |

| | | | |
|---|---|---|---|
| BAK1 | GSDMD | 0.220398148 | 1.48E-07 |
| BAK1 | GZMB | 0.156618064 | 0.000206491 |
| BAK1 | HMGB1 | -0.488119226 | 1.10E-34 |
| BAK1 | IL18 | -0.360972638 | 1.39E-18 |
| BAK1 | IL1B | 0.237259513 | 1.44E-08 |
| BAK1 | IL6 | 0.155089499 | 0.000238525 |
| BAK1 | IRF1 | 0.269348321 | 1.03E-10 |
| BAK1 | IRF2 | 0.28129724 | 1.37E-11 |
| BAK1 | NAIP | -0.208887227 | 6.56E-07 |
| BAK1 | NLRP3 | 0.217116982 | 2.29E-07 |
| BAK1 | NLRP6 | 0.196194764 | 3.08E-06 |
| BAK1 | NLRP7 | 0.133695835 | 0.001564449 |
| BAK1 | NLRP9 | -0.086742141 | 0.040712355 |
| BAK1 | NOD1 | 0.142605674 | 0.000737299 |
| BAK1 | PLCG1 | 0.293733699 | 1.51E-12 |
| BAK1 | PRKACA | 0.500589912 | 1.15E-36 |
| BAK1 | PYCARD | 0.192520753 | 4.73E-06 |
| BAK1 | SCAF11 | -0.386610466 | 2.67E-21 |
| BAK1 | TP53 | 0.379464197 | 1.62E-20 |
| BAK1 | TP63 | 0.103779416 | 0.014269778 |
| BAK1 | TREM2 | 0.189551879 | 6.65E-06 |
| BAK1 | TIRAP | 0.124104969 | 0.003348919 |
| BAK1 | TNF | 0.132404248 | 0.001738385 |
| BAK1 | GZMA | -0.130446829 | 0.002035995 |
| BAK1 | ZBP1 | 0.364030506 | 6.77E-19 |
| BAX | CASP1 | 0.088356619 | 0.03709789 |

| | | | |
|---|---|---|---|
| BAX | CASP3 | 0.22986165 | 4.10E-08 |
| BAX | CASP4 | 0.22432048 | 8.77E-08 |
| BAX | CASP6 | -0.527543227 | 3.16E-41 |
| BAX | CASP8 | -0.32003291 | 9.90E-15 |
| BAX | CASP9 | 0.229402426 | 4.37E-08 |
| BAX | CHMP2A | 0.367941505 | 2.68E-19 |
| BAX | CHMP2B | -0.421222342 | 2.30E-25 |
| BAX | CHMP4B | 0.722252652 | 6.31E-91 |
| BAX | CHMP4C | -0.334201291 | 5.32E-16 |
| BAX | CHMP6 | 0.311639552 | 5.20E-14 |
| BAX | CHMP7 | 0.176016608 | 2.95E-05 |
| BAX | CYCS | -0.174090624 | 3.61E-05 |
| BAX | DHX9 | 0.187967071 | 7.97E-06 |
| BAX | ELANE | 0.547841563 | 6.05E-45 |
| BAX | GPX4 | 0.261337712 | 3.77E-10 |
| BAX | GSDMB | 0.389939214 | 1.14E-21 |
| BAX | GSDMC | -0.179689731 | 1.99E-05 |
| BAX | GSDMD | 0.373831232 | 6.47E-20 |
| BAX | GZMB | 0.164791482 | 9.34E-05 |
| BAX | HMGB1 | -0.510094796 | 3.16E-38 |
| BAX | IL18 | -0.503730635 | 3.56E-37 |
| BAX | IL1A | 0.111784328 | 0.008276947 |
| BAX | IL1B | 0.236139703 | 1.70E-08 |
| BAX | IL6 | 0.222206924 | 1.17E-07 |
| BAX | IRF1 | 0.317181069 | 1.75E-14 |
| BAX | IRF2 | 0.166647753 | 7.75E-05 |

| | | | |
|---|---|---|---|
| BAX | NAIP | 0.153348108 | 0.000280665 |
| BAX | NLRC4 | 0.386791848 | 2.55E-21 |
| BAX | NLRP1 | -0.482091574 | 9.27E-34 |
| BAX | NLRP3 | 0.184793215 | 1.14E-05 |
| BAX | NLRP6 | 0.157734885 | 0.000185684 |
| BAX | NLRP7 | 0.569840523 | 2.85E-49 |
| BAX | NLRP9 | -0.648367353 | 9.75E-68 |
| BAX | NOD1 | -0.189152946 | 6.96E-06 |
| BAX | NOD2 | 0.086954857 | 0.040219726 |
| BAX | PLCG1 | 0.188962085 | 7.12E-06 |
| BAX | PRKACA | 0.262467151 | 3.15E-10 |
| BAX | PYCARD | 0.205144017 | 1.05E-06 |
| BAX | SCAF11 | -0.208256295 | 7.11E-07 |
| BAX | TP53 | 0.184875068 | 1.13E-05 |
| BAX | TREM2 | -0.372051031 | 9.97E-20 |
| BAX | TIRAP | -0.246482877 | 3.74E-09 |
| BAX | TNF | -0.162853501 | 0.000113087 |
| BAX | GZMA | 0.254923859 | 1.03E-09 |
| BAX | ZBP1 | 0.505986301 | 1.52E-37 |
| CASP1 | CASP3 | 0.407912243 | 9.60E-24 |
| CASP1 | CASP4 | 0.399302285 | 9.81E-23 |
| CASP1 | CASP5 | 0.264256226 | 2.36E-10 |
| CASP1 | CASP6 | 0.140490815 | 0.000884955 |
| CASP1 | CASP8 | 0.413711452 | 1.93E-24 |
| CASP1 | CASP9 | 0.158688517 | 0.000169489 |
| CASP1 | CHMP2A | 0.10698854 | 0.011517149 |

| | | | |
|---|---|---|---|
| CASP1 | CHMP4C | -0.089880371 | 0.033940193 |
| CASP1 | CHMP6 | -0.397909715 | 1.42E-22 |
| CASP1 | CHMP7 | -0.172820378 | 4.12E-05 |
| CASP1 | CYCS | -0.096381197 | 0.022913964 |
| CASP1 | DHX9 | 0.126160163 | 0.002856957 |
| CASP1 | ELANE | -0.128851947 | 0.002312192 |
| CASP1 | GPX4 | -0.189733438 | 6.52E-06 |
| CASP1 | GSDMB | 0.146238247 | 0.000535733 |
| CASP1 | GZMB | 0.301658313 | 3.50E-13 |
| CASP1 | HMGB1 | 0.221233781 | 1.33E-07 |
| CASP1 | IL18 | 0.095967138 | 0.023509755 |
| CASP1 | IL1A | 0.323711917 | 4.70E-15 |
| CASP1 | IL1B | 0.258052949 | 6.35E-10 |
| CASP1 | IL6 | 0.102431779 | 0.015588497 |
| CASP1 | IRF1 | 0.2344076 | 2.17E-08 |
| CASP1 | IRF2 | -0.113547046 | 0.007308145 |
| CASP1 | NAIP | 0.323787082 | 4.63E-15 |
| CASP1 | NLRP1 | -0.205803338 | 9.64E-07 |
| CASP1 | NLRP2 | 0.148529718 | 0.000436324 |
| CASP1 | NLRP3 | 0.107982224 | 0.010765885 |
| CASP1 | NLRP7 | 0.088627453 | 0.036519062 |
| CASP1 | NOD2 | -0.116711376 | 0.005820432 |
| CASP1 | PLCG1 | -0.152788541 | 0.00029562 |
| CASP1 | PRKACA | -0.289035341 | 3.52E-12 |
| CASP1 | PYCARD | -0.262215716 | 3.28E-10 |
| CASP1 | SCAF11 | 0.228267282 | 5.11E-08 |

| | | | |
|---|---|---|---|
| CASP1 | TP53 | -0.38784232 | 1.95E-21 |
| CASP1 | TREM2 | -0.09111884 | 0.031545307 |
| CASP1 | TNF | -0.091558093 | 0.030731359 |
| CASP1 | GZMA | 0.511325887 | 1.97E-38 |
| CASP1 | ZBP1 | 0.15431122 | 0.000256569 |
| CASP3 | CASP4 | 0.207791038 | 7.53E-07 |
| CASP3 | CASP5 | 0.2298748 | 4.10E-08 |
| CASP3 | CASP6 | -0.264528562 | 2.26E-10 |
| CASP3 | CASP8 | 0.334690667 | 4.80E-16 |
| CASP3 | CASP9 | 0.229110877 | 4.55E-08 |
| CASP3 | CHMP2B | -0.310216372 | 6.86E-14 |
| CASP3 | CHMP4B | 0.195891243 | 3.19E-06 |
| CASP3 | CHMP4C | -0.350772004 | 1.43E-17 |
| CASP3 | CHMP6 | -0.270637421 | 8.33E-11 |
| CASP3 | CHMP7 | 0.144469602 | 0.000626436 |
| CASP3 | CYCS | -0.146676803 | 0.000515213 |
| CASP3 | DHX9 | -0.155811751 | 0.000222849 |
| CASP3 | ELANE | 0.091932383 | 0.030052046 |
| CASP3 | GPX4 | -0.09656418 | 0.022654867 |
| CASP3 | GSDMB | 0.369432953 | 1.87E-19 |
| CASP3 | GSDMC | -0.304934291 | 1.89E-13 |
| CASP3 | GSDMD | 0.228454381 | 4.98E-08 |
| CASP3 | GZMB | 0.344062674 | 6.35E-17 |
| CASP3 | IL18 | -0.420513838 | 2.82E-25 |
| CASP3 | IL1A | 0.410369471 | 4.88E-24 |
| CASP3 | IL1B | 0.486094436 | 2.26E-34 |

| | | | |
|---|---|---|---|
| CASP3 | IL6 | 0.377773258 | 2.46E-20 |
| CASP3 | IRF1 | 0.466689788 | 1.79E-31 |
| CASP3 | IRF2 | 0.177794531 | 2.44E-05 |
| CASP3 | NAIP | 0.243932343 | 5.46E-09 |
| CASP3 | NLRC4 | 0.27374586 | 4.96E-11 |
| CASP3 | NLRP1 | -0.461722899 | 9.26E-31 |
| CASP3 | NLRP3 | 0.380573559 | 1.23E-20 |
| CASP3 | NLRP6 | 0.130014401 | 0.002107733 |
| CASP3 | NLRP7 | 0.453966995 | 1.14E-29 |
| CASP3 | NLRP9 | -0.224823938 | 8.19E-08 |
| CASP3 | NOD1 | -0.144493366 | 0.000625128 |
| CASP3 | NOD2 | -0.233577675 | 2.44E-08 |
| CASP3 | PLCG1 | -0.087690056 | 0.038555736 |
| CASP3 | PYCARD | -0.468608262 | 9.44E-32 |
| CASP3 | SCAF11 | -0.17949138 | 2.03E-05 |
| CASP3 | TP53 | -0.377388916 | 2.70E-20 |
| CASP3 | TP63 | -0.322605955 | 5.89E-15 |
| CASP3 | GZMA | 0.383032059 | 6.62E-21 |
| CASP3 | ZBP1 | 0.596004649 | 7.32E-55 |
| CASP4 | CASP8 | -0.122501147 | 0.003784964 |
| CASP4 | CASP9 | 0.183268833 | 1.35E-05 |
| CASP4 | CHMP2A | 0.415166495 | 1.28E-24 |
| CASP4 | CHMP2B | 0.249735628 | 2.29E-09 |
| CASP4 | CHMP4B | 0.131951246 | 0.001803475 |
| CASP4 | CHMP4C | 0.14897874 | 0.000418978 |
| CASP4 | CHMP6 | -0.166326164 | 8.01E-05 |

| | | | |
|---|---|---|---|
| CASP4 | CHMP7 | 0.103861269 | 0.014192941 |
| CASP4 | ELANE | 0.091408833 | 0.031005901 |
| CASP4 | GPX4 | -0.263611095 | 2.62E-10 |
| CASP4 | GSDMB | 0.158263002 | 0.000176544 |
| CASP4 | GZMB | 0.109521753 | 0.0096876 |
| CASP4 | IL1A | 0.192191062 | 4.92E-06 |
| CASP4 | IL6 | 0.088634297 | 0.036504535 |
| CASP4 | NAIP | 0.20227326 | 1.49E-06 |
| CASP4 | NLRC4 | 0.289652201 | 3.15E-12 |
| CASP4 | NLRP1 | -0.465902306 | 2.33E-31 |
| CASP4 | NLRP3 | -0.114117174 | 0.007017263 |
| CASP4 | NLRP7 | 0.212844158 | 3.97E-07 |
| CASP4 | NLRP9 | -0.326018166 | 2.93E-15 |
| CASP4 | NOD1 | -0.307936961 | 1.06E-13 |
| CASP4 | NOD2 | 0.180179958 | 1.89E-05 |
| CASP4 | PLCG1 | -0.292223028 | 1.99E-12 |
| CASP4 | PRKACA | -0.185600171 | 1.04E-05 |
| CASP4 | PYCARD | -0.104532479 | 0.013576474 |
| CASP4 | SCAF11 | 0.202809791 | 1.39E-06 |
| CASP4 | TP53 | -0.337201714 | 2.81E-16 |
| CASP4 | TP63 | -0.154008319 | 0.00026393 |
| CASP4 | TREM2 | -0.524365827 | 1.14E-40 |
| CASP4 | TIRAP | -0.401252683 | 5.83E-23 |
| CASP4 | TNF | -0.095512843 | 0.024178821 |
| CASP4 | GZMA | 0.243619375 | 5.72E-09 |
| CASP5 | CASP6 | 0.083148826 | 0.049835671 |

| | | | |
|---|---|---|---|
| CASP5 | CASP8 | 0.202321797 | 1.48E-06 |
| CASP5 | CHMP2A | -0.126089782 | 0.002872653 |
| CASP5 | CHMP4C | -0.207910069 | 7.42E-07 |
| CASP5 | CHMP6 | -0.174509071 | 3.46E-05 |
| CASP5 | GPX4 | -0.121355921 | 0.004127151 |
| CASP5 | GSDMB | 0.161944428 | 0.000123633 |
| CASP5 | GSDMC | -0.09401477 | 0.026503064 |
| CASP5 | GSDMD | 0.132924488 | 0.001666296 |
| CASP5 | GZMB | 0.185462516 | 1.06E-05 |
| CASP5 | IL1A | 0.239729053 | 1.01E-08 |
| CASP5 | IL1B | 0.209771977 | 5.87E-07 |
| CASP5 | IL6 | 0.119003908 | 0.004919114 |
| CASP5 | IRF1 | 0.158934388 | 0.000165534 |
| CASP5 | NAIP | 0.192375348 | 4.81E-06 |
| CASP5 | NLRC4 | 0.093708571 | 0.027001126 |
| CASP5 | NOD2 | -0.108326788 | 0.010515715 |
| CASP5 | PLCG1 | -0.18139013 | 1.65E-05 |
| CASP5 | PYCARD | -0.137824258 | 0.001110054 |
| CASP5 | TP53 | -0.215403856 | 2.86E-07 |
| CASP5 | TP63 | -0.146839542 | 0.000507786 |
| CASP5 | TREM2 | 0.093536057 | 0.027285268 |
| CASP5 | TIRAP | 0.181031501 | 1.72E-05 |
| CASP5 | GZMA | 0.210334459 | 5.47E-07 |
| CASP5 | ZBP1 | 0.14477891 | 0.000609609 |
| CASP6 | CASP8 | 0.217637761 | 2.13E-07 |
| CASP6 | CHMP2B | 0.544614005 | 2.45E-44 |

| | | | |
|---|---|---|---|
| CASP6 | CHMP4B | -0.449426677 | 4.81E-29 |
| CASP6 | CHMP4C | 0.469517492 | 6.96E-32 |
| CASP6 | CHMP6 | -0.351899246 | 1.11E-17 |
| CASP6 | CHMP7 | -0.391667841 | 7.29E-22 |
| CASP6 | CYCS | 0.340202169 | 1.47E-16 |
| CASP6 | ELANE | -0.421280478 | 2.26E-25 |
| CASP6 | GPX4 | -0.338641518 | 2.06E-16 |
| CASP6 | GSDMB | -0.100633827 | 0.017513716 |
| CASP6 | GSDMC | 0.315821164 | 2.29E-14 |
| CASP6 | GSDMD | -0.387597319 | 2.08E-21 |
| CASP6 | GZMB | -0.193395623 | 4.28E-06 |
| CASP6 | HMGB1 | 0.663220201 | 7.23E-72 |
| CASP6 | IL18 | 0.675848864 | 1.43E-75 |
| CASP6 | IL1B | -0.37241286 | 9.13E-20 |
| CASP6 | IL6 | -0.345273813 | 4.87E-17 |
| CASP6 | IRF1 | -0.46917823 | 7.80E-32 |
| CASP6 | IRF2 | -0.363413647 | 7.83E-19 |
| CASP6 | NLRC4 | -0.363255341 | 8.13E-19 |
| CASP6 | NLRP1 | 0.299184843 | 5.56E-13 |
| CASP6 | NLRP3 | -0.349928333 | 1.73E-17 |
| CASP6 | NLRP6 | -0.272097117 | 6.54E-11 |
| CASP6 | NLRP7 | -0.426661933 | 4.78E-26 |
| CASP6 | NLRP9 | 0.390557947 | 9.71E-22 |
| CASP6 | NOD1 | -0.122800017 | 0.0037 |
| CASP6 | PLCG1 | -0.29708696 | 8.19E-13 |
| CASP6 | PRKACA | -0.515330619 | 4.14E-39 |

| | | | |
|---|---|---|---|
| CASP6 | SCAF11 | 0.576077692 | 1.47E-50 |
| CASP6 | TP53 | -0.277623468 | 2.58E-11 |
| CASP6 | TREM2 | 0.088919932 | 0.035902612 |
| CASP6 | TNF | -0.105982884 | 0.01232434 |
| CASP6 | GZMA | -0.158964985 | 0.000165048 |
| CASP6 | ZBP1 | -0.576405045 | 1.26E-50 |
| CASP8 | CHMP2A | -0.370532165 | 1.44E-19 |
| CASP8 | CHMP4B | -0.183472088 | 1.32E-05 |
| CASP8 | CHMP6 | -0.400758285 | 6.65E-23 |
| CASP8 | CHMP7 | -0.290487578 | 2.72E-12 |
| CASP8 | CYCS | 0.091669897 | 0.030527076 |
| CASP8 | DHX9 | 0.134100979 | 0.001513275 |
| CASP8 | ELANE | -0.35549713 | 4.91E-18 |
| CASP8 | GPX4 | -0.276512996 | 3.11E-11 |
| CASP8 | GSDMD | -0.292802333 | 1.79E-12 |
| CASP8 | GZMB | 0.25096381 | 1.90E-09 |
| CASP8 | HMGB1 | 0.446130071 | 1.35E-28 |
| CASP8 | IL18 | 0.08954635 | 0.034612049 |
| CASP8 | IL1A | 0.227173506 | 5.94E-08 |
| CASP8 | IL1B | 0.185255475 | 1.08E-05 |
| CASP8 | IL6 | 0.097426183 | 0.021468168 |
| CASP8 | IRF1 | 0.090285791 | 0.033139692 |
| CASP8 | IRF2 | -0.093849194 | 0.026771397 |
| CASP8 | NAIP | 0.162864546 | 0.000112964 |
| CASP8 | NLRC4 | -0.210602241 | 5.29E-07 |
| CASP8 | NLRP1 | 0.189512757 | 6.68E-06 |

| | | | |
|---|---|---|---|
| CASP8 | NLRP3 | 0.132932647 | 0.001665188 |
| CASP8 | NLRP7 | -0.203675327 | 1.25E-06 |
| CASP8 | NLRP9 | 0.390093298 | 1.10E-21 |
| CASP8 | NOD1 | 0.166252206 | 8.07E-05 |
| CASP8 | NOD2 | -0.256253772 | 8.41E-10 |
| CASP8 | PLCG1 | -0.191125337 | 5.56E-06 |
| CASP8 | PRKACA | -0.317929372 | 1.51E-14 |
| CASP8 | PYCARD | -0.426124348 | 5.59E-26 |
| CASP8 | SCAF11 | 0.147336554 | 0.000485717 |
| CASP8 | TP53 | -0.282516367 | 1.11E-11 |
| CASP8 | TP63 | 0.085148173 | 0.044568564 |
| CASP8 | TREM2 | 0.383023074 | 6.63E-21 |
| CASP8 | TIRAP | 0.367475104 | 2.99E-19 |
| CASP8 | GZMA | 0.244604086 | 4.95E-09 |
| CASP9 | CHMP2A | 0.211097062 | 4.97E-07 |
| CASP9 | CHMP2B | -0.139882501 | 0.000932234 |
| CASP9 | CHMP4B | 0.209523592 | 6.06E-07 |
| CASP9 | CHMP4C | -0.112353461 | 0.007952305 |
| CASP9 | CHMP6 | 0.086864806 | 0.040427657 |
| CASP9 | CHMP7 | 0.103302748 | 0.014724549 |
| CASP9 | GPX4 | 0.094309065 | 0.026031845 |
| CASP9 | GSDMD | 0.090100983 | 0.033502573 |
| CASP9 | GZMB | 0.188298577 | 7.67E-06 |
| CASP9 | HMGB1 | -0.149103002 | 0.000414293 |
| CASP9 | IL1A | 0.207968319 | 7.37E-07 |
| CASP9 | IL1B | 0.233726963 | 2.39E-08 |

| | | | |
|---|---|---|---|
| CASP9 | IL6 | 0.112668434 | 0.00777757 |
| CASP9 | IRF1 | 0.121075419 | 0.004215122 |
| CASP9 | NAIP | -0.111193775 | 0.00862626 |
| CASP9 | NLRC4 | 0.116803664 | 0.005781458 |
| CASP9 | NLRP1 | -0.148475898 | 0.000438447 |
| CASP9 | NLRP3 | 0.127336004 | 0.002606026 |
| CASP9 | NLRP6 | 0.128417559 | 0.002393133 |
| CASP9 | NLRP7 | 0.18653236 | 9.36E-06 |
| CASP9 | NLRP9 | -0.145312268 | 0.000581577 |
| CASP9 | PYCARD | -0.190737981 | 5.81E-06 |
| CASP9 | SCAF11 | -0.091459379 | 0.030912694 |
| CASP9 | TREM2 | -0.13121926 | 0.001913386 |
| CASP9 | TIRAP | -0.107565149 | 0.011075751 |
| CASP9 | ZBP1 | 0.123334607 | 0.003552326 |
| CHMP2A | CHMP2B | 0.104256279 | 0.013827232 |
| CHMP2A | CHMP4B | 0.166375653 | 7.97E-05 |
| CHMP2A | CHMP4C | 0.140996878 | 0.000847324 |
| CHMP2A | CHMP6 | 0.094060863 | 0.026428779 |
| CHMP2A | CHMP7 | 0.116975898 | 0.00570935 |
| CHMP2A | ELANE | 0.20012687 | 1.93E-06 |
| CHMP2A | GSDMD | 0.152801985 | 0.000295252 |
| CHMP2A | HMGB1 | -0.151874895 | 0.000321647 |
| CHMP2A | IL1B | -0.100987493 | 0.017119372 |
| CHMP2A | IL6 | -0.124224174 | 0.003318406 |
| CHMP2A | IRF1 | -0.190418975 | 6.03E-06 |
| CHMP2A | IRF2 | -0.335697411 | 3.87E-16 |

| | | | |
|---|---|---|---|
| CHMP2A | NLRC4 | 0.17716785 | 2.61E-05 |
| CHMP2A | NLRP1 | -0.374088087 | 6.07E-20 |
| CHMP2A | NLRP3 | -0.103730706 | 0.014315677 |
| CHMP2A | NLRP7 | 0.27900869 | 2.03E-11 |
| CHMP2A | NLRP9 | -0.400629223 | 6.89E-23 |
| CHMP2A | NOD1 | -0.299097927 | 5.65E-13 |
| CHMP2A | NOD2 | 0.13120598 | 0.001915435 |
| CHMP2A | PRKACA | -0.165034502 | 9.11E-05 |
| CHMP2A | PYCARD | 0.275721693 | 3.56E-11 |
| CHMP2A | SCAF11 | 0.093997818 | 0.02653043 |
| CHMP2A | TP53 | -0.201589749 | 1.62E-06 |
| CHMP2A | TP63 | -0.141113346 | 0.000838875 |
| CHMP2A | TREM2 | -0.4262444 | 5.40E-26 |
| CHMP2A | TIRAP | -0.544719454 | 2.34E-44 |
| CHMP2A | TNF | -0.156029217 | 0.000218322 |
| CHMP2B | CHMP4B | -0.33011107 | 1.26E-15 |
| CHMP2B | CHMP4C | 0.668278724 | 2.50E-73 |
| CHMP2B | CHMP6 | -0.264365086 | 2.32E-10 |
| CHMP2B | CHMP7 | -0.212682591 | 4.06E-07 |
| CHMP2B | CYCS | 0.432407694 | 8.79E-27 |
| CHMP2B | DHX9 | 0.147698168 | 0.000470223 |
| CHMP2B | ELANE | -0.345044875 | 5.12E-17 |
| CHMP2B | GPX4 | -0.460418313 | 1.42E-30 |
| CHMP2B | GSDMB | -0.264061184 | 2.44E-10 |
| CHMP2B | GSDMC | 0.365387983 | 4.92E-19 |
| CHMP2B | GSDMD | -0.52495173 | 9.03E-41 |

| | | | |
|---|---|---|---|
| CHMP2B | GZMB | -0.356608112 | 3.80E-18 |
| CHMP2B | HMGB1 | 0.514210059 | 6.42E-39 |
| CHMP2B | IL18 | 0.630796676 | 3.85E-63 |
| CHMP2B | IL1B | -0.497527383 | 3.59E-36 |
| CHMP2B | IL6 | -0.23142801 | 3.30E-08 |
| CHMP2B | IRF1 | -0.517315479 | 1.90E-39 |
| CHMP2B | IRF2 | -0.271456725 | 7.27E-11 |
| CHMP2B | NLRC4 | -0.158144041 | 0.000178565 |
| CHMP2B | NLRP3 | -0.391962429 | 6.75E-22 |
| CHMP2B | NLRP6 | -0.288354813 | 3.98E-12 |
| CHMP2B | NLRP7 | -0.376798832 | 3.12E-20 |
| CHMP2B | NLRP9 | 0.18811832 | 7.83E-06 |
| CHMP2B | NOD1 | -0.242147637 | 7.11E-09 |
| CHMP2B | NOD2 | 0.393019117 | 5.13E-22 |
| CHMP2B | PLCG1 | -0.475286301 | 9.81E-33 |
| CHMP2B | PRKACA | -0.476806588 | 5.82E-33 |
| CHMP2B | SCAF11 | 0.477175862 | 5.12E-33 |
| CHMP2B | TP53 | -0.196103921 | 3.11E-06 |
| CHMP2B | TREM2 | -0.236746252 | 1.55E-08 |
| CHMP2B | TIRAP | -0.192818919 | 4.57E-06 |
| CHMP2B | GZMA | -0.151257789 | 0.00034042 |
| CHMP2B | ZBP1 | -0.648633371 | 8.26E-68 |
| CHMP4B | CHMP4C | -0.244578699 | 4.96E-09 |
| CHMP4B | CHMP6 | 0.207187941 | 8.12E-07 |
| CHMP4B | CYCS | -0.188994675 | 7.09E-06 |
| CHMP4B | DHX9 | 0.183198057 | 1.36E-05 |

| | | | |
|---|---|---|---|
| CHMP4B | ELANE | 0.420574589 | 2.77E-25 |
| CHMP4B | GPX4 | 0.1218348 | 0.003980796 |
| CHMP4B | GSDMB | 0.277748684 | 2.52E-11 |
| CHMP4B | GSDMD | 0.224033805 | 9.11E-08 |
| CHMP4B | HMGB1 | -0.399045018 | 1.05E-22 |
| CHMP4B | IL18 | -0.395809823 | 2.47E-22 |
| CHMP4B | IL1A | 0.141128251 | 0.000837799 |
| CHMP4B | IL1B | 0.17972208 | 1.98E-05 |
| CHMP4B | IL6 | 0.147655186 | 0.000472041 |
| CHMP4B | IRF1 | 0.236078395 | 1.71E-08 |
| CHMP4B | IRF2 | 0.167469368 | 7.14E-05 |
| CHMP4B | NAIP | 0.105003727 | 0.013157876 |
| CHMP4B | NLRC4 | 0.196624532 | 2.93E-06 |
| CHMP4B | NLRP1 | -0.329307585 | 1.49E-15 |
| CHMP4B | NLRP6 | 0.108891184 | 0.010117066 |
| CHMP4B | NLRP7 | 0.373781569 | 6.54E-20 |
| CHMP4B | NLRP9 | -0.42575226 | 6.23E-26 |
| CHMP4B | PLCG1 | 0.251036722 | 1.88E-09 |
| CHMP4B | PRKACA | 0.253661215 | 1.26E-09 |
| CHMP4B | SCAF11 | -0.210510524 | 5.35E-07 |
| CHMP4B | TP53 | 0.148949377 | 0.000420093 |
| CHMP4B | TREM2 | -0.22102481 | 1.36E-07 |
| CHMP4B | TIRAP | -0.119119046 | 0.004877361 |
| CHMP4B | TNF | -0.160320344 | 0.000144812 |
| CHMP4B | GZMA | 0.143547028 | 0.000679217 |
| CHMP4B | ZBP1 | 0.392993909 | 5.16E-22 |

| | | | |
|---|---|---|---|
| CHMP4C | CHMP6 | -0.139446106 | 0.000967577 |
| CHMP4C | CHMP7 | -0.242490795 | 6.76E-09 |
| CHMP4C | CYCS | 0.376734491 | 3.17E-20 |
| CHMP4C | DHX9 | 0.104744963 | 0.013386298 |
| CHMP4C | ELANE | -0.204774101 | 1.10E-06 |
| CHMP4C | GPX4 | -0.355216642 | 5.23E-18 |
| CHMP4C | GSDMB | -0.285096554 | 7.07E-12 |
| CHMP4C | GSDMC | 0.372774847 | 8.36E-20 |
| CHMP4C | GSDMD | -0.530224423 | 1.05E-41 |
| CHMP4C | GZMB | -0.389792506 | 1.18E-21 |
| CHMP4C | HMGB1 | 0.349443197 | 1.93E-17 |
| CHMP4C | IL18 | 0.585516348 | 1.47E-52 |
| CHMP4C | IL1A | -0.255370382 | 9.65E-10 |
| CHMP4C | IL1B | -0.562005068 | 1.08E-47 |
| CHMP4C | IL6 | -0.331090966 | 1.02E-15 |
| CHMP4C | IRF1 | -0.581769476 | 9.30E-52 |
| CHMP4C | IRF2 | -0.19753853 | 2.63E-06 |
| CHMP4C | NLRC4 | -0.145950103 | 0.000549625 |
| CHMP4C | NLRP3 | -0.432332315 | 8.99E-27 |
| CHMP4C | NLRP6 | -0.289899602 | 3.02E-12 |
| CHMP4C | NLRP7 | -0.325241481 | 3.44E-15 |
| CHMP4C | NLRP9 | 0.19074483 | 5.81E-06 |
| CHMP4C | NOD1 | -0.195075529 | 3.51E-06 |
| CHMP4C | NOD2 | 0.340339429 | 1.43E-16 |
| CHMP4C | PLCG1 | -0.312584664 | 4.33E-14 |
| CHMP4C | PRKACA | -0.474456954 | 1.30E-32 |

| | | | |
|---|---|---|---|
| CHMP4C | PYCARD | 0.129041938 | 0.002277583 |
| CHMP4C | SCAF11 | 0.322287006 | 6.28E-15 |
| CHMP4C | TP63 | 0.170353124 | 5.32E-05 |
| CHMP4C | TREM2 | -0.147754506 | 0.000467851 |
| CHMP4C | TIRAP | -0.156600808 | 0.000206829 |
| CHMP4C | TNF | -0.18711549 | 8.77E-06 |
| CHMP4C | GZMA | -0.130809639 | 0.001977537 |
| CHMP4C | ZBP1 | -0.66214396 | 1.47E-71 |
| CHMP6 | CHMP7 | 0.199684591 | 2.03E-06 |
| CHMP6 | CYCS | -0.113608621 | 0.007276217 |
| CHMP6 | ELANE | 0.31821211 | 1.42E-14 |
| CHMP6 | GPX4 | 0.379572336 | 1.57E-20 |
| CHMP6 | GSDMB | -0.125410719 | 0.003028172 |
| CHMP6 | GSDMC | 0.113873733 | 0.007140174 |
| CHMP6 | GSDMD | 0.276250124 | 3.25E-11 |
| CHMP6 | GZMB | -0.095411744 | 0.024329935 |
| CHMP6 | HMGB1 | -0.434351047 | 4.92E-27 |
| CHMP6 | IL18 | -0.217249227 | 2.25E-07 |
| CHMP6 | IL1A | -0.332226046 | 8.08E-16 |
| CHMP6 | IRF2 | 0.134302442 | 0.001488405 |
| CHMP6 | NAIP | -0.287614318 | 4.53E-12 |
| CHMP6 | NLRC4 | 0.09299225 | 0.028197861 |
| CHMP6 | NLRP6 | 0.16445151 | 9.66E-05 |
| CHMP6 | NLRP9 | -0.124681139 | 0.003203764 |
| CHMP6 | NOD2 | 0.101006478 | 0.017098425 |
| CHMP6 | PLCG1 | 0.332009295 | 8.45E-16 |

| | | | |
|---|---|---|---|
| CHMP6 | PRKACA | 0.429040591 | 2.38E-26 |
| CHMP6 | PYCARD | 0.352876114 | 8.91E-18 |
| CHMP6 | SCAF11 | -0.335498081 | 4.04E-16 |
| CHMP6 | TP53 | 0.422543695 | 1.58E-25 |
| CHMP6 | TP63 | 0.147208371 | 0.000491322 |
| CHMP6 | TIRAP | -0.154924552 | 0.000242247 |
| CHMP6 | TNF | 0.181705023 | 1.60E-05 |
| CHMP6 | GZMA | -0.285098714 | 7.07E-12 |
| CHMP7 | CYCS | -0.215857672 | 2.69E-07 |
| CHMP7 | DHX9 | -0.372672059 | 8.57E-20 |
| CHMP7 | ELANE | 0.132615213 | 0.001708814 |
| CHMP7 | GPX4 | 0.292711342 | 1.82E-12 |
| CHMP7 | GSDMC | -0.222038992 | 1.19E-07 |
| CHMP7 | GSDMD | 0.337682291 | 2.54E-16 |
| CHMP7 | GZMB | 0.205328044 | 1.02E-06 |
| CHMP7 | HMGB1 | -0.358090125 | 2.71E-18 |
| CHMP7 | IL18 | -0.312020806 | 4.83E-14 |
| CHMP7 | IL1B | 0.200692434 | 1.80E-06 |
| CHMP7 | IL6 | 0.175105095 | 3.25E-05 |
| CHMP7 | IRF1 | 0.305691842 | 1.64E-13 |
| CHMP7 | IRF2 | 0.231974154 | 3.06E-08 |
| CHMP7 | NAIP | -0.106505413 | 0.011898899 |
| CHMP7 | NLRC4 | 0.365540301 | 4.74E-19 |
| CHMP7 | NLRP1 | -0.264911421 | 2.13E-10 |
| CHMP7 | NLRP3 | 0.289670071 | 3.14E-12 |
| CHMP7 | NLRP6 | 0.156165246 | 0.000215534 |

| | | | |
|---|---|---|---|
| CHMP7 | NLRP7 | 0.405919627 | 1.65E-23 |
| CHMP7 | NLRP9 | -0.328086218 | 1.92E-15 |
| CHMP7 | PLCG1 | 0.192655094 | 4.66E-06 |
| CHMP7 | PRKACA | 0.322719435 | 5.75E-15 |
| CHMP7 | SCAF11 | -0.349215267 | 2.03E-17 |
| CHMP7 | TP63 | -0.405957303 | 1.64E-23 |
| CHMP7 | TREM2 | -0.267245184 | 1.46E-10 |
| CHMP7 | TIRAP | -0.248128356 | 2.92E-09 |
| CHMP7 | TNF | 0.261802816 | 3.50E-10 |
| CHMP7 | GZMA | 0.094113951 | 0.026343442 |
| CHMP7 | ZBP1 | 0.280075577 | 1.69E-11 |
| CYCS | DHX9 | 0.234088146 | 2.27E-08 |
| CYCS | ELANE | -0.13966947 | 0.000949335 |
| CYCS | GPX4 | -0.333094845 | 6.73E-16 |
| CYCS | GSDMB | -0.113388991 | 0.007390672 |
| CYCS | GSDMC | 0.222961186 | 1.05E-07 |
| CYCS | GSDMD | -0.421451175 | 2.16E-25 |
| CYCS | GZMB | -0.233983236 | 2.30E-08 |
| CYCS | HMGB1 | 0.370406362 | 1.48E-19 |
| CYCS | IL18 | 0.284800084 | 7.45E-12 |
| CYCS | IL1B | -0.320807064 | 8.47E-15 |
| CYCS | IRF1 | -0.333432536 | 6.26E-16 |
| CYCS | IRF2 | -0.186941774 | 8.94E-06 |
| CYCS | NLRC4 | -0.158219099 | 0.000177288 |
| CYCS | NLRP3 | -0.228580096 | 4.90E-08 |
| CYCS | NLRP6 | -0.188173498 | 7.78E-06 |

| | | | |
|---|---|---|---|
| CYCS | NLRP7 | -0.175313076 | 3.18E-05 |
| CYCS | NLRP9 | 0.147482035 | 0.000479427 |
| CYCS | NOD1 | -0.242225568 | 7.02E-09 |
| CYCS | NOD2 | 0.323363621 | 5.05E-15 |
| CYCS | PLCG1 | -0.286448668 | 5.57E-12 |
| CYCS | PRKACA | -0.351626132 | 1.18E-17 |
| CYCS | SCAF11 | 0.348279119 | 2.50E-17 |
| CYCS | TP63 | 0.083552707 | 0.048731636 |
| CYCS | GZMA | -0.199835858 | 2.00E-06 |
| CYCS | ZBP1 | -0.400139042 | 7.85E-23 |
| DHX9 | GPX4 | -0.175283852 | 3.19E-05 |
| DHX9 | GSDMB | -0.147309629 | 0.000486889 |
| DHX9 | GSDMC | 0.234677051 | 2.09E-08 |
| DHX9 | GSDMD | -0.301163425 | 3.84E-13 |
| DHX9 | GZMB | -0.147836379 | 0.000464424 |
| DHX9 | HMGB1 | 0.213474539 | 3.66E-07 |
| DHX9 | IL1B | -0.100190609 | 0.018019106 |
| DHX9 | IRF1 | -0.25610375 | 8.61E-10 |
| DHX9 | IRF2 | -0.124620901 | 0.003218668 |
| DHX9 | NLRC4 | -0.116611022 | 0.005863081 |
| DHX9 | NLRP3 | -0.169968949 | 5.54E-05 |
| DHX9 | NLRP7 | -0.134106467 | 0.001512593 |
| DHX9 | PLCG1 | -0.091525072 | 0.030791916 |
| DHX9 | PRKACA | -0.209104103 | 6.39E-07 |
| DHX9 | SCAF11 | 0.316986902 | 1.82E-14 |
| DHX9 | TP53 | 0.118932141 | 0.004945302 |

| | | | |
|---|---|---|---|
| DHX9 | TP63 | 0.580924102 | 1.41E-51 |
| DHX9 | TNF | -0.253483456 | 1.29E-09 |
| DHX9 | GZMA | -0.083879416 | 0.047853606 |
| DHX9 | ZBP1 | -0.142929191 | 0.000716838 |
| ELANE | GPX4 | 0.190527472 | 5.95E-06 |
| ELANE | GSDMB | 0.261500406 | 3.68E-10 |
| ELANE | GSDMC | -0.171515771 | 4.72E-05 |
| ELANE | GSDMD | 0.300294914 | 4.52E-13 |
| ELANE | HMGB1 | -0.452223065 | 1.99E-29 |
| ELANE | IL18 | -0.424650297 | 8.58E-26 |
| ELANE | IL1B | 0.09355734 | 0.027250075 |
| ELANE | IL6 | 0.114045612 | 0.007053196 |
| ELANE | IRF1 | 0.16454856 | 9.56E-05 |
| ELANE | IRF2 | 0.242163779 | 7.09E-09 |
| ELANE | NLRC4 | 0.285109891 | 7.06E-12 |
| ELANE | NLRP1 | -0.382431422 | 7.70E-21 |
| ELANE | NLRP3 | 0.161058703 | 0.000134793 |
| ELANE | NLRP6 | 0.135042805 | 0.0014002 |
| ELANE | NLRP7 | 0.427023075 | 4.30E-26 |
| ELANE | NLRP9 | -0.461377442 | 1.04E-30 |
| ELANE | NOD1 | -0.163691832 | 0.000104116 |
| ELANE | NOD2 | 0.135972335 | 0.001296267 |
| ELANE | PLCG1 | 0.122259842 | 0.003854849 |
| ELANE | PRKACA | 0.324779139 | 3.78E-15 |
| ELANE | PYCARD | 0.191100141 | 5.57E-06 |
| ELANE | SCAF11 | -0.301280127 | 3.76E-13 |

| | | | |
|---|---|---|---|
| ELANE | TP53 | 0.251942545 | 1.64E-09 |
| ELANE | TREM2 | -0.232744924 | 2.74E-08 |
| ELANE | TIRAP | -0.108167331 | 0.01063084 |
| ELANE | TNF | -0.117985359 | 0.005302752 |
| ELANE | GZMA | 0.128632643 | 0.002352738 |
| ELANE | ZBP1 | 0.356598136 | 3.81E-18 |
| GPX4 | GSDMC | -0.319109401 | 1.19E-14 |
| GPX4 | GSDMD | 0.389009894 | 1.45E-21 |
| GPX4 | GZMB | 0.161926352 | 0.000123852 |
| GPX4 | HMGB1 | -0.397550275 | 1.56E-22 |
| GPX4 | IL18 | -0.29099673 | 2.48E-12 |
| GPX4 | IL1B | 0.234946294 | 2.01E-08 |
| GPX4 | IRF1 | 0.266513723 | 1.64E-10 |
| GPX4 | IRF2 | 0.215459614 | 2.84E-07 |
| GPX4 | NAIP | -0.083808316 | 0.048043551 |
| GPX4 | NLRP1 | 0.156182173 | 0.000215189 |
| GPX4 | NLRP3 | 0.22955192 | 4.28E-08 |
| GPX4 | NLRP6 | 0.21306922 | 3.86E-07 |
| GPX4 | NLRP7 | 0.201560681 | 1.62E-06 |
| GPX4 | NLRP9 | -0.168420612 | 6.48E-05 |
| GPX4 | NOD1 | 0.258877466 | 5.57E-10 |
| GPX4 | NOD2 | -0.305832778 | 1.59E-13 |
| GPX4 | PLCG1 | 0.343141004 | 7.78E-17 |
| GPX4 | PRKACA | 0.526262773 | 5.31E-41 |
| GPX4 | PYCARD | 0.221383578 | 1.30E-07 |
| GPX4 | SCAF11 | -0.214255809 | 3.31E-07 |

| | | | |
|---|---|---|---|
| GPX4 | TP53 | 0.388414852 | 1.69E-21 |
| GPX4 | TNF | 0.200600836 | 1.82E-06 |
| GPX4 | ZBP1 | 0.32726955 | 2.27E-15 |
| GSDMB | GSDMC | -0.303638585 | 2.41E-13 |
| GSDMB | GSDMD | 0.317278903 | 1.72E-14 |
| GSDMB | GZMB | 0.236898411 | 1.52E-08 |
| GSDMB | HMGB1 | -0.178789198 | 2.19E-05 |
| GSDMB | IL18 | -0.253296082 | 1.33E-09 |
| GSDMB | IL1A | 0.186649368 | 9.24E-06 |
| GSDMB | IL1B | 0.155748071 | 0.000224192 |
| GSDMB | IL6 | 0.084151586 | 0.047132312 |
| GSDMB | IRF1 | 0.39113512 | 8.37E-22 |
| GSDMB | IRF2 | 0.128072584 | 0.002459248 |
| GSDMB | NAIP | 0.401107921 | 6.06E-23 |
| GSDMB | NLRC4 | 0.193090128 | 4.43E-06 |
| GSDMB | NLRP1 | -0.295403518 | 1.11E-12 |
| GSDMB | NLRP3 | 0.094979596 | 0.02498511 |
| GSDMB | NLRP7 | 0.364263364 | 6.41E-19 |
| GSDMB | NLRP9 | -0.363796478 | 7.16E-19 |
| GSDMB | NOD1 | -0.208756744 | 6.67E-07 |
| GSDMB | PRKACA | 0.093748947 | 0.026934992 |
| GSDMB | TP53 | -0.103045358 | 0.014975374 |
| GSDMB | TP63 | -0.330868804 | 1.07E-15 |
| GSDMB | TIRAP | 0.224383456 | 8.69E-08 |
| GSDMB | TNF | -0.148689239 | 0.000430086 |
| GSDMB | GZMA | 0.328643672 | 1.71E-15 |

| | | | |
|---|---|---|---|
| GSDMB | ZBP1 | 0.489755396 | 6.10E-35 |
| GSDMC | GSDMD | -0.232824223 | 2.71E-08 |
| GSDMC | GZMB | -0.284864047 | 7.37E-12 |
| GSDMC | HMGB1 | 0.246493373 | 3.73E-09 |
| GSDMC | IL18 | 0.396334676 | 2.15E-22 |
| GSDMC | IL1A | -0.283087307 | 1.00E-11 |
| GSDMC | IL1B | -0.373213797 | 7.51E-20 |
| GSDMC | IL6 | -0.2452091 | 4.52E-09 |
| GSDMC | IRF1 | -0.534301405 | 1.95E-42 |
| GSDMC | IRF2 | -0.286432053 | 5.59E-12 |
| GSDMC | NAIP | -0.269861414 | 9.47E-11 |
| GSDMC | NLRC4 | -0.218410155 | 1.93E-07 |
| GSDMC | NLRP1 | 0.144910157 | 0.000602597 |
| GSDMC | NLRP2 | 0.091523854 | 0.030794151 |
| GSDMC | NLRP3 | -0.374278159 | 5.80E-20 |
| GSDMC | NLRP6 | -0.142618176 | 0.000736499 |
| GSDMC | NLRP7 | -0.398056422 | 1.37E-22 |
| GSDMC | NLRP9 | 0.267452278 | 1.41E-10 |
| GSDMC | NOD1 | 0.091343827 | 0.031126125 |
| GSDMC | NOD2 | 0.265070037 | 2.07E-10 |
| GSDMC | PRKACA | -0.305861934 | 1.58E-13 |
| GSDMC | SCAF11 | 0.208522435 | 6.87E-07 |
| GSDMC | TP63 | 0.344121931 | 6.27E-17 |
| GSDMC | TIRAP | -0.177181177 | 2.60E-05 |
| GSDMC | GZMA | -0.322356209 | 6.19E-15 |
| GSDMC | ZBP1 | -0.529009719 | 1.73E-41 |

| | | | |
|---|---|---|---|
| GSDMD | GZMB | 0.370669316 | 1.39E-19 |
| GSDMD | HMGB1 | -0.48352069 | 5.61E-34 |
| GSDMD | IL18 | -0.504734715 | 2.44E-37 |
| GSDMD | IL1A | 0.114445843 | 0.006854326 |
| GSDMD | IL1B | 0.336349297 | 3.37E-16 |
| GSDMD | IL6 | 0.182790414 | 1.42E-05 |
| GSDMD | IRF1 | 0.438988069 | 1.21E-27 |
| GSDMD | NLRC4 | 0.27195718 | 6.69E-11 |
| GSDMD | NLRP1 | -0.292921481 | 1.75E-12 |
| GSDMD | NLRP3 | 0.287670212 | 4.49E-12 |
| GSDMD | NLRP6 | 0.241724696 | 7.56E-09 |
| GSDMD | NLRP7 | 0.38821522 | 1.77E-21 |
| GSDMD | NLRP9 | -0.344515651 | 5.75E-17 |
| GSDMD | NOD2 | -0.190966246 | 5.66E-06 |
| GSDMD | PLCG1 | 0.377746902 | 2.47E-20 |
| GSDMD | PRKACA | 0.327362396 | 2.22E-15 |
| GSDMD | SCAF11 | -0.271804888 | 6.86E-11 |
| GSDMD | TP53 | -0.097271985 | 0.021676398 |
| GSDMD | TP63 | -0.352858609 | 8.94E-18 |
| GSDMD | TIRAP | -0.105449417 | 0.012772495 |
| GSDMD | TNF | 0.094601813 | 0.025570288 |
| GSDMD | GZMA | 0.1890956 | 7.01E-06 |
| GSDMD | ZBP1 | 0.518213682 | 1.33E-39 |
| GZMB | HMGB1 | -0.185960468 | 9.98E-06 |
| GZMB | IL18 | -0.324418095 | 4.07E-15 |
| GZMB | IL1A | 0.362977562 | 8.68E-19 |

| | | | |
|---|---|---|---|
| GZMB | IL1B | 0.438714281 | 1.32E-27 |
| GZMB | IL6 | 0.435153144 | 3.87E-27 |
| GZMB | IRF1 | 0.570506826 | 2.08E-49 |
| GZMB | NAIP | 0.220257203 | 1.51E-07 |
| GZMB | NLRC4 | 0.129108579 | 0.002265556 |
| GZMB | NLRP1 | -0.096419377 | 0.022859693 |
| GZMB | NLRP2 | -0.091278561 | 0.031247231 |
| GZMB | NLRP3 | 0.387141229 | 2.34E-21 |
| GZMB | NLRP7 | 0.171561662 | 4.70E-05 |
| GZMB | NLRP9 | -0.142100878 | 0.000770309 |
| GZMB | NOD1 | 0.17613531 | 2.91E-05 |
| GZMB | NOD2 | -0.264238854 | 2.37E-10 |
| GZMB | PLCG1 | 0.096639523 | 0.022548924 |
| GZMB | PYCARD | -0.181445804 | 1.64E-05 |
| GZMB | SCAF11 | -0.111694893 | 0.008329024 |
| GZMB | TP53 | -0.276358378 | 3.19E-11 |
| GZMB | TP63 | -0.298835008 | 5.93E-13 |
| GZMB | TNF | 0.165459405 | 8.74E-05 |
| GZMB | GZMA | 0.499717322 | 1.60E-36 |
| GZMB | ZBP1 | 0.406523762 | 1.40E-23 |
| HMGB1 | IL18 | 0.436147829 | 2.87E-27 |
| HMGB1 | IL1B | -0.261253819 | 3.82E-10 |
| HMGB1 | IL6 | -0.184447073 | 1.18E-05 |
| HMGB1 | IRF1 | -0.381525858 | 9.66E-21 |
| HMGB1 | IRF2 | -0.256907517 | 7.59E-10 |
| HMGB1 | NAIP | 0.102321815 | 0.015700677 |

| | | | |
|---|---|---|---|
| HMGB1 | NLRC4 | -0.283576455 | 9.23E-12 |
| HMGB1 | NLRP1 | 0.188432494 | 7.56E-06 |
| HMGB1 | NLRP3 | -0.249008623 | 2.56E-09 |
| HMGB1 | NLRP6 | -0.164551399 | 9.56E-05 |
| HMGB1 | NLRP7 | -0.323135716 | 5.29E-15 |
| HMGB1 | NLRP9 | 0.424715602 | 8.41E-26 |
| HMGB1 | PLCG1 | -0.325997842 | 2.95E-15 |
| HMGB1 | PRKACA | -0.551711414 | 1.11E-45 |
| HMGB1 | PYCARD | -0.223553772 | 9.72E-08 |
| HMGB1 | SCAF11 | 0.512850203 | 1.09E-38 |
| HMGB1 | TP53 | -0.282933103 | 1.03E-11 |
| HMGB1 | TREM2 | 0.084547238 | 0.046100049 |
| HMGB1 | GZMA | -0.091935602 | 0.03004626 |
| HMGB1 | ZBP1 | -0.408061711 | 9.21E-24 |
| IL18 | IL1A | -0.092375842 | 0.029263891 |
| IL18 | IL1B | -0.401510789 | 5.44E-23 |
| IL18 | IL6 | -0.393080451 | 5.05E-22 |
| IL18 | IRF1 | -0.559072122 | 4.10E-47 |
| IL18 | IRF2 | -0.299604015 | 5.14E-13 |
| IL18 | NLRC4 | -0.362835939 | 8.97E-19 |
| IL18 | NLRP1 | 0.359544346 | 1.93E-18 |
| IL18 | NLRP2 | 0.083482425 | 0.048922271 |
| IL18 | NLRP3 | -0.428370852 | 2.90E-26 |
| IL18 | NLRP6 | -0.282641589 | 1.09E-11 |
| IL18 | NLRP7 | -0.525160107 | 8.30E-41 |
| IL18 | NLRP9 | 0.331994182 | 8.48E-16 |

| | | | |
|---|---|---|---|
| IL18 | NOD2 | 0.226606468 | 6.42E-08 |
| IL18 | PLCG1 | -0.269358081 | 1.03E-10 |
| IL18 | PRKACA | -0.354608051 | 6.01E-18 |
| IL18 | PYCARD | 0.099218552 | 0.019172329 |
| IL18 | SCAF11 | 0.504567522 | 2.60E-37 |
| IL18 | TP53 | -0.100501659 | 0.017663111 |
| IL18 | TP63 | 0.187376109 | 8.52E-06 |
| IL18 | GZMA | -0.244297472 | 5.18E-09 |
| IL18 | ZBP1 | -0.643948723 | 1.49E-66 |
| IL1A | IL1B | 0.585125511 | 1.78E-52 |
| IL1A | IL6 | 0.452131348 | 2.05E-29 |
| IL1A | IRF1 | 0.406914933 | 1.26E-23 |
| IL1A | IRF2 | 0.111590566 | 0.008390141 |
| IL1A | NAIP | 0.289950723 | 2.99E-12 |
| IL1A | NLRP1 | -0.149435296 | 0.000402002 |
| IL1A | NLRP3 | 0.282663798 | 1.08E-11 |
| IL1A | NLRP7 | 0.112823233 | 0.007692958 |
| IL1A | NOD2 | -0.149932133 | 0.000384258 |
| IL1A | PLCG1 | -0.207808553 | 7.52E-07 |
| IL1A | PYCARD | -0.396654168 | 1.98E-22 |
| IL1A | TP53 | -0.267530644 | 1.39E-10 |
| IL1A | TP63 | -0.156894413 | 0.000201148 |
| IL1A | GZMA | 0.2464787 | 3.74E-09 |
| IL1A | ZBP1 | 0.24868194 | 2.69E-09 |
| IL1B | IL6 | 0.526955133 | 4.01E-41 |
| IL1B | IRF1 | 0.541961125 | 7.67E-44 |

| | | | |
|---|---|---|---|
| IL1B | IRF2 | 0.152714133 | 0.000297663 |
| IL1B | NAIP | 0.147511103 | 0.00047818 |
| IL1B | NLRC4 | 0.176093448 | 2.92E-05 |
| IL1B | NLRP1 | -0.10534506 | 0.012861826 |
| IL1B | NLRP3 | 0.510905921 | 2.31E-38 |
| IL1B | NLRP6 | 0.229903789 | 4.08E-08 |
| IL1B | NLRP7 | 0.287817245 | 4.37E-12 |
| IL1B | NLRP9 | -0.109548447 | 0.009669782 |
| IL1B | NOD1 | 0.169849627 | 5.60E-05 |
| IL1B | NOD2 | -0.400073174 | 7.99E-23 |
| IL1B | PLCG1 | 0.105864248 | 0.012422785 |
| IL1B | PRKACA | 0.270450491 | 8.59E-11 |
| IL1B | PYCARD | -0.335705549 | 3.87E-16 |
| IL1B | SCAF11 | -0.251762244 | 1.68E-09 |
| IL1B | TP53 | -0.142437851 | 0.000748125 |
| IL1B | TP63 | -0.168028041 | 6.75E-05 |
| IL1B | TREM2 | 0.107075262 | 0.011449783 |
| IL1B | TNF | 0.14463738 | 0.000617256 |
| IL1B | GZMA | 0.191017174 | 5.63E-06 |
| IL1B | ZBP1 | 0.557142942 | 9.80E-47 |
| IL6 | IRF1 | 0.498119106 | 2.89E-36 |
| IL6 | IRF2 | 0.177939841 | 2.40E-05 |
| IL6 | NAIP | 0.144302512 | 0.000635704 |
| IL6 | NLRC4 | 0.121121856 | 0.004200442 |
| IL6 | NLRP1 | -0.193528796 | 4.21E-06 |
| IL6 | NLRP3 | 0.426457192 | 5.07E-26 |

| | | | |
|---|---|---|---|
| IL6 | NLRP6 | 0.120469582 | 0.00441094 |
| IL6 | NLRP7 | 0.240301103 | 9.31E-09 |
| IL6 | NLRP9 | -0.195858189 | 3.21E-06 |
| IL6 | NOD1 | 0.096244117 | 0.023109744 |
| IL6 | PLCG1 | -0.084547189 | 0.046100176 |
| IL6 | PRKACA | 0.099145093 | 0.019262037 |
| IL6 | PYCARD | -0.267366096 | 1.43E-10 |
| IL6 | SCAF11 | -0.232144668 | 2.98E-08 |
| IL6 | TP53 | -0.08709638 | 0.039894776 |
| IL6 | TP63 | -0.096377474 | 0.022919263 |
| IL6 | TNF | 0.084995452 | 0.044953657 |
| IL6 | GZMA | 0.154916344 | 0.000242434 |
| IL6 | ZBP1 | 0.312329158 | 4.55E-14 |
| IRF1 | IRF2 | 0.313641062 | 3.52E-14 |
| IRF1 | NAIP | 0.323339122 | 5.07E-15 |
| IRF1 | NLRC4 | 0.252500898 | 1.50E-09 |
| IRF1 | NLRP1 | -0.1764442 | 2.82E-05 |
| IRF1 | NLRP3 | 0.503735578 | 3.55E-37 |
| IRF1 | NLRP6 | 0.170318109 | 5.34E-05 |
| IRF1 | NLRP7 | 0.408216555 | 8.83E-24 |
| IRF1 | NLRP9 | -0.255445162 | 9.54E-10 |
| IRF1 | NOD2 | -0.248186754 | 2.90E-09 |
| IRF1 | PLCG1 | 0.134867845 | 0.001420599 |
| IRF1 | PRKACA | 0.293748279 | 1.51E-12 |
| IRF1 | PYCARD | -0.17870522 | 2.21E-05 |
| IRF1 | SCAF11 | -0.276279439 | 3.24E-11 |

| | | | |
|---|---|---|---|
| IRF1 | TP63 | -0.309057553 | 8.58E-14 |
| IRF1 | TIRAP | 0.173005311 | 4.05E-05 |
| IRF1 | TNF | 0.159961983 | 0.000149923 |
| IRF1 | GZMA | 0.459812708 | 1.73E-30 |
| IRF1 | ZBP1 | 0.631819667 | 2.12E-63 |
| IRF2 | NAIP | 0.120722897 | 0.004328085 |
| IRF2 | NLRC4 | 0.181310012 | 1.67E-05 |
| IRF2 | NLRP1 | -0.148068857 | 0.00045482 |
| IRF2 | NLRP3 | 0.201012017 | 1.73E-06 |
| IRF2 | NLRP6 | 0.114462952 | 0.006845938 |
| IRF2 | NLRP7 | 0.274090478 | 4.68E-11 |
| IRF2 | NLRP9 | -0.167943905 | 6.80E-05 |
| IRF2 | PRKACA | 0.533293026 | 2.96E-42 |
| IRF2 | PYCARD | -0.149302104 | 0.000406887 |
| IRF2 | SCAF11 | -0.377350893 | 2.73E-20 |
| IRF2 | TP53 | 0.511894544 | 1.58E-38 |
| IRF2 | TP63 | 0.136837146 | 0.001205995 |
| IRF2 | TREM2 | -0.083185619 | 0.049734238 |
| IRF2 | TIRAP | 0.236611635 | 1.58E-08 |
| IRF2 | GZMA | 0.141593484 | 0.000804862 |
| IRF2 | ZBP1 | 0.28121455 | 1.39E-11 |
| NAIP | NLRC4 | 0.126991225 | 0.002677437 |
| NAIP | NLRP1 | -0.133032567 | 0.001651668 |
| NAIP | NLRP3 | 0.095362753 | 0.024403455 |
| NAIP | NLRP6 | -0.137884911 | 0.001104395 |
| NAIP | NLRP7 | 0.178648681 | 2.23E-05 |

| | | | |
|---|---|---|---|
| NAIP | NLRP9 | -0.194939154 | 3.57E-06 |
| NAIP | NOD1 | -0.190734705 | 5.81E-06 |
| NAIP | PLCG1 | -0.274775585 | 4.17E-11 |
| NAIP | SCAF11 | 0.192434534 | 4.78E-06 |
| NAIP | TP53 | -0.127341261 | 0.00260495 |
| NAIP | TP63 | -0.136601008 | 0.001230051 |
| NAIP | TREM2 | -0.165596239 | 8.62E-05 |
| NAIP | TIRAP | 0.175915338 | 2.98E-05 |
| NAIP | GZMA | 0.499353754 | 1.83E-36 |
| NAIP | ZBP1 | 0.199904796 | 1.98E-06 |
| NLRC4 | NLRP1 | -0.482567446 | 7.84E-34 |
| NLRC4 | NLRP3 | 0.173062791 | 4.02E-05 |
| NLRC4 | NLRP7 | 0.474065037 | 1.49E-32 |
| NLRC4 | NLRP9 | -0.383934135 | 5.27E-21 |
| NLRC4 | NOD1 | -0.166486799 | 7.88E-05 |
| NLRC4 | PRKACA | 0.134700415 | 0.001440376 |
| NLRC4 | SCAF11 | -0.22818755 | 5.17E-08 |
| NLRC4 | TP63 | -0.245131751 | 4.57E-09 |
| NLRC4 | TREM2 | -0.321149609 | 7.90E-15 |
| NLRC4 | TIRAP | -0.181195168 | 1.69E-05 |
| NLRC4 | GZMA | 0.263120126 | 2.84E-10 |
| NLRC4 | ZBP1 | 0.332457175 | 7.69E-16 |
| NLRP1 | NLRP3 | -0.148628834 | 0.000432438 |
| NLRP1 | NLRP7 | -0.552378802 | 8.23E-46 |
| NLRP1 | NLRP9 | 0.532618109 | 3.92E-42 |
| NLRP1 | NOD1 | 0.449506449 | 4.69E-29 |

| | | | |
|---|---|---|---|
| NLRP1 | NOD2 | -0.211717609 | 4.59E-07 |
| NLRP1 | PLCG1 | 0.149506503 | 0.000399413 |
| NLRP1 | SCAF11 | 0.199999566 | 1.96E-06 |
| NLRP1 | TP53 | 0.229784414 | 4.15E-08 |
| NLRP1 | TP63 | 0.273979826 | 4.77E-11 |
| NLRP1 | TREM2 | 0.49875688 | 2.28E-36 |
| NLRP1 | TIRAP | 0.352085724 | 1.07E-17 |
| NLRP1 | TNF | 0.14570886 | 0.000561513 |
| NLRP1 | GZMA | -0.299008105 | 5.74E-13 |
| NLRP1 | ZBP1 | -0.327803601 | 2.03E-15 |
| NLRP2 | NLRP6 | -0.10937295 | 0.009787458 |
| NLRP2 | PYCARD | 0.1055047 | 0.012725394 |
| NLRP2 | TREM2 | -0.103403999 | 0.014626898 |
| NLRP2 | TNF | -0.106591648 | 0.01182995 |
| NLRP3 | NLRP6 | 0.209375786 | 6.17E-07 |
| NLRP3 | NLRP7 | 0.313955089 | 3.31E-14 |
| NLRP3 | NLRP9 | -0.149334751 | 0.000405685 |
| NLRP3 | NOD1 | 0.130490446 | 0.002028884 |
| NLRP3 | NOD2 | -0.209902185 | 5.78E-07 |
| NLRP3 | PLCG1 | 0.085309855 | 0.0441639 |
| NLRP3 | PRKACA | 0.273245438 | 5.40E-11 |
| NLRP3 | PYCARD | -0.201258125 | 1.68E-06 |
| NLRP3 | SCAF11 | -0.327848638 | 2.01E-15 |
| NLRP3 | TP63 | -0.22766122 | 5.56E-08 |
| NLRP3 | TNF | 0.135235273 | 0.00137807 |
| NLRP3 | GZMA | 0.227895799 | 5.38E-08 |

| | | | |
|---|---|---|---|
| NLRP3 | ZBP1 | 0.433109664 | 7.13E-27 |
| NLRP6 | NLRP7 | 0.171905247 | 4.53E-05 |
| NLRP6 | NOD1 | 0.114527959 | 0.006814151 |
| NLRP6 | NOD2 | -0.239387304 | 1.06E-08 |
| NLRP6 | PLCG1 | 0.209896391 | 5.78E-07 |
| NLRP6 | PRKACA | 0.234872592 | 2.03E-08 |
| NLRP6 | PYCARD | -0.111602439 | 0.008383165 |
| NLRP6 | SCAF11 | -0.185572748 | 1.04E-05 |
| NLRP6 | TP53 | 0.128297166 | 0.00241602 |
| NLRP6 | TREM2 | 0.161272776 | 0.000132012 |
| NLRP6 | GZMA | -0.08908316 | 0.035562443 |
| NLRP6 | ZBP1 | 0.35614545 | 4.23E-18 |
| NLRP7 | NLRP9 | -0.597059838 | 4.25E-55 |
| NLRP7 | NOD1 | -0.236251986 | 1.67E-08 |
| NLRP7 | PRKACA | 0.228305051 | 5.09E-08 |
| NLRP7 | SCAF11 | -0.266360729 | 1.68E-10 |
| NLRP7 | TP63 | -0.345363636 | 4.77E-17 |
| NLRP7 | TREM2 | -0.385769507 | 3.31E-21 |
| NLRP7 | TIRAP | -0.194922307 | 3.58E-06 |
| NLRP7 | TNF | -0.134271536 | 0.001492195 |
| NLRP7 | GZMA | 0.34891267 | 2.17E-17 |
| NLRP7 | ZBP1 | 0.62724087 | 3.02E-62 |
| NLRP9 | NOD1 | 0.244141077 | 5.30E-09 |
| NLRP9 | NOD2 | -0.112774815 | 0.007719334 |
| NLRP9 | PRKACA | -0.190703482 | 5.83E-06 |
| NLRP9 | PYCARD | -0.141368463 | 0.00082064 |

| | | | |
|---|---|---|---|
| NLRP9 | SCAF11 | 0.093249584 | 0.027762796 |
| NLRP9 | TP63 | 0.192679998 | 4.65E-06 |
| NLRP9 | TREM2 | 0.498023121 | 2.99E-36 |
| NLRP9 | TIRAP | 0.292383922 | 1.93E-12 |
| NLRP9 | TNF | 0.086134796 | 0.042147053 |
| NLRP9 | GZMA | -0.31969649 | 1.06E-14 |
| NLRP9 | ZBP1 | -0.418735904 | 4.68E-25 |
| NOD1 | NOD2 | -0.314869555 | 2.76E-14 |
| NOD1 | PLCG1 | 0.365098453 | 5.26E-19 |
| NOD1 | PRKACA | 0.148478003 | 0.000438364 |
| NOD1 | PYCARD | -0.144033264 | 0.000650906 |
| NOD1 | SCAF11 | -0.181824989 | 1.58E-05 |
| NOD1 | TP63 | 0.159953601 | 0.000150045 |
| NOD1 | TREM2 | 0.328425957 | 1.78E-15 |
| NOD1 | TIRAP | 0.116365752 | 0.005968499 |
| NOD1 | TNF | 0.110111554 | 0.009300649 |
| NOD2 | PLCG1 | -0.274640004 | 4.27E-11 |
| NOD2 | PRKACA | -0.146356464 | 0.000530128 |
| NOD2 | PYCARD | 0.344443366 | 5.84E-17 |
| NOD2 | TP63 | 0.09094271 | 0.031876823 |
| NOD2 | TREM2 | -0.350984044 | 1.37E-17 |
| NOD2 | TIRAP | -0.244230134 | 5.23E-09 |
| NOD2 | GZMA | -0.100631507 | 0.017516328 |
| NOD2 | ZBP1 | -0.385493453 | 3.55E-21 |
| PLCG1 | PRKACA | 0.238252426 | 1.25E-08 |
| PLCG1 | SCAF11 | -0.222988188 | 1.05E-07 |

| | | | |
|---|---|---|---|
| PLCG1 | TP53 | 0.114311758 | 0.006920385 |
| PLCG1 | TREM2 | 0.237079998 | 1.48E-08 |
| PLCG1 | TIRAP | -0.104920805 | 0.013230698 |
| PLCG1 | GZMA | -0.091219956 | 0.03135632 |
| PLCG1 | ZBP1 | 0.229399569 | 4.37E-08 |
| PRKACA | PYCARD | 0.103782596 | 0.014266786 |
| PRKACA | SCAF11 | -0.484539049 | 3.92E-34 |
| PRKACA | TP53 | 0.502637865 | 5.37E-37 |
| PRKACA | TIRAP | 0.120245548 | 0.00448541 |
| PRKACA | TNF | 0.179347986 | 2.06E-05 |
| PRKACA | GZMA | -0.088449437 | 0.036898648 |
| PRKACA | ZBP1 | 0.417702938 | 6.28E-25 |
| PYCARD | TP53 | 0.301229307 | 3.80E-13 |
| PYCARD | TREM2 | -0.134155422 | 0.001506517 |
| PYCARD | TIRAP | -0.152164718 | 0.000313167 |
| PYCARD | GZMA | -0.153089694 | 0.000287481 |
| PYCARD | ZBP1 | -0.232425109 | 2.87E-08 |
| SCAF11 | TP53 | -0.243107615 | 6.17E-09 |
| SCAF11 | TP63 | 0.100517993 | 0.017644588 |
| SCAF11 | TIRAP | -0.093947872 | 0.026611199 |
| SCAF11 | TNF | -0.105171292 | 0.013011798 |
| SCAF11 | ZBP1 | -0.298791827 | 5.98E-13 |
| TP53 | TP63 | 0.468226373 | 1.07E-31 |
| TP53 | TREM2 | 0.093712399 | 0.02699485 |
| TP53 | TIRAP | 0.246806186 | 3.56E-09 |
| TP53 | GZMA | -0.23921111 | 1.09E-08 |

| | | | |
|---|---|---|---|
| TP63 | TREM2 | 0.170546067 | 5.22E-05 |
| TP63 | TIRAP | 0.102247205 | 0.015777194 |
| TP63 | TNF | -0.142204397 | 0.00076343 |
| TP63 | GZMA | -0.24353364 | 5.79E-09 |
| TP63 | ZBP1 | -0.305978756 | 1.55E-13 |
| TREM2 | TIRAP | 0.481901477 | 9.91E-34 |
| TREM2 | GZMA | -0.225972287 | 7.01E-08 |
| TIRAP | ZBP1 | 0.114634973 | 0.006762111 |
| GZMA | ZBP1 | 0.369787358 | 1.72E-19 |

**Table S4. Correlations between pyroptosis-related genes in periodontitis samples**

| Gene1 | Gene2 | cor | pvalue |
|---|---|---|---|
| AIM2 | APIP | -0.111790206 | 0.021316759 |
| AIM2 | CASP1 | 0.510703998 | 1.53E-29 |
| AIM2 | CASP3 | 0.563082257 | 7.78E-37 |
| AIM2 | CASP4 | 0.224545789 | 3.01E-06 |
| AIM2 | CASP5 | 0.188692695 | 9.26E-05 |
| AIM2 | CASP8 | 0.311012407 | 5.82E-11 |
| AIM2 | CHMP2B | -0.130526297 | 0.007117645 |
| AIM2 | CHMP4B | -0.194132886 | 5.72E-05 |
| AIM2 | CHMP4C | -0.255168319 | 9.98E-08 |
| AIM2 | CHMP6 | -0.322926233 | 9.54E-12 |
| AIM2 | CHMP7 | 0.141671174 | 0.003463046 |
| AIM2 | CYCS | -0.111578065 | 0.02156577 |
| AIM2 | DHX9 | -0.130536145 | 0.007113276 |
| AIM2 | ELANE | -0.109211494 | 0.024519235 |

| | | | |
|---|---|---|---|
| AIM2 | GSDMB | 0.212460114 | 1.02E-05 |
| AIM2 | GSDMC | -0.291117153 | 9.99E-10 |
| AIM2 | GSDMD | 0.182745407 | 0.000154477 |
| AIM2 | GZMB | 0.418910919 | 1.91E-19 |
| AIM2 | HMGB1 | 0.165260101 | 0.000634579 |
| AIM2 | IL18 | -0.210883228 | 1.19E-05 |
| AIM2 | IL1A | 0.388848176 | 9.36E-17 |
| AIM2 | IL1B | 0.435807666 | 4.40E-21 |
| AIM2 | IL6 | 0.227845989 | 2.13E-06 |
| AIM2 | IRF1 | 0.454288955 | 5.58E-23 |
| AIM2 | IRF2 | -0.098496492 | 0.042651062 |
| AIM2 | NAIP | 0.268502303 | 1.96E-08 |
| AIM2 | NLRC4 | 0.131313997 | 0.006775689 |
| AIM2 | NLRP1 | -0.181127513 | 0.000177051 |
| AIM2 | NLRP3 | 0.285589438 | 2.12E-09 |
| AIM2 | NLRP6 | 0.131883041 | 0.006537887 |
| AIM2 | NLRP7 | 0.204619677 | 2.18E-05 |
| AIM2 | NOD1 | -0.097887898 | 0.043954431 |
| AIM2 | NOD2 | -0.217851488 | 5.98E-06 |
| AIM2 | PRKACA | -0.171835787 | 0.000378925 |
| AIM2 | PYCARD | -0.271335722 | 1.37E-08 |
| AIM2 | SCAF11 | 0.127512568 | 0.00857292 |
| AIM2 | TP53 | -0.453690379 | 6.45E-23 |
| AIM2 | TP63 | -0.383359353 | 2.71E-16 |
| AIM2 | GZMA | 0.400368606 | 9.38E-18 |
| AIM2 | ZBP1 | 0.379094459 | 6.12E-16 |

| | | | |
|---|---|---|---|
| APIP | BAK1 | -0.33465005 | 1.49E-12 |
| APIP | BAX | 0.131795733 | 0.00657388 |
| APIP | CASP1 | 0.131661395 | 0.006629608 |
| APIP | CASP3 | 0.206535235 | 1.81E-05 |
| APIP | CASP4 | 0.384340131 | 2.25E-16 |
| APIP | CASP5 | 0.109708176 | 0.023871951 |
| APIP | CHMP2A | 0.115761245 | 0.017095141 |
| APIP | CHMP2B | 0.402251424 | 6.38E-18 |
| APIP | CHMP4B | 0.123547269 | 0.01088885 |
| APIP | CHMP4C | 0.336832787 | 1.04E-12 |
| APIP | CHMP6 | -0.155322161 | 0.001335085 |
| APIP | CYCS | 0.194446821 | 5.56E-05 |
| APIP | ELANE | 0.152039235 | 0.001691157 |
| APIP | GPX4 | -0.373000296 | 1.92E-15 |
| APIP | GSDMB | 0.218562347 | 5.57E-06 |
| APIP | GSDMD | -0.206349892 | 1.85E-05 |
| APIP | GZMB | -0.229531685 | 1.79E-06 |
| APIP | HMGB1 | 0.121652773 | 0.0121793 |
| APIP | IL18 | 0.148133627 | 0.00222714 |
| APIP | IL1B | -0.277204247 | 6.43E-09 |
| APIP | IRF1 | -0.122307833 | 0.011718587 |
| APIP | IRF2 | 0.23007374 | 1.69E-06 |
| APIP | NAIP | 0.217169645 | 6.40E-06 |
| APIP | NLRC4 | 0.186332375 | 0.000113692 |
| APIP | NLRP1 | -0.443137664 | 8.04E-22 |
| APIP | NLRP3 | -0.155694269 | 0.001299408 |

| | | | |
|---|---|---|---|
| APIP | NLRP6 | -0.286488154 | 1.88E-09 |
| APIP | NLRP7 | 0.162338421 | 0.000793172 |
| APIP | NLRP9 | -0.294210987 | 6.51E-10 |
| APIP | NOD1 | -0.495390985 | 1.22E-27 |
| APIP | NOD2 | 0.445758685 | 4.33E-22 |
| APIP | PLCG1 | -0.450291582 | 1.47E-22 |
| APIP | PRKACA | -0.171288766 | 0.000395817 |
| APIP | TREM2 | -0.464789049 | 4.13E-24 |
| APIP | TNF | -0.191372031 | 7.32E-05 |
| APIP | GZMA | 0.278476722 | 5.44E-09 |
| APIP | ZBP1 | -0.177370669 | 0.000241936 |
| BAK1 | BAX | 0.260581815 | 5.20E-08 |
| BAK1 | CASP1 | -0.273294733 | 1.06E-08 |
| BAK1 | CASP3 | -0.10874128 | 0.025145933 |
| BAK1 | CASP4 | -0.275532364 | 7.98E-09 |
| BAK1 | CASP5 | -0.155707946 | 0.001298113 |
| BAK1 | CASP6 | -0.396898208 | 1.89E-17 |
| BAK1 | CASP8 | -0.226185557 | 2.54E-06 |
| BAK1 | CHMP2B | -0.515724396 | 3.46E-30 |
| BAK1 | CHMP4B | 0.111741033 | 0.021374255 |
| BAK1 | CHMP4C | -0.385500948 | 1.80E-16 |
| BAK1 | CHMP6 | 0.471184588 | 8.08E-25 |
| BAK1 | CHMP7 | 0.126022261 | 0.009386123 |
| BAK1 | ELANE | 0.254842393 | 1.04E-07 |
| BAK1 | GPX4 | 0.360804696 | 1.76E-14 |
| BAK1 | GSDMB | 0.095769649 | 0.048754998 |

| | | | |
|---|---|---|---|
| BAK1 | GSDMC | -0.173960031 | 0.000319493 |
| BAK1 | GSDMD | 0.140195809 | 0.003820861 |
| BAK1 | GZMB | 0.096010573 | 0.048187784 |
| BAK1 | HMGB1 | -0.46749704 | 2.08E-24 |
| BAK1 | IL18 | -0.223387233 | 3.40E-06 |
| BAK1 | IL1A | -0.115345512 | 0.017499881 |
| BAK1 | IL1B | 0.106131396 | 0.028881336 |
| BAK1 | IRF1 | 0.187894861 | 9.93E-05 |
| BAK1 | IRF2 | 0.216046093 | 7.17E-06 |
| BAK1 | NAIP | -0.202462406 | 2.67E-05 |
| BAK1 | NLRP3 | 0.164413058 | 0.000677233 |
| BAK1 | NLRP6 | 0.227139214 | 2.30E-06 |
| BAK1 | NOD1 | 0.099398862 | 0.040778576 |
| BAK1 | PLCG1 | 0.280844116 | 3.99E-09 |
| BAK1 | PRKACA | 0.481792044 | 5.00E-26 |
| BAK1 | PYCARD | 0.318760033 | 1.81E-11 |
| BAK1 | SCAF11 | -0.299749667 | 2.99E-10 |
| BAK1 | TP53 | 0.414721557 | 4.69E-19 |
| BAK1 | TP63 | 0.098169131 | 0.043348035 |
| BAK1 | TREM2 | 0.194723073 | 5.43E-05 |
| BAK1 | TIRAP | 0.101748996 | 0.036224966 |
| BAK1 | GZMA | -0.240717268 | 5.28E-07 |
| BAK1 | ZBP1 | 0.284780874 | 2.36E-09 |
| BAX | CASP4 | 0.213648447 | 9.09E-06 |
| BAX | CASP5 | -0.096823497 | 0.046314499 |
| BAX | CASP6 | -0.499540384 | 3.81E-28 |

| | | | |
|---|---|---|---|
| BAX | CASP8 | -0.432059155 | 1.03E-20 |
| BAX | CASP9 | 0.190381704 | 7.99E-05 |
| BAX | CHMP2A | 0.398547973 | 1.36E-17 |
| BAX | CHMP2B | -0.382970074 | 2.93E-16 |
| BAX | CHMP4B | 0.719047132 | 1.09E-68 |
| BAX | CHMP4C | -0.256267473 | 8.75E-08 |
| BAX | CHMP6 | 0.40941513 | 1.44E-18 |
| BAX | CHMP7 | 0.161907657 | 0.000819438 |
| BAX | CYCS | -0.111543146 | 0.021606998 |
| BAX | DHX9 | 0.213419586 | 9.30E-06 |
| BAX | ELANE | 0.581209124 | 1.12E-39 |
| BAX | GPX4 | 0.218109719 | 5.83E-06 |
| BAX | GSDMB | 0.341581352 | 4.78E-13 |
| BAX | GSDMD | 0.334409471 | 1.55E-12 |
| BAX | HMGB1 | -0.507233258 | 4.21E-29 |
| BAX | IL18 | -0.45134235 | 1.14E-22 |
| BAX | IL1B | 0.121793975 | 0.01207866 |
| BAX | IL6 | 0.130847596 | 0.006976341 |
| BAX | IRF1 | 0.220569092 | 4.54E-06 |
| BAX | NAIP | 0.11680478 | 0.016115054 |
| BAX | NLRC4 | 0.373414141 | 1.78E-15 |
| BAX | NLRP1 | -0.454411993 | 5.41E-23 |
| BAX | NLRP6 | 0.173673386 | 0.000326971 |
| BAX | NLRP7 | 0.507336416 | 4.09E-29 |
| BAX | NLRP9 | -0.631256171 | 1.62E-48 |
| BAX | NOD1 | -0.267856713 | 2.12E-08 |

| | | | |
|---|---|---|---|
| BAX | NOD2 | 0.216853525 | 6.61E-06 |
| BAX | PLCG1 | 0.166499187 | 0.000576652 |
| BAX | PRKACA | 0.24381615 | 3.72E-07 |
| BAX | PYCARD | 0.418073427 | 2.28E-19 |
| BAX | SCAF11 | -0.201379755 | 2.95E-05 |
| BAX | TP53 | 0.257104937 | 7.92E-08 |
| BAX | TREM2 | -0.349548826 | 1.25E-13 |
| BAX | TIRAP | -0.237820307 | 7.28E-07 |
| BAX | TNF | -0.210423357 | 1.25E-05 |
| BAX | GZMA | 0.148589295 | 0.002157458 |
| BAX | ZBP1 | 0.424267315 | 5.91E-20 |
| CASP1 | CASP3 | 0.380307077 | 4.87E-16 |
| CASP1 | CASP4 | 0.378180136 | 7.28E-16 |
| CASP1 | CASP5 | 0.227559862 | 2.20E-06 |
| CASP1 | CASP6 | 0.2339878 | 1.11E-06 |
| CASP1 | CASP8 | 0.415044038 | 4.38E-19 |
| CASP1 | CASP9 | 0.133274643 | 0.005987552 |
| CASP1 | CHMP2B | 0.145106609 | 0.002744684 |
| CASP1 | CHMP6 | -0.405740984 | 3.11E-18 |
| CASP1 | CHMP7 | -0.19508826 | 5.25E-05 |
| CASP1 | CYCS | -0.10260949 | 0.034669363 |
| CASP1 | DHX9 | 0.166967168 | 0.00055608 |
| CASP1 | ELANE | -0.190375514 | 7.99E-05 |
| CASP1 | GPX4 | -0.240991796 | 5.12E-07 |
| CASP1 | GSDMB | 0.099252077 | 0.041078351 |
| CASP1 | GSDMC | 0.169290541 | 0.000463662 |

| | | | |
|---|---|---|---|
| CASP1 | GSDMD | -0.099165471 | 0.041256096 |
| CASP1 | GZMB | 0.201274049 | 2.98E-05 |
| CASP1 | HMGB1 | 0.338166575 | 8.39E-13 |
| CASP1 | IL18 | 0.206672578 | 1.79E-05 |
| CASP1 | IL1A | 0.293425719 | 7.26E-10 |
| CASP1 | IL1B | 0.269163761 | 1.80E-08 |
| CASP1 | IRF1 | 0.158287688 | 0.001074101 |
| CASP1 | IRF2 | -0.209932177 | 1.31E-05 |
| CASP1 | NAIP | 0.306586443 | 1.12E-10 |
| CASP1 | NLRP1 | -0.144562453 | 0.002848577 |
| CASP1 | NLRP2 | 0.158382059 | 0.001066626 |
| CASP1 | NOD2 | -0.104378608 | 0.031649085 |
| CASP1 | PLCG1 | -0.195103573 | 5.24E-05 |
| CASP1 | PRKACA | -0.375649271 | 1.17E-15 |
| CASP1 | PYCARD | -0.232401954 | 1.31E-06 |
| CASP1 | SCAF11 | 0.262984436 | 3.88E-08 |
| CASP1 | TP53 | -0.393479023 | 3.75E-17 |
| CASP1 | GZMA | 0.443258009 | 7.81E-22 |
| CASP3 | CASP4 | 0.226170532 | 2.55E-06 |
| CASP3 | CASP5 | 0.215980604 | 7.21E-06 |
| CASP3 | CASP6 | -0.127039371 | 0.00882405 |
| CASP3 | CASP8 | 0.319322285 | 1.66E-11 |
| CASP3 | CASP9 | 0.111341309 | 0.02184665 |
| CASP3 | CHMP2B | -0.112197436 | 0.020845738 |
| CASP3 | CHMP4C | -0.20588457 | 1.93E-05 |
| CASP3 | CHMP6 | -0.291832532 | 9.05E-10 |

| | | | |
|---|---|---|---|
| CASP3 | GPX4 | -0.296745209 | 4.57E-10 |
| CASP3 | GSDMB | 0.324486235 | 7.49E-12 |
| CASP3 | GSDMC | -0.212362753 | 1.03E-05 |
| CASP3 | GZMB | 0.150642666 | 0.001867495 |
| CASP3 | HMGB1 | 0.126264025 | 0.009249712 |
| CASP3 | IL18 | -0.330984677 | 2.68E-12 |
| CASP3 | IL1A | 0.283017828 | 2.99E-09 |
| CASP3 | IL1B | 0.299493028 | 3.10E-10 |
| CASP3 | IL6 | 0.255053112 | 1.01E-07 |
| CASP3 | IRF1 | 0.334593979 | 1.50E-12 |
| CASP3 | NAIP | 0.238305825 | 6.90E-07 |
| CASP3 | NLRC4 | 0.258224228 | 6.92E-08 |
| CASP3 | NLRP1 | -0.486274399 | 1.50E-26 |
| CASP3 | NLRP3 | 0.263457262 | 3.66E-08 |
| CASP3 | NLRP6 | 0.102074076 | 0.035630455 |
| CASP3 | NLRP7 | 0.34833926 | 1.53E-13 |
| CASP3 | NLRP9 | -0.129876398 | 0.007411316 |
| CASP3 | NOD1 | -0.352634994 | 7.35E-14 |
| CASP3 | PLCG1 | -0.231913932 | 1.38E-06 |
| CASP3 | PRKACA | -0.199897411 | 3.38E-05 |
| CASP3 | PYCARD | -0.341241868 | 5.05E-13 |
| CASP3 | TP53 | -0.393820645 | 3.51E-17 |
| CASP3 | TP63 | -0.33407778 | 1.63E-12 |
| CASP3 | TNF | -0.098620187 | 0.042390178 |
| CASP3 | GZMA | 0.299320297 | 3.18E-10 |
| CASP3 | ZBP1 | 0.481345305 | 5.64E-26 |

| | | | |
|---|---|---|---|
| CASP4 | CASP5 | 0.161488184 | 0.000845786 |
| CASP4 | CASP8 | -0.140713348 | 0.003691701 |
| CASP4 | CASP9 | 0.141609876 | 0.003477285 |
| CASP4 | CHMP2A | 0.343432562 | 3.51E-13 |
| CASP4 | CHMP2B | 0.272758405 | 1.14E-08 |
| CASP4 | CHMP4B | 0.151038368 | 0.001815896 |
| CASP4 | CHMP4C | 0.150854979 | 0.001839645 |
| CASP4 | CHMP6 | -0.147474693 | 0.002331546 |
| CASP4 | CHMP7 | 0.145896798 | 0.002599952 |
| CASP4 | ELANE | 0.109716646 | 0.023861041 |
| CASP4 | GPX4 | -0.239017713 | 6.37E-07 |
| CASP4 | GSDMB | 0.169488786 | 0.000456477 |
| CASP4 | GSDMC | 0.109192881 | 0.024543784 |
| CASP4 | IL1A | 0.165800691 | 0.000608673 |
| CASP4 | IL1B | 0.107992813 | 0.026171985 |
| CASP4 | IL6 | 0.124208015 | 0.010468133 |
| CASP4 | NAIP | 0.240088644 | 5.66E-07 |
| CASP4 | NLRC4 | 0.284360648 | 2.50E-09 |
| CASP4 | NLRP1 | -0.431385132 | 1.21E-20 |
| CASP4 | NLRP3 | -0.109327159 | 0.02436716 |
| CASP4 | NLRP7 | 0.208872594 | 1.45E-05 |
| CASP4 | NLRP9 | -0.309820246 | 6.94E-11 |
| CASP4 | NOD1 | -0.287608848 | 1.61E-09 |
| CASP4 | NOD2 | 0.148267025 | 0.002206531 |
| CASP4 | PLCG1 | -0.305080396 | 1.39E-10 |
| CASP4 | PRKACA | -0.175562998 | 0.000280531 |

| | | | |
|---|---|---|---|
| CASP4 | SCAF11 | 0.15748233 | 0.001139884 |
| CASP4 | TP53 | -0.276550685 | 7.00E-09 |
| CASP4 | TP63 | -0.101491888 | 0.036701117 |
| CASP4 | TREM2 | -0.527774995 | 8.83E-32 |
| CASP4 | TIRAP | -0.349748163 | 1.21E-13 |
| CASP4 | TNF | -0.100510478 | 0.038567772 |
| CASP4 | GZMA | 0.245685916 | 3.01E-07 |
| CASP5 | CASP6 | 0.152430478 | 0.001644569 |
| CASP5 | CASP8 | 0.145808547 | 0.002615764 |
| CASP5 | CHMP2B | 0.135871866 | 0.005070492 |
| CASP5 | CHMP4B | -0.165278624 | 0.000633675 |
| CASP5 | CHMP4C | -0.145421472 | 0.002686157 |
| CASP5 | CHMP6 | -0.175602972 | 0.000279619 |
| CASP5 | GPX4 | -0.196067203 | 4.81E-05 |
| CASP5 | GSDMB | 0.124983563 | 0.009992753 |
| CASP5 | GSDMD | 0.117774797 | 0.015248513 |
| CASP5 | GZMB | 0.153027207 | 0.001575774 |
| CASP5 | HMGB1 | 0.121800988 | 0.012073681 |
| CASP5 | IL1A | 0.225535638 | 2.72E-06 |
| CASP5 | IL1B | 0.19718734 | 4.34E-05 |
| CASP5 | NAIP | 0.205824832 | 1.94E-05 |
| CASP5 | NLRC4 | 0.126075389 | 0.009355994 |
| CASP5 | PLCG1 | -0.2325864 | 1.29E-06 |
| CASP5 | PYCARD | -0.109971388 | 0.023534953 |
| CASP5 | TP53 | -0.264484646 | 3.23E-08 |
| CASP5 | TP63 | -0.202965309 | 2.54E-05 |

| | | | |
|---|---|---|---|
| CASP5 | TIRAP | 0.128594373 | 0.008022562 |
| CASP5 | GZMA | 0.14935394 | 0.002044987 |
| CASP6 | CASP8 | 0.306643326 | 1.11E-10 |
| CASP6 | CHMP2B | 0.408265964 | 1.84E-18 |
| CASP6 | CHMP4B | -0.408168794 | 1.88E-18 |
| CASP6 | CHMP4C | 0.350144328 | 1.13E-13 |
| CASP6 | CHMP6 | -0.371741031 | 2.42E-15 |
| CASP6 | CHMP7 | -0.308802903 | 8.06E-11 |
| CASP6 | CYCS | 0.187311628 | 0.00010446 |
| CASP6 | ELANE | -0.427075261 | 3.17E-20 |
| CASP6 | GPX4 | -0.230217687 | 1.66E-06 |
| CASP6 | GSDMB | -0.097814363 | 0.04411416 |
| CASP6 | GSDMC | 0.271743654 | 1.30E-08 |
| CASP6 | GSDMD | -0.263060959 | 3.84E-08 |
| CASP6 | HMGB1 | 0.591288088 | 2.47E-41 |
| CASP6 | IL18 | 0.596171449 | 3.71E-42 |
| CASP6 | IL1B | -0.196989601 | 4.42E-05 |
| CASP6 | IL6 | -0.253594396 | 1.20E-07 |
| CASP6 | IRF1 | -0.381915336 | 3.58E-16 |
| CASP6 | IRF2 | -0.292944055 | 7.76E-10 |
| CASP6 | NLRC4 | -0.277681877 | 6.04E-09 |
| CASP6 | NLRP1 | 0.229865662 | 1.72E-06 |
| CASP6 | NLRP3 | -0.228418896 | 2.01E-06 |
| CASP6 | NLRP6 | -0.253444594 | 1.22E-07 |
| CASP6 | NLRP7 | -0.351625166 | 8.75E-14 |
| CASP6 | NLRP9 | 0.342691259 | 3.97E-13 |

| | | | |
|---|---|---|---|
| CASP6 | NOD2 | -0.144104796 | 0.002938711 |
| CASP6 | PLCG1 | -0.206206474 | 1.87E-05 |
| CASP6 | PRKACA | -0.446478053 | 3.65E-22 |
| CASP6 | PYCARD | -0.134820794 | 0.005425179 |
| CASP6 | SCAF11 | 0.509774025 | 2.01E-29 |
| CASP6 | TP53 | -0.343493335 | 3.47E-13 |
| CASP6 | TREM2 | 0.102262951 | 0.035288862 |
| CASP6 | ZBP1 | -0.456565191 | 3.19E-23 |
| CASP8 | CHMP2A | -0.363117889 | 1.16E-14 |
| CASP8 | CHMP2B | 0.151536987 | 0.001752736 |
| CASP8 | CHMP4B | -0.344701198 | 2.84E-13 |
| CASP8 | CHMP6 | -0.393909309 | 3.44E-17 |
| CASP8 | CHMP7 | -0.339547575 | 6.69E-13 |
| CASP8 | CYCS | 0.144317974 | 0.002896409 |
| CASP8 | DHX9 | 0.150955918 | 0.001826538 |
| CASP8 | ELANE | -0.354226973 | 5.58E-14 |
| CASP8 | GPX4 | -0.364573314 | 8.95E-15 |
| CASP8 | GSDMB | -0.12707092 | 0.008807105 |
| CASP8 | GSDMD | -0.402252231 | 6.38E-18 |
| CASP8 | GZMB | 0.154722169 | 0.001394514 |
| CASP8 | HMGB1 | 0.545554926 | 2.99E-34 |
| CASP8 | IL18 | 0.189237988 | 8.83E-05 |
| CASP8 | IL1A | 0.162038336 | 0.000811386 |
| CASP8 | IL1B | 0.102318656 | 0.035188648 |
| CASP8 | IRF2 | -0.156614949 | 0.001214868 |
| CASP8 | NAIP | 0.135812525 | 0.005089947 |

| | | | |
|---|---|---|---|
| CASP8 | NLRC4 | -0.265107324 | 2.99E-08 |
| CASP8 | NLRP1 | 0.204049457 | 2.30E-05 |
| CASP8 | NLRP7 | -0.275121231 | 8.42E-09 |
| CASP8 | NLRP9 | 0.447166046 | 3.10E-22 |
| CASP8 | NOD1 | 0.145116481 | 0.002742832 |
| CASP8 | NOD2 | -0.196977545 | 4.42E-05 |
| CASP8 | PLCG1 | -0.230959824 | 1.53E-06 |
| CASP8 | PRKACA | -0.406931092 | 2.43E-18 |
| CASP8 | PYCARD | -0.447377351 | 2.95E-22 |
| CASP8 | SCAF11 | 0.218338073 | 5.69E-06 |
| CASP8 | TP53 | -0.291919282 | 8.94E-10 |
| CASP8 | TP63 | 0.11181107 | 0.021292404 |
| CASP8 | TREM2 | 0.340626411 | 5.60E-13 |
| CASP8 | TIRAP | 0.348411742 | 1.52E-13 |
| CASP8 | GZMA | 0.216292721 | 6.99E-06 |
| CASP9 | CHMP2A | 0.229833183 | 1.73E-06 |
| CASP9 | CHMP4B | 0.159710449 | 0.000966372 |
| CASP9 | CHMP6 | 0.170566225 | 0.000419204 |
| CASP9 | CHMP7 | 0.142018182 | 0.00338343 |
| CASP9 | DHX9 | 0.11089335 | 0.022386781 |
| CASP9 | GPX4 | 0.099968324 | 0.039633001 |
| CASP9 | GZMB | 0.110768583 | 0.022539265 |
| CASP9 | HMGB1 | -0.158667013 | 0.001044346 |
| CASP9 | IL1B | 0.154482738 | 0.0014189 |
| CASP9 | NAIP | -0.213005539 | 9.69E-06 |
| CASP9 | NLRC4 | 0.148472901 | 0.002175065 |

| | | | |
|---|---|---|---|
| CASP9 | NLRP1 | -0.148308059 | 0.002200226 |
| CASP9 | NLRP6 | 0.158874073 | 0.001028426 |
| CASP9 | NLRP7 | 0.105447316 | 0.029935797 |
| CASP9 | SCAF11 | -0.136310606 | 0.004928722 |
| CASP9 | TREM2 | -0.114551348 | 0.018296364 |
| CASP9 | TIRAP | -0.13661216 | 0.004833363 |
| CASP9 | GZMA | -0.099636506 | 0.040297156 |
| CHMP2A | CHMP4B | 0.24964878 | 1.91E-07 |
| CHMP2A | CHMP6 | 0.129051592 | 0.007799592 |
| CHMP2A | CHMP7 | 0.171905161 | 0.000376832 |
| CHMP2A | CYCS | -0.130647592 | 0.007064003 |
| CHMP2A | ELANE | 0.208136679 | 1.56E-05 |
| CHMP2A | GPX4 | 0.145866117 | 0.002605439 |
| CHMP2A | GSDMB | 0.100440481 | 0.038703925 |
| CHMP2A | GSDMD | 0.250842769 | 1.66E-07 |
| CHMP2A | HMGB1 | -0.200707792 | 3.14E-05 |
| CHMP2A | IL1A | -0.144300574 | 0.00289984 |
| CHMP2A | IL6 | -0.108576902 | 0.025368252 |
| CHMP2A | IRF1 | -0.165722643 | 0.000612352 |
| CHMP2A | IRF2 | -0.352637376 | 7.35E-14 |
| CHMP2A | NAIP | -0.101412816 | 0.036848618 |
| CHMP2A | NLRC4 | 0.201512498 | 2.91E-05 |
| CHMP2A | NLRP1 | -0.345967429 | 2.29E-13 |
| CHMP2A | NLRP7 | 0.315505948 | 2.97E-11 |
| CHMP2A | NLRP9 | -0.382335163 | 3.30E-16 |
| CHMP2A | NOD1 | -0.223663533 | 3.30E-06 |

| | | | |
|---|---|---|---|
| CHMP2A | PLCG1 | 0.100823134 | 0.037964557 |
| CHMP2A | PRKACA | -0.138738015 | 0.004206968 |
| CHMP2A | PYCARD | 0.317988809 | 2.04E-11 |
| CHMP2A | TP53 | -0.193554673 | 6.03E-05 |
| CHMP2A | TP63 | -0.1573427 | 0.001151659 |
| CHMP2A | TREM2 | -0.360819239 | 1.75E-14 |
| CHMP2A | TIRAP | -0.486988329 | 1.24E-26 |
| CHMP2A | TNF | -0.197183579 | 4.34E-05 |
| CHMP2B | CHMP4B | -0.179752031 | 0.000198637 |
| CHMP2B | CHMP4C | 0.557556252 | 5.27E-36 |
| CHMP2B | CHMP6 | -0.31906476 | 1.73E-11 |
| CHMP2B | CHMP7 | -0.137797539 | 0.004474427 |
| CHMP2B | CYCS | 0.285379449 | 2.18E-09 |
| CHMP2B | ELANE | -0.352509297 | 7.51E-14 |
| CHMP2B | GPX4 | -0.374909889 | 1.35E-15 |
| CHMP2B | GSDMB | -0.26117447 | 4.84E-08 |
| CHMP2B | GSDMC | 0.302774121 | 1.94E-10 |
| CHMP2B | GSDMD | -0.424390955 | 5.75E-20 |
| CHMP2B | GZMB | -0.177478074 | 0.000239806 |
| CHMP2B | HMGB1 | 0.45673918 | 3.06E-23 |
| CHMP2B | IL18 | 0.519029349 | 1.28E-30 |
| CHMP2B | IL1A | 0.131140384 | 0.006849768 |
| CHMP2B | IL1B | -0.263405928 | 3.68E-08 |
| CHMP2B | IRF1 | -0.357270418 | 3.28E-14 |
| CHMP2B | NLRC4 | -0.098008524 | 0.043693468 |
| CHMP2B | NLRP3 | -0.272303263 | 1.21E-08 |

| | | | |
|---|---|---|---|
| CHMP2B | NLRP6 | -0.278604835 | 5.35E-09 |
| CHMP2B | NLRP7 | -0.30627261 | 1.17E-10 |
| CHMP2B | NLRP9 | 0.141059477 | 0.003607539 |
| CHMP2B | NOD2 | 0.211898008 | 1.08E-05 |
| CHMP2B | PLCG1 | -0.403698834 | 4.74E-18 |
| CHMP2B | PRKACA | -0.387300574 | 1.27E-16 |
| CHMP2B | PYCARD | -0.218174349 | 5.79E-06 |
| CHMP2B | SCAF11 | 0.355026636 | 4.86E-14 |
| CHMP2B | TP53 | -0.244505396 | 3.44E-07 |
| CHMP2B | TREM2 | -0.284379329 | 2.49E-09 |
| CHMP2B | TIRAP | -0.172532029 | 0.000358397 |
| CHMP2B | ZBP1 | -0.533847925 | 1.31E-32 |
| CHMP4B | CHMP4C | -0.112643099 | 0.020340648 |
| CHMP4B | CHMP6 | 0.308907293 | 7.94E-11 |
| CHMP4B | DHX9 | 0.316432937 | 2.58E-11 |
| CHMP4B | ELANE | 0.474477512 | 3.44E-25 |
| CHMP4B | GSDMB | 0.170414017 | 0.000424292 |
| CHMP4B | GSDMC | 0.145741631 | 0.002627812 |
| CHMP4B | GSDMD | 0.120108913 | 0.013328915 |
| CHMP4B | GZMB | -0.148164018 | 0.00222243 |
| CHMP4B | HMGB1 | -0.378104111 | 7.39E-16 |
| CHMP4B | IL18 | -0.289292798 | 1.28E-09 |
| CHMP4B | NLRC4 | 0.171204738 | 0.000398473 |
| CHMP4B | NLRP1 | -0.286861761 | 1.78E-09 |
| CHMP4B | NLRP7 | 0.232533586 | 1.29E-06 |
| CHMP4B | NLRP9 | -0.399153105 | 1.20E-17 |

| | | | |
|---|---|---|---|
| CHMP4B | NOD1 | -0.124959432 | 0.010007251 |
| CHMP4B | NOD2 | 0.186833753 | 0.000108874 |
| CHMP4B | PLCG1 | 0.215053952 | 7.91E-06 |
| CHMP4B | PRKACA | 0.204973158 | 2.11E-05 |
| CHMP4B | PYCARD | 0.19912013 | 3.64E-05 |
| CHMP4B | SCAF11 | -0.144438571 | 0.002872724 |
| CHMP4B | TP53 | 0.259725757 | 5.77E-08 |
| CHMP4B | TP63 | 0.144212617 | 0.002917245 |
| CHMP4B | TREM2 | -0.286509038 | 1.87E-09 |
| CHMP4B | TIRAP | -0.185752038 | 0.00011952 |
| CHMP4B | TNF | -0.18468968 | 0.000130921 |
| CHMP4B | ZBP1 | 0.224316917 | 3.09E-06 |
| CHMP4C | CHMP6 | -0.164226538 | 0.000686976 |
| CHMP4C | CHMP7 | -0.190897148 | 7.63E-05 |
| CHMP4C | CYCS | 0.256241904 | 8.78E-08 |
| CHMP4C | ELANE | -0.171637899 | 0.000384957 |
| CHMP4C | GPX4 | -0.251730429 | 1.50E-07 |
| CHMP4C | GSDMB | -0.28144018 | 3.69E-09 |
| CHMP4C | GSDMC | 0.328234968 | 4.16E-12 |
| CHMP4C | GSDMD | -0.446456493 | 3.67E-22 |
| CHMP4C | GZMB | -0.243620101 | 3.81E-07 |
| CHMP4C | HMGB1 | 0.265658103 | 2.79E-08 |
| CHMP4C | IL18 | 0.451599378 | 1.07E-22 |
| CHMP4C | IL1A | -0.21297654 | 9.72E-06 |
| CHMP4C | IL1B | -0.462749461 | 6.89E-24 |
| CHMP4C | IL6 | -0.173158712 | 0.00034081 |

| | | | |
|---|---|---|---|
| CHMP4C | IRF1 | -0.492058514 | 3.09E-27 |
| CHMP4C | NLRP3 | -0.336366902 | 1.13E-12 |
| CHMP4C | NLRP6 | -0.277674082 | 6.04E-09 |
| CHMP4C | NLRP7 | -0.239238897 | 6.22E-07 |
| CHMP4C | NLRP9 | 0.13921395 | 0.004077201 |
| CHMP4C | NOD2 | 0.203440107 | 2.43E-05 |
| CHMP4C | PLCG1 | -0.227695853 | 2.17E-06 |
| CHMP4C | PRKACA | -0.396002535 | 2.27E-17 |
| CHMP4C | SCAF11 | 0.2131276 | 9.57E-06 |
| CHMP4C | TP63 | 0.209314869 | 1.39E-05 |
| CHMP4C | TREM2 | -0.173134759 | 0.000341467 |
| CHMP4C | TIRAP | -0.169741648 | 0.000447462 |
| CHMP4C | TNF | -0.203638437 | 2.39E-05 |
| CHMP4C | ZBP1 | -0.587694567 | 9.79E-41 |
| CHMP6 | CHMP7 | 0.181236514 | 0.000175438 |
| CHMP6 | ELANE | 0.336124327 | 1.17E-12 |
| CHMP6 | GPX4 | 0.444730873 | 5.52E-22 |
| CHMP6 | GSDMD | 0.280105793 | 4.40E-09 |
| CHMP6 | HMGB1 | -0.455869073 | 3.79E-23 |
| CHMP6 | IL18 | -0.240431084 | 5.45E-07 |
| CHMP6 | IL1A | -0.332173299 | 2.22E-12 |
| CHMP6 | IRF2 | 0.121300092 | 0.01243391 |
| CHMP6 | NAIP | -0.256302081 | 8.72E-08 |
| CHMP6 | NLRP6 | 0.122553787 | 0.011549623 |
| CHMP6 | NLRP9 | -0.183505034 | 0.000144836 |
| CHMP6 | NOD2 | 0.104111396 | 0.032090332 |

| | | | |
|---|---|---|---|
| CHMP6 | PLCG1 | 0.373005584 | 1.92E-15 |
| CHMP6 | PRKACA | 0.43859825 | 2.32E-21 |
| CHMP6 | PYCARD | 0.378112315 | 7.37E-16 |
| CHMP6 | SCAF11 | -0.29864304 | 3.50E-10 |
| CHMP6 | TP53 | 0.433965312 | 6.71E-21 |
| CHMP6 | TP63 | 0.130507844 | 0.007125838 |
| CHMP6 | TIRAP | -0.183958328 | 0.000139356 |
| CHMP6 | TNF | 0.126543013 | 0.00909449 |
| CHMP6 | GZMA | -0.299326351 | 3.17E-10 |
| CHMP7 | CYCS | -0.14452983 | 0.002854918 |
| CHMP7 | DHX9 | -0.348003671 | 1.62E-13 |
| CHMP7 | ELANE | 0.123392026 | 0.010989843 |
| CHMP7 | GPX4 | 0.297634948 | 4.03E-10 |
| CHMP7 | GSDMC | -0.252749917 | 1.33E-07 |
| CHMP7 | GSDMD | 0.315862488 | 2.81E-11 |
| CHMP7 | GZMB | 0.151239071 | 0.001790226 |
| CHMP7 | HMGB1 | -0.301891866 | 2.20E-10 |
| CHMP7 | IL18 | -0.281730585 | 3.55E-09 |
| CHMP7 | IL1B | 0.112251884 | 0.020783451 |
| CHMP7 | IRF1 | 0.281772838 | 3.53E-09 |
| CHMP7 | IRF2 | 0.187257706 | 0.00010495 |
| CHMP7 | NLRC4 | 0.359661083 | 2.15E-14 |
| CHMP7 | NLRP1 | -0.259821428 | 5.71E-08 |
| CHMP7 | NLRP3 | 0.232913024 | 1.24E-06 |
| CHMP7 | NLRP6 | 0.120319398 | 0.013166754 |
| CHMP7 | NLRP7 | 0.423946925 | 6.34E-20 |

| | | | |
|---|---|---|---|
| CHMP7 | NLRP9 | -0.330999148 | 2.68E-12 |
| CHMP7 | NOD1 | -0.108595564 | 0.025342926 |
| CHMP7 | PLCG1 | 0.12942403 | 0.007622073 |
| CHMP7 | PRKACA | 0.2759806 | 7.53E-09 |
| CHMP7 | PYCARD | 0.138669753 | 0.004225883 |
| CHMP7 | SCAF11 | -0.265683647 | 2.78E-08 |
| CHMP7 | TP63 | -0.4508367 | 1.29E-22 |
| CHMP7 | TREM2 | -0.314664586 | 3.37E-11 |
| CHMP7 | TIRAP | -0.257115788 | 7.91E-08 |
| CHMP7 | TNF | 0.177347519 | 0.000242397 |
| CHMP7 | GZMA | 0.098034557 | 0.043637319 |
| CHMP7 | ZBP1 | 0.226374011 | 2.49E-06 |
| CYCS | DHX9 | 0.125112685 | 0.009915494 |
| CYCS | GPX4 | -0.265523614 | 2.84E-08 |
| CYCS | GSDMC | 0.160918665 | 0.000882812 |
| CYCS | GSDMD | -0.309671994 | 7.10E-11 |
| CYCS | GZMB | -0.137640465 | 0.004520564 |
| CYCS | HMGB1 | 0.267154745 | 2.31E-08 |
| CYCS | IL18 | 0.140427523 | 0.003762536 |
| CYCS | IL1B | -0.201885027 | 2.81E-05 |
| CYCS | IL6 | 0.196899217 | 4.46E-05 |
| CYCS | IRF1 | -0.219773786 | 4.92E-06 |
| CYCS | NAIP | -0.096093706 | 0.047993348 |
| CYCS | NLRP3 | -0.111048878 | 0.022197955 |
| CYCS | NLRP6 | -0.137277334 | 0.004628874 |
| CYCS | NOD1 | -0.126247027 | 0.009259245 |

| | | | |
|---|---|---|---|
| CYCS | NOD2 | 0.192864046 | 6.41E-05 |
| CYCS | PLCG1 | -0.165896657 | 0.000604178 |
| CYCS | PRKACA | -0.243616102 | 3.81E-07 |
| CYCS | PYCARD | -0.09631301 | 0.047483576 |
| CYCS | SCAF11 | 0.233200835 | 1.20E-06 |
| CYCS | GZMA | -0.118893508 | 0.014300134 |
| CYCS | ZBP1 | -0.259107633 | 6.22E-08 |
| DHX9 | GPX4 | -0.250705813 | 1.69E-07 |
| DHX9 | GSDMB | -0.160758273 | 0.000893507 |
| DHX9 | GSDMC | 0.247685948 | 2.39E-07 |
| DHX9 | GSDMD | -0.271284525 | 1.38E-08 |
| DHX9 | GZMB | -0.119247506 | 0.014011032 |
| DHX9 | HMGB1 | 0.156777223 | 0.001200504 |
| DHX9 | IL1A | 0.115296539 | 0.017548106 |
| DHX9 | IL6 | 0.100760456 | 0.038084838 |
| DHX9 | IRF1 | -0.201003143 | 3.06E-05 |
| DHX9 | NLRC4 | -0.11645202 | 0.016440714 |
| DHX9 | NLRP3 | -0.151860696 | 0.001712816 |
| DHX9 | NLRP6 | 0.137042207 | 0.004700247 |
| DHX9 | PRKACA | -0.174635102 | 0.000302507 |
| DHX9 | PYCARD | -0.136905178 | 0.0047423 |
| DHX9 | SCAF11 | 0.290071961 | 1.15E-09 |
| DHX9 | TP63 | 0.601929405 | 3.79E-43 |
| DHX9 | TNF | -0.241982888 | 4.58E-07 |
| ELANE | GPX4 | 0.1528502 | 0.0015959 |
| ELANE | GSDMB | 0.315818677 | 2.83E-11 |

| | | | |
|---|---|---|---|
| ELANE | GSDMC | -0.182157676 | 0.000162346 |
| ELANE | GSDMD | 0.252458897 | 1.37E-07 |
| ELANE | HMGB1 | -0.429974611 | 1.66E-20 |
| ELANE | IL18 | -0.437287598 | 3.13E-21 |
| ELANE | IRF1 | 0.141419094 | 0.003521943 |
| ELANE | IRF2 | 0.228922707 | 1.90E-06 |
| ELANE | NLRC4 | 0.297529898 | 4.09E-10 |
| ELANE | NLRP1 | -0.429583612 | 1.81E-20 |
| ELANE | NLRP3 | 0.144721715 | 0.002817806 |
| ELANE | NLRP6 | 0.114307287 | 0.018547408 |
| ELANE | NLRP7 | 0.424061962 | 6.18E-20 |
| ELANE | NLRP9 | -0.47246593 | 5.80E-25 |
| ELANE | NOD1 | -0.231247845 | 1.49E-06 |
| ELANE | NOD2 | 0.249727981 | 1.89E-07 |
| ELANE | PRKACA | 0.33508289 | 1.39E-12 |
| ELANE | PYCARD | 0.308896244 | 7.95E-11 |
| ELANE | SCAF11 | -0.328034513 | 4.29E-12 |
| ELANE | TP53 | 0.268106986 | 2.05E-08 |
| ELANE | TREM2 | -0.211237219 | 1.15E-05 |
| ELANE | TIRAP | -0.099156734 | 0.041274064 |
| ELANE | TNF | -0.153124353 | 0.001564828 |
| ELANE | ZBP1 | 0.356521322 | 3.74E-14 |
| GPX4 | GSDMC | -0.271359677 | 1.36E-08 |
| GPX4 | GSDMD | 0.395013816 | 2.76E-17 |
| GPX4 | HMGB1 | -0.355522008 | 4.45E-14 |
| GPX4 | IL18 | -0.197335983 | 4.28E-05 |

| | | | |
|---|---|---|---|
| GPX4 | IL1A | -0.132110793 | 0.006444825 |
| GPX4 | IL1B | 0.106111078 | 0.02891219 |
| GPX4 | IL6 | -0.178745356 | 0.000215971 |
| GPX4 | IRF1 | 0.177656272 | 0.000236312 |
| GPX4 | NAIP | -0.133730527 | 0.005816528 |
| GPX4 | NLRP1 | 0.164376122 | 0.000679152 |
| GPX4 | NLRP3 | 0.155043626 | 0.001362378 |
| GPX4 | NLRP6 | 0.198224962 | 3.95E-05 |
| GPX4 | NLRP7 | 0.13136546 | 0.006753869 |
| GPX4 | NOD1 | 0.15443949 | 0.001423346 |
| GPX4 | NOD2 | -0.196830261 | 4.48E-05 |
| GPX4 | PLCG1 | 0.345556583 | 2.46E-13 |
| GPX4 | PRKACA | 0.490426219 | 4.84E-27 |
| GPX4 | PYCARD | 0.412882706 | 6.95E-19 |
| GPX4 | SCAF11 | -0.158703759 | 0.001041504 |
| GPX4 | TP53 | 0.345876739 | 2.33E-13 |
| GPX4 | TP63 | -0.114927489 | 0.017915266 |
| GPX4 | TNF | 0.162482127 | 0.000784584 |
| GPX4 | GZMA | -0.098659176 | 0.042308224 |
| GPX4 | ZBP1 | 0.210402466 | 1.25E-05 |
| GSDMB | GSDMC | -0.276657373 | 6.90E-09 |
| GSDMB | GSDMD | 0.32730305 | 4.81E-12 |
| GSDMB | GZMB | 0.189353593 | 8.74E-05 |
| GSDMB | HMGB1 | -0.192874052 | 6.40E-05 |
| GSDMB | IL18 | -0.236823097 | 8.12E-07 |
| GSDMB | IRF1 | 0.371925794 | 2.34E-15 |

| | | | |
|---|---|---|---|
| GSDMB | IRF2 | 0.113569009 | 0.01932518 |
| GSDMB | NAIP | 0.359161264 | 2.35E-14 |
| GSDMB | NLRC4 | 0.224379177 | 3.07E-06 |
| GSDMB | NLRP1 | -0.293862706 | 6.83E-10 |
| GSDMB | NLRP7 | 0.328060948 | 4.27E-12 |
| GSDMB | NLRP9 | -0.368802252 | 4.16E-15 |
| GSDMB | NOD1 | -0.321270872 | 1.23E-11 |
| GSDMB | PRKACA | 0.149038956 | 0.002090652 |
| GSDMB | PYCARD | 0.119041779 | 0.014178413 |
| GSDMB | TP63 | -0.350741384 | 1.02E-13 |
| GSDMB | TIRAP | 0.269687514 | 1.68E-08 |
| GSDMB | TNF | -0.095859574 | 0.048542637 |
| GSDMB | GZMA | 0.29886189 | 3.39E-10 |
| GSDMB | ZBP1 | 0.514976048 | 4.33E-30 |
| GSDMC | GSDMD | -0.21042352 | 1.25E-05 |
| GSDMC | GZMB | -0.146815559 | 0.002440432 |
| GSDMC | HMGB1 | 0.212590229 | 1.01E-05 |
| GSDMC | IL18 | 0.383315102 | 2.74E-16 |
| GSDMC | IL1A | -0.16102263 | 0.000875943 |
| GSDMC | IL1B | -0.236878876 | 8.07E-07 |
| GSDMC | IL6 | -0.117671599 | 0.015338717 |
| GSDMC | IRF1 | -0.457093468 | 2.81E-23 |
| GSDMC | IRF2 | -0.222594946 | 3.69E-06 |
| GSDMC | NAIP | -0.221704352 | 4.04E-06 |
| GSDMC | NLRC4 | -0.21742636 | 6.24E-06 |
| GSDMC | NLRP1 | 0.157756582 | 0.001117077 |

| | | | |
|---|---|---|---|
| GSDMC | NLRP2 | 0.112607372 | 0.020380743 |
| GSDMC | NLRP3 | -0.342941601 | 3.81E-13 |
| GSDMC | NLRP6 | -0.155349503 | 0.001332433 |
| GSDMC | NLRP7 | -0.366861723 | 5.92E-15 |
| GSDMC | NLRP9 | 0.213271074 | 9.44E-06 |
| GSDMC | NOD1 | 0.235728865 | 9.16E-07 |
| GSDMC | NOD2 | 0.147663324 | 0.002301211 |
| GSDMC | PRKACA | -0.312160837 | 4.90E-11 |
| GSDMC | PYCARD | -0.12442693 | 0.010331957 |
| GSDMC | SCAF11 | 0.23917542 | 6.26E-07 |
| GSDMC | TP63 | 0.392328363 | 4.72E-17 |
| GSDMC | TIRAP | -0.201044682 | 3.04E-05 |
| GSDMC | GZMA | -0.207110173 | 1.72E-05 |
| GSDMC | ZBP1 | -0.4865665 | 1.39E-26 |
| GSDMD | GZMB | 0.26150059 | 4.65E-08 |
| GSDMD | HMGB1 | -0.40456848 | 3.96E-18 |
| GSDMD | IL18 | -0.426703153 | 3.44E-20 |
| GSDMD | IL1B | 0.23981058 | 5.84E-07 |
| GSDMD | IRF1 | 0.339964226 | 6.24E-13 |
| GSDMD | IRF2 | -0.155783502 | 0.001290983 |
| GSDMD | NLRC4 | 0.225106784 | 2.84E-06 |
| GSDMD | NLRP1 | -0.225711628 | 2.67E-06 |
| GSDMD | NLRP3 | 0.173517513 | 0.000331106 |
| GSDMD | NLRP6 | 0.201177048 | 3.01E-05 |
| GSDMD | NLRP7 | 0.315846594 | 2.82E-11 |
| GSDMD | NLRP9 | -0.28994426 | 1.17E-09 |

| | | | |
|---|---|---|---|
| GSDMD | NOD1 | -0.117145531 | 0.015805859 |
| GSDMD | NOD2 | -0.112866729 | 0.020091231 |
| GSDMD | PLCG1 | 0.306110651 | 1.20E-10 |
| GSDMD | PRKACA | 0.239907055 | 5.78E-07 |
| GSDMD | PYCARD | 0.225988183 | 2.59E-06 |
| GSDMD | SCAF11 | -0.157753462 | 0.001117334 |
| GSDMD | TP63 | -0.384187896 | 2.31E-16 |
| GSDMD | TIRAP | -0.154835211 | 0.001383134 |
| GSDMD | ZBP1 | 0.412425737 | 7.65E-19 |
| GZMB | IL18 | -0.148491604 | 0.002172227 |
| GZMB | IL1A | 0.209177744 | 1.41E-05 |
| GZMB | IL1B | 0.243716864 | 3.77E-07 |
| GZMB | IL6 | 0.246095351 | 2.87E-07 |
| GZMB | IRF1 | 0.377739273 | 7.91E-16 |
| GZMB | IRF2 | -0.213951812 | 8.83E-06 |
| GZMB | NAIP | 0.157131343 | 0.001169697 |
| GZMB | NLRP2 | -0.108801425 | 0.02506501 |
| GZMB | NLRP3 | 0.235897058 | 8.99E-07 |
| GZMB | NOD2 | -0.138253836 | 0.004342802 |
| GZMB | PRKACA | -0.110501569 | 0.022868621 |
| GZMB | TP53 | -0.261458636 | 4.68E-08 |
| GZMB | TP63 | -0.255929376 | 9.11E-08 |
| GZMB | TNF | 0.187017857 | 0.000107153 |
| GZMB | GZMA | 0.352523638 | 7.49E-14 |
| GZMB | ZBP1 | 0.191898136 | 6.99E-05 |
| HMGB1 | IL18 | 0.343211146 | 3.64E-13 |

| | | | |
|---|---|---|---|
| HMGB1 | IL1B | -0.14864858 | 0.00214854 |
| HMGB1 | IRF1 | -0.277970935 | 5.81E-09 |
| HMGB1 | IRF2 | -0.150387822 | 0.001901433 |
| HMGB1 | NAIP | 0.106246766 | 0.028706679 |
| HMGB1 | NLRC4 | -0.224607379 | 3.00E-06 |
| HMGB1 | NLRP1 | 0.123679978 | 0.010803168 |
| HMGB1 | NLRP3 | -0.153819923 | 0.001488467 |
| HMGB1 | NLRP6 | -0.135866453 | 0.005072264 |
| HMGB1 | NLRP7 | -0.217262532 | 6.35E-06 |
| HMGB1 | NLRP9 | 0.372926578 | 1.95E-15 |
| HMGB1 | NOD2 | -0.189770163 | 8.43E-05 |
| HMGB1 | PLCG1 | -0.276341533 | 7.19E-09 |
| HMGB1 | PRKACA | -0.504995053 | 8.04E-29 |
| HMGB1 | PYCARD | -0.43359935 | 7.29E-21 |
| HMGB1 | SCAF11 | 0.486492716 | 1.41E-26 |
| HMGB1 | TP53 | -0.336378971 | 1.12E-12 |
| HMGB1 | ZBP1 | -0.287688375 | 1.60E-09 |
| IL18 | IL1B | -0.220547491 | 4.55E-06 |
| IL18 | IL6 | -0.289405748 | 1.26E-09 |
| IL18 | IRF1 | -0.46350525 | 5.70E-24 |
| IL18 | IRF2 | -0.205520525 | 2.00E-05 |
| IL18 | NLRC4 | -0.310970566 | 5.85E-11 |
| IL18 | NLRP1 | 0.340450052 | 5.76E-13 |
| IL18 | NLRP2 | 0.136072613 | 0.005005175 |
| IL18 | NLRP3 | -0.344729488 | 2.82E-13 |
| IL18 | NLRP6 | -0.311778356 | 5.19E-11 |

| | | | |
|---|---|---|---|
| IL18 | NLRP7 | -0.506791614 | 4.79E-29 |
| IL18 | NLRP9 | 0.31004937 | 6.71E-11 |
| IL18 | NOD1 | 0.166880214 | 0.00055985 |
| IL18 | PLCG1 | -0.158816334 | 0.001032842 |
| IL18 | PRKACA | -0.246038161 | 2.89E-07 |
| IL18 | SCAF11 | 0.429655497 | 1.78E-20 |
| IL18 | TP53 | -0.130827118 | 0.006985272 |
| IL18 | TP63 | 0.233172454 | 1.21E-06 |
| IL18 | GZMA | -0.104497294 | 0.03145477 |
| IL18 | ZBP1 | -0.537016104 | 4.79E-33 |
| IL1A | IL1B | 0.538579366 | 2.90E-33 |
| IL1A | IL6 | 0.391664846 | 5.38E-17 |
| IL1A | IRF1 | 0.300502284 | 2.68E-10 |
| IL1A | NAIP | 0.297464523 | 4.13E-10 |
| IL1A | NLRP1 | -0.126612853 | 0.009055998 |
| IL1A | NLRP3 | 0.193084609 | 6.29E-05 |
| IL1A | NOD1 | -0.118995369 | 0.014216414 |
| IL1A | PLCG1 | -0.290723297 | 1.05E-09 |
| IL1A | PYCARD | -0.288397015 | 1.45E-09 |
| IL1A | TP53 | -0.260397896 | 5.32E-08 |
| IL1A | TP63 | -0.107025139 | 0.027551828 |
| IL1A | GZMA | 0.120658882 | 0.012908871 |
| IL1B | IL6 | 0.424230008 | 5.95E-20 |
| IL1B | IRF1 | 0.371123257 | 2.72E-15 |
| IL1B | NAIP | 0.163771117 | 0.000711312 |
| IL1B | NLRC4 | 0.14013208 | 0.003837046 |

| | | | |
|---|---|---|---|
| IL1B | NLRP3 | 0.425265835 | 4.74E-20 |
| IL1B | NLRP6 | 0.206449434 | 1.83E-05 |
| IL1B | NLRP7 | 0.125977438 | 0.00941161 |
| IL1B | NOD2 | -0.232673951 | 1.28E-06 |
| IL1B | PRKACA | 0.122175382 | 0.01181048 |
| IL1B | PYCARD | -0.14511717 | 0.002742703 |
| IL1B | TP53 | -0.156564202 | 0.001219393 |
| IL1B | TP63 | -0.139717376 | 0.003943887 |
| IL1B | TREM2 | 0.096048977 | 0.048097879 |
| IL1B | TNF | 0.12017646 | 0.013276685 |
| IL1B | ZBP1 | 0.367371408 | 5.40E-15 |
| IL6 | IRF1 | 0.341096796 | 5.18E-13 |
| IL6 | NAIP | 0.108641529 | 0.025280644 |
| IL6 | NLRC4 | 0.095762046 | 0.04877299 |
| IL6 | NLRP1 | -0.201583861 | 2.89E-05 |
| IL6 | NLRP3 | 0.300860874 | 2.55E-10 |
| IL6 | NLRP6 | 0.115877769 | 0.016983178 |
| IL6 | NLRP9 | -0.12946959 | 0.007600607 |
| IL6 | NOD2 | 0.173391308 | 0.000334489 |
| IL6 | PLCG1 | -0.242255117 | 4.44E-07 |
| IL6 | PYCARD | -0.143220772 | 0.003120196 |
| IL6 | SCAF11 | -0.154797912 | 0.00138688 |
| IL6 | TNF | 0.113814336 | 0.019063636 |
| IL6 | ZBP1 | 0.102451785 | 0.034950131 |
| IRF1 | IRF2 | 0.181922966 | 0.000165592 |
| IRF1 | NAIP | 0.332721325 | 2.03E-12 |

| | | | |
|---|---|---|---|
| IRF1 | NLRC4 | 0.234247656 | 1.08E-06 |
| IRF1 | NLRP1 | -0.149733936 | 0.001991111 |
| IRF1 | NLRP3 | 0.373514358 | 1.74E-15 |
| IRF1 | NLRP6 | 0.192510008 | 6.62E-05 |
| IRF1 | NLRP7 | 0.314329773 | 3.55E-11 |
| IRF1 | NLRP9 | -0.177576903 | 0.000237863 |
| IRF1 | NOD1 | -0.130113496 | 0.007302947 |
| IRF1 | PRKACA | 0.196897759 | 4.46E-05 |
| IRF1 | SCAF11 | -0.19427116 | 5.65E-05 |
| IRF1 | TP63 | -0.32344325 | 8.81E-12 |
| IRF1 | TIRAP | 0.181262973 | 0.000175048 |
| IRF1 | TNF | 0.170183523 | 0.000432106 |
| IRF1 | GZMA | 0.318168984 | 1.98E-11 |
| IRF1 | ZBP1 | 0.510754199 | 1.51E-29 |
| IRF2 | NAIP | 0.154960665 | 0.001370606 |
| IRF2 | NLRC4 | 0.132870574 | 0.006142899 |
| IRF2 | NLRP1 | -0.182975093 | 0.0001515 |
| IRF2 | NLRP6 | 0.0956943 | 0.048933538 |
| IRF2 | NLRP7 | 0.219173476 | 5.23E-06 |
| IRF2 | NLRP9 | -0.148270244 | 0.002206036 |
| IRF2 | NOD1 | -0.20542182 | 2.02E-05 |
| IRF2 | NOD2 | 0.154343314 | 0.001433281 |
| IRF2 | PLCG1 | -0.204550866 | 2.19E-05 |
| IRF2 | PRKACA | 0.495846276 | 1.08E-27 |
| IRF2 | SCAF11 | -0.288862992 | 1.36E-09 |
| IRF2 | TP53 | 0.572894995 | 2.37E-38 |

| | | | |
|---|---|---|---|
| IRF2 | TP63 | 0.152651825 | 0.001618735 |
| IRF2 | TREM2 | -0.113412025 | 0.019494176 |
| IRF2 | TIRAP | 0.23859443 | 6.68E-07 |
| IRF2 | ZBP1 | 0.142263768 | 0.003328091 |
| NAIP | NLRC4 | 0.167553417 | 0.000531271 |
| NAIP | NLRP1 | -0.176113681 | 0.000268204 |
| NAIP | NLRP6 | -0.157651402 | 0.001125773 |
| NAIP | NLRP7 | 0.172786692 | 0.00035115 |
| NAIP | NLRP9 | -0.163857038 | 0.00070666 |
| NAIP | NOD1 | -0.284113049 | 2.58E-09 |
| NAIP | PLCG1 | -0.334216686 | 1.60E-12 |
| NAIP | SCAF11 | 0.175407241 | 0.000284113 |
| NAIP | TP53 | -0.131291924 | 0.006785067 |
| NAIP | TP63 | -0.17321841 | 0.000339177 |
| NAIP | TREM2 | -0.183758579 | 0.000141746 |
| NAIP | TIRAP | 0.178551692 | 0.000219464 |
| NAIP | GZMA | 0.52149035 | 6.09E-31 |
| NAIP | ZBP1 | 0.1900461 | 8.23E-05 |
| NLRC4 | NLRP1 | -0.46226916 | 7.77E-24 |
| NLRC4 | NLRP3 | 0.188226898 | 9.65E-05 |
| NLRC4 | NLRP6 | 0.095838117 | 0.048593238 |
| NLRC4 | NLRP7 | 0.497346168 | 7.08E-28 |
| NLRC4 | NLRP9 | -0.384248779 | 2.29E-16 |
| NLRC4 | NOD1 | -0.224733024 | 2.96E-06 |
| NLRC4 | NOD2 | 0.098967115 | 0.041665638 |
| NLRC4 | PYCARD | 0.13779957 | 0.004473834 |

| | | | |
|---|---|---|---|
| NLRC4 | SCAF11 | -0.19453731 | 5.52E-05 |
| NLRC4 | TP63 | -0.285056662 | 2.28E-09 |
| NLRC4 | TREM2 | -0.357089112 | 3.39E-14 |
| NLRC4 | TIRAP | -0.149398592 | 0.002038588 |
| NLRC4 | GZMA | 0.244355859 | 3.50E-07 |
| NLRC4 | ZBP1 | 0.290265388 | 1.12E-09 |
| NLRP1 | NLRP2 | -0.096281108 | 0.047557448 |
| NLRP1 | NLRP3 | -0.128507721 | 0.008065454 |
| NLRP1 | NLRP7 | -0.574951656 | 1.13E-38 |
| NLRP1 | NLRP9 | 0.52287462 | 4.00E-31 |
| NLRP1 | NOD1 | 0.550275296 | 6.23E-35 |
| NLRP1 | NOD2 | -0.295400973 | 5.52E-10 |
| NLRP1 | PLCG1 | 0.269898316 | 1.64E-08 |
| NLRP1 | SCAF11 | 0.210402135 | 1.25E-05 |
| NLRP1 | TP53 | 0.146043486 | 0.002573862 |
| NLRP1 | TP63 | 0.246645968 | 2.70E-07 |
| NLRP1 | TREM2 | 0.527238044 | 1.04E-31 |
| NLRP1 | TIRAP | 0.293542576 | 7.14E-10 |
| NLRP1 | TNF | 0.185916648 | 0.000117839 |
| NLRP1 | GZMA | -0.250224908 | 1.78E-07 |
| NLRP1 | ZBP1 | -0.280267202 | 4.30E-09 |
| NLRP2 | NLRP7 | 0.120847996 | 0.012767153 |
| NLRP2 | PRKACA | 0.102704682 | 0.034500819 |
| NLRP2 | PYCARD | 0.098130359 | 0.043431214 |
| NLRP2 | TP53 | 0.104468927 | 0.031501119 |
| NLRP2 | TREM2 | -0.111185816 | 0.022032843 |

| | | | |
|---|---|---|---|
| NLRP3 | NLRP6 | 0.193450781 | 6.08E-05 |
| NLRP3 | NLRP7 | 0.234098765 | 1.09E-06 |
| NLRP3 | PRKACA | 0.180422415 | 0.000187826 |
| NLRP3 | SCAF11 | -0.283798276 | 2.69E-09 |
| NLRP3 | TP53 | -0.102306127 | 0.035211168 |
| NLRP3 | TP63 | -0.259793441 | 5.73E-08 |
| NLRP3 | TNF | 0.13381938 | 0.00578371 |
| NLRP3 | ZBP1 | 0.31102384 | 5.81E-11 |
| NLRP6 | NLRP7 | 0.164344716 | 0.000680788 |
| NLRP6 | NOD2 | -0.216041175 | 7.17E-06 |
| NLRP6 | PLCG1 | 0.177261964 | 0.000244109 |
| NLRP6 | PRKACA | 0.261070388 | 4.90E-08 |
| NLRP6 | SCAF11 | -0.157907429 | 0.001104712 |
| NLRP6 | TP53 | 0.169429696 | 0.000458607 |
| NLRP6 | TREM2 | 0.169836627 | 0.000444119 |
| NLRP6 | GZMA | -0.124209595 | 0.010467145 |
| NLRP6 | ZBP1 | 0.395416482 | 2.55E-17 |
| NLRP7 | NLRP9 | -0.545718798 | 2.83E-34 |
| NLRP7 | NOD1 | -0.364123315 | 9.71E-15 |
| NLRP7 | PRKACA | 0.175657443 | 0.00027838 |
| NLRP7 | PYCARD | 0.164923145 | 0.00065124 |
| NLRP7 | SCAF11 | -0.246034016 | 2.89E-07 |
| NLRP7 | TP63 | -0.378311297 | 7.10E-16 |
| NLRP7 | TREM2 | -0.379174099 | 6.03E-16 |
| NLRP7 | TIRAP | -0.16867641 | 0.000486593 |
| NLRP7 | TNF | -0.205325561 | 2.04E-05 |

| | | | |
|---|---|---|---|
| NLRP7 | GZMA | 0.294371449 | 6.37E-10 |
| NLRP7 | ZBP1 | 0.571985681 | 3.30E-38 |
| NLRP9 | NOD1 | 0.335464277 | 1.30E-12 |
| NLRP9 | NOD2 | -0.199030737 | 3.67E-05 |
| NLRP9 | PRKACA | -0.189824268 | 8.39E-05 |
| NLRP9 | PYCARD | -0.293078175 | 7.62E-10 |
| NLRP9 | SCAF11 | 0.114755982 | 0.018088165 |
| NLRP9 | TP53 | -0.113914203 | 0.018958054 |
| NLRP9 | TP63 | 0.186292851 | 0.000114081 |
| NLRP9 | TREM2 | 0.472171841 | 6.26E-25 |
| NLRP9 | TIRAP | 0.261087849 | 4.89E-08 |
| NLRP9 | TNF | 0.128775966 | 0.007933333 |
| NLRP9 | GZMA | -0.242242223 | 4.45E-07 |
| NLRP9 | ZBP1 | -0.367317283 | 5.45E-15 |
| NOD1 | NOD2 | -0.202601284 | 2.63E-05 |
| NOD1 | PLCG1 | 0.313260471 | 4.16E-11 |
| NOD1 | TP63 | 0.218472889 | 5.62E-06 |
| NOD1 | TREM2 | 0.326290683 | 5.65E-12 |
| NOD1 | TNF | 0.104073065 | 0.032154056 |
| NOD1 | GZMA | -0.182390181 | 0.000159189 |
| NOD1 | ZBP1 | -0.147561791 | 0.002317494 |
| NOD2 | PLCG1 | -0.246865933 | 2.63E-07 |
| NOD2 | PYCARD | 0.221469305 | 4.14E-06 |
| NOD2 | SCAF11 | -0.098547441 | 0.042543443 |
| NOD2 | TP63 | 0.118700797 | 0.014459711 |
| NOD2 | TREM2 | -0.391420623 | 5.64E-17 |

| | | | |
|---|---|---|---|
| NOD2 | TIRAP | -0.19228191 | 6.75E-05 |
| NOD2 | ZBP1 | -0.235326407 | 9.57E-07 |
| PLCG1 | PRKACA | 0.169466802 | 0.000457268 |
| PLCG1 | PYCARD | 0.148665764 | 0.002145961 |
| PLCG1 | TP53 | 0.139262343 | 0.004064211 |
| PLCG1 | TREM2 | 0.264620599 | 3.17E-08 |
| PLCG1 | TIRAP | -0.136860363 | 0.004756126 |
| PLCG1 | GZMA | -0.217415114 | 6.25E-06 |
| PLCG1 | ZBP1 | 0.117649786 | 0.015357844 |
| PRKACA | PYCARD | 0.250013746 | 1.83E-07 |
| PRKACA | SCAF11 | -0.40759903 | 2.11E-18 |
| PRKACA | TP53 | 0.566961925 | 1.98E-37 |
| PRKACA | TIRAP | 0.139866833 | 0.003905076 |
| PRKACA | GZMA | -0.209244405 | 1.40E-05 |
| PRKACA | ZBP1 | 0.335956361 | 1.20E-12 |
| PYCARD | TP53 | 0.303689833 | 1.70E-10 |
| PYCARD | TREM2 | -0.209989312 | 1.30E-05 |
| PYCARD | TIRAP | -0.1648411 | 0.000655357 |
| SCAF11 | TP53 | -0.276899277 | 6.69E-09 |
| SCAF11 | TP63 | 0.13222633 | 0.006398071 |
| SCAF11 | ZBP1 | -0.172832376 | 0.000349865 |
| TP53 | TP63 | 0.435008404 | 5.29E-21 |
| TP53 | TREM2 | 0.108047364 | 0.026096004 |
| TP53 | TIRAP | 0.259080409 | 6.24E-08 |
| TP53 | GZMA | -0.229521943 | 1.79E-06 |
| TP63 | TREM2 | 0.196709175 | 4.53E-05 |

| | | | |
|---|---|---|---|
| TP63 | TNF | -0.134797367 | 0.005433333 |
| TP63 | GZMA | -0.235772695 | 9.11E-07 |
| TP63 | ZBP1 | -0.355347304 | 4.59E-14 |
| TREM2 | TIRAP | 0.45555979 | 4.09E-23 |
| TREM2 | GZMA | -0.27354186 | 1.03E-08 |
| TIRAP | ZBP1 | 0.176992839 | 0.000249567 |
| TNF | ZBP1 | -0.12242575 | 0.01163731 |
| GZMA | ZBP1 | 0.219771844 | 4.92E-06 |

**Table S5. The 3 distinct PRG patterns based on 48 significant PRGs**

| Samples | Subtype |
|---|---|
| GSM261086 | cluster A |
| GSM261089 | cluster A |
| GSM261090 | cluster A |
| GSM261097 | cluster A |
| GSM261098 | cluster A |
| GSM261100 | cluster A |
| GSM261105 | cluster A |
| GSM261114 | cluster A |
| GSM261115 | cluster A |
| GSM261120 | cluster A |
| GSM261125 | cluster A |
| GSM261126 | cluster A |
| GSM261128 | cluster A |
| GSM261129 | cluster A |
| GSM261136 | cluster A |

| | |
|---|---|
| GSM261145 | cluster A |
| GSM261164 | cluster A |
| GSM261175 | cluster A |
| GSM261176 | cluster A |
| GSM261178 | cluster A |
| GSM261179 | cluster A |
| GSM261180 | cluster A |
| GSM261186 | cluster A |
| GSM261189 | cluster A |
| GSM261191 | cluster A |
| GSM261192 | cluster A |
| GSM261194 | cluster A |
| GSM261195 | cluster A |
| GSM261197 | cluster A |
| GSM261198 | cluster A |
| GSM261200 | cluster A |
| GSM261208 | cluster A |
| GSM261216 | cluster A |
| GSM261218 | cluster A |
| GSM261219 | cluster A |
| GSM261225 | cluster A |
| GSM261238 | cluster A |
| GSM261239 | cluster A |
| GSM261247 | cluster A |
| GSM261248 | cluster A |
| GSM261250 | cluster A |

| | |
|---|---|
| GSM261253 | cluster A |
| GSM261255 | cluster A |
| GSM261261 | cluster A |
| GSM261262 | cluster A |
| GSM261263 | cluster A |
| GSM261264 | cluster A |
| GSM261265 | cluster A |
| GSM261267 | cluster A |
| GSM261268 | cluster A |
| GSM261269 | cluster A |
| GSM261270 | cluster A |
| GSM261281 | cluster A |
| GSM261282 | cluster A |
| GSM261284 | cluster A |
| GSM261285 | cluster A |
| GSM261294 | cluster A |
| GSM261297 | cluster A |
| GSM261298 | cluster A |
| GSM261300 | cluster A |
| GSM261301 | cluster A |
| GSM261302 | cluster A |
| GSM261304 | cluster A |
| GSM261307 | cluster A |
| GSM261308 | cluster A |
| GSM261310 | cluster A |
| GSM261316 | cluster A |

| | |
|---|---|
| GSM261319 | cluster A |
| GSM261324 | cluster A |
| GSM261325 | cluster A |
| GSM261328 | cluster A |
| GSM261331 | cluster A |
| GSM404005 | cluster A |
| GSM404008 | cluster A |
| GSM404009 | cluster A |
| GSM404012 | cluster A |
| GSM404014 | cluster A |
| GSM404015 | cluster A |
| GSM404019 | cluster A |
| GSM404023 | cluster A |
| GSM404029 | cluster A |
| GSM404030 | cluster A |
| GSM404034 | cluster A |
| GSM404037 | cluster A |
| GSM404038 | cluster A |
| GSM404040 | cluster A |
| GSM404045 | cluster A |
| GSM404057 | cluster A |
| GSM404062 | cluster A |
| GSM404063 | cluster A |
| GSM404065 | cluster A |
| GSM404066 | cluster A |
| GSM404073 | cluster A |

| | |
|---|---|
| GSM404098 | cluster A |
| GSM404109 | cluster A |
| GSM404110 | cluster A |
| GSM404111 | cluster A |
| GSM404118 | cluster A |
| GSM404121 | cluster A |
| GSM404123 | cluster A |
| GSM404124 | cluster A |
| GSM404126 | cluster A |
| GSM404127 | cluster A |
| GSM404129 | cluster A |
| GSM404144 | cluster A |
| GSM404145 | cluster A |
| GSM404151 | cluster A |
| GSM404164 | cluster A |
| GSM404165 | cluster A |
| GSM404173 | cluster A |
| GSM404174 | cluster A |
| GSM404176 | cluster A |
| GSM404178 | cluster A |
| GSM404179 | cluster A |
| GSM404181 | cluster A |
| GSM404184 | cluster A |
| GSM404185 | cluster A |
| GSM404186 | cluster A |
| GSM404187 | cluster A |

| | |
|---|---|
| GSM404188 | cluster A |
| GSM404189 | cluster A |
| GSM404191 | cluster A |
| GSM404192 | cluster A |
| GSM404193 | cluster A |
| GSM404204 | cluster A |
| GSM404205 | cluster A |
| GSM404213 | cluster A |
| GSM404214 | cluster A |
| GSM404216 | cluster A |
| GSM404217 | cluster A |
| GSM404218 | cluster A |
| GSM404219 | cluster A |
| GSM404220 | cluster A |
| GSM404221 | cluster A |
| GSM404223 | cluster A |
| GSM404226 | cluster A |
| GSM404227 | cluster A |
| GSM404229 | cluster A |
| GSM404232 | cluster A |
| GSM404235 | cluster A |
| GSM404240 | cluster A |
| GSM404241 | cluster A |
| GSM404243 | cluster A |
| GSM404244 | cluster A |
| GSM404247 | cluster A |

| | |
|---|---|
| GSM404252 | cluster A |
| GSM404253 | cluster A |
| GSM404254 | cluster A |
| GSM404256 | cluster A |
| GSM404258 | cluster A |
| GSM404259 | cluster A |
| GSM404261 | cluster A |
| GSM404262 | cluster A |
| GSM404263 | cluster A |
| GSM404264 | cluster A |
| GSM404265 | cluster A |
| GSM404266 | cluster A |
| GSM404272 | cluster A |
| GSM404274 | cluster A |
| GSM404275 | cluster A |
| GSM404278 | cluster A |
| GSM404279 | cluster A |
| GSM404286 | cluster A |
| GSM404294 | cluster A |
| GSM404297 | cluster A |
| GSM404299 | cluster A |
| GSM404301 | cluster A |
| GSM404302 | cluster A |
| GSM404304 | cluster A |
| GSM404307 | cluster A |
| GSM404309 | cluster A |

| | |
|---|---|
| GSM404310 | cluster A |
| GSM404311 | cluster A |
| GSM404313 | cluster A |
| GSM404314 | cluster A |
| GSM261087 | cluster B |
| GSM261092 | cluster B |
| GSM261093 | cluster B |
| GSM261101 | cluster B |
| GSM261103 | cluster B |
| GSM261104 | cluster B |
| GSM261106 | cluster B |
| GSM261107 | cluster B |
| GSM261108 | cluster B |
| GSM261110 | cluster B |
| GSM261111 | cluster B |
| GSM261112 | cluster B |
| GSM261119 | cluster B |
| GSM261121 | cluster B |
| GSM261130 | cluster B |
| GSM261131 | cluster B |
| GSM261132 | cluster B |
| GSM261133 | cluster B |
| GSM261139 | cluster B |
| GSM261140 | cluster B |
| GSM261141 | cluster B |
| GSM261142 | cluster B |

| | |
|---|---|
| GSM261148 | cluster B |
| GSM261149 | cluster B |
| GSM261150 | cluster B |
| GSM261153 | cluster B |
| GSM261155 | cluster B |
| GSM261156 | cluster B |
| GSM261157 | cluster B |
| GSM261158 | cluster B |
| GSM261160 | cluster B |
| GSM261161 | cluster B |
| GSM261163 | cluster B |
| GSM261166 | cluster B |
| GSM261167 | cluster B |
| GSM261169 | cluster B |
| GSM261170 | cluster B |
| GSM261172 | cluster B |
| GSM261173 | cluster B |
| GSM261181 | cluster B |
| GSM261183 | cluster B |
| GSM261187 | cluster B |
| GSM261203 | cluster B |
| GSM261204 | cluster B |
| GSM261207 | cluster B |
| GSM261209 | cluster B |
| GSM261210 | cluster B |
| GSM261211 | cluster B |

| | |
|---|---|
| GSM261212 | cluster B |
| GSM261214 | cluster B |
| GSM261256 | cluster B |
| GSM261258 | cluster B |
| GSM261259 | cluster B |
| GSM261260 | cluster B |
| GSM261276 | cluster B |
| GSM261278 | cluster B |
| GSM261279 | cluster B |
| GSM261312 | cluster B |
| GSM261313 | cluster B |
| GSM404006 | cluster B |
| GSM404018 | cluster B |
| GSM404020 | cluster B |
| GSM404021 | cluster B |
| GSM404022 | cluster B |
| GSM404024 | cluster B |
| GSM404026 | cluster B |
| GSM404027 | cluster B |
| GSM404032 | cluster B |
| GSM404033 | cluster B |
| GSM404041 | cluster B |
| GSM404043 | cluster B |
| GSM404044 | cluster B |
| GSM404046 | cluster B |
| GSM404047 | cluster B |

| | |
|---|---|
| GSM404048 | cluster B |
| GSM404050 | cluster B |
| GSM404051 | cluster B |
| GSM404052 | cluster B |
| GSM404055 | cluster B |
| GSM404056 | cluster B |
| GSM404058 | cluster B |
| GSM404067 | cluster B |
| GSM404068 | cluster B |
| GSM404069 | cluster B |
| GSM404070 | cluster B |
| GSM404076 | cluster B |
| GSM404077 | cluster B |
| GSM404078 | cluster B |
| GSM404079 | cluster B |
| GSM404082 | cluster B |
| GSM404083 | cluster B |
| GSM404084 | cluster B |
| GSM404087 | cluster B |
| GSM404089 | cluster B |
| GSM404090 | cluster B |
| GSM404091 | cluster B |
| GSM404092 | cluster B |
| GSM404094 | cluster B |
| GSM404095 | cluster B |
| GSM404097 | cluster B |

| | |
|---|---|
| GSM404100 | cluster B |
| GSM404101 | cluster B |
| GSM404103 | cluster B |
| GSM404104 | cluster B |
| GSM404106 | cluster B |
| GSM404107 | cluster B |
| GSM404112 | cluster B |
| GSM404114 | cluster B |
| GSM404116 | cluster B |
| GSM404120 | cluster B |
| GSM404132 | cluster B |
| GSM404133 | cluster B |
| GSM404135 | cluster B |
| GSM404136 | cluster B |
| GSM404137 | cluster B |
| GSM404138 | cluster B |
| GSM404140 | cluster B |
| GSM404141 | cluster B |
| GSM404142 | cluster B |
| GSM404156 | cluster B |
| GSM404182 | cluster B |
| GSM404199 | cluster B |
| GSM404201 | cluster B |
| GSM404202 | cluster B |
| GSM404300 | cluster B |
| GSM261094 | cluster C |

| | |
|---|---|
| GSM261095 | cluster C |
| GSM261118 | cluster C |
| GSM261123 | cluster C |
| GSM261124 | cluster C |
| GSM261135 | cluster C |
| GSM261144 | cluster C |
| GSM261147 | cluster C |
| GSM261152 | cluster C |
| GSM261182 | cluster C |
| GSM261185 | cluster C |
| GSM261190 | cluster C |
| GSM261201 | cluster C |
| GSM261213 | cluster C |
| GSM261215 | cluster C |
| GSM261221 | cluster C |
| GSM261222 | cluster C |
| GSM261224 | cluster C |
| GSM261227 | cluster C |
| GSM261228 | cluster C |
| GSM261230 | cluster C |
| GSM261231 | cluster C |
| GSM261232 | cluster C |
| GSM261233 | cluster C |
| GSM261235 | cluster C |
| GSM261236 | cluster C |
| GSM261241 | cluster C |

| | |
|---|---|
| GSM261242 | cluster C |
| GSM261244 | cluster C |
| GSM261245 | cluster C |
| GSM261251 | cluster C |
| GSM261252 | cluster C |
| GSM261271 | cluster C |
| GSM261273 | cluster C |
| GSM261274 | cluster C |
| GSM261275 | cluster C |
| GSM261287 | cluster C |
| GSM261288 | cluster C |
| GSM261289 | cluster C |
| GSM261290 | cluster C |
| GSM261291 | cluster C |
| GSM261292 | cluster C |
| GSM261295 | cluster C |
| GSM261299 | cluster C |
| GSM261305 | cluster C |
| GSM261311 | cluster C |
| GSM261315 | cluster C |
| GSM261318 | cluster C |
| GSM261321 | cluster C |
| GSM261322 | cluster C |
| GSM261327 | cluster C |
| GSM261330 | cluster C |
| GSM404011 | cluster C |

| | |
|---|---|
| GSM404035 | cluster C |
| GSM404060 | cluster C |
| GSM404061 | cluster C |
| GSM404072 | cluster C |
| GSM404081 | cluster C |
| GSM404086 | cluster C |
| GSM404113 | cluster C |
| GSM404119 | cluster C |
| GSM404130 | cluster C |
| GSM404139 | cluster C |
| GSM404147 | cluster C |
| GSM404148 | cluster C |
| GSM404150 | cluster C |
| GSM404153 | cluster C |
| GSM404154 | cluster C |
| GSM404157 | cluster C |
| GSM404158 | cluster C |
| GSM404159 | cluster C |
| GSM404161 | cluster C |
| GSM404162 | cluster C |
| GSM404167 | cluster C |
| GSM404168 | cluster C |
| GSM404170 | cluster C |
| GSM404171 | cluster C |
| GSM404177 | cluster C |
| GSM404194 | cluster C |

| | |
|---|---|
| GSM404196 | cluster C |
| GSM404197 | cluster C |
| GSM404198 | cluster C |
| GSM404207 | cluster C |
| GSM404208 | cluster C |
| GSM404209 | cluster C |
| GSM404210 | cluster C |
| GSM404211 | cluster C |
| GSM404212 | cluster C |
| GSM404224 | cluster C |
| GSM404230 | cluster C |
| GSM404231 | cluster C |
| GSM404234 | cluster C |
| GSM404237 | cluster C |
| GSM404238 | cluster C |
| GSM404246 | cluster C |
| GSM404249 | cluster C |
| GSM404250 | cluster C |
| GSM404255 | cluster C |
| GSM404257 | cluster C |
| GSM404268 | cluster C |
| GSM404269 | cluster C |
| GSM404271 | cluster C |
| GSM404276 | cluster C |
| GSM404277 | cluster C |
| GSM404280 | cluster C |

| | |
|---|---|
| GSM404281 | cluster C |
| GSM404282 | cluster C |
| GSM404283 | cluster C |
| GSM404285 | cluster C |
| GSM404287 | cluster C |
| GSM404288 | cluster C |
| GSM404289 | cluster C |
| GSM404290 | cluster C |
| GSM404291 | cluster C |
| GSM404292 | cluster C |
| GSM404293 | cluster C |
| GSM404295 | cluster C |
| GSM404296 | cluster C |
| GSM404298 | cluster C |
| GSM404303 | cluster C |
| GSM404305 | cluster C |
| GSM404306 | cluster C |
| GSM404308 | cluster C |
| GSM404312 | cluster C |

**Table S6. PRG pattern-related DEGs**

SLAMF6
CCR5
LRRC25
DENND1C

SH2D2A
CYP26A1
KIF21B
AF289551
LY86
PACSIN3
CCDC163P
SENCR
KCNMB3
CD33
GAS2L1
IFNL2
ERBB2
CAMSAP3
LOC101927752
DOK4
TMEM151A
CD3E
GATSL3
DEGS2
EPB41L1
CTBS
CARNS1
CD8A
CTC-459F4.3
GALR3

OPLAH
ELMO3
KATNA1
ABR
SNORA68
TRAV12-2
C3orf20
LOC101928443
PLA2G2D
LAIR2
B4GALT7
RNF11
CCDC113
MSC
GPR1
Igk
ATP8B1
DNAJC4
ZNF512B
HLX
DHRS11
LMNB2
ADK
ACAP3
MPST
IDO1

SEPT1
UNC93B1
HRAS
SLC39A7
GRAMD1A
SPATA18
ZBTB32
MRPS9
TRG-AS1
LOC284023
IGSF8
GTF2H3
SPI1
SLA
CYP27C1
LINC00865
PLCD4
ATF5
SSSCA1
COL9A3
NCKAP1L
RP11-111M22.3
LINGO1
PRKCD
BIN2
COBL

RASSF2
PGLYRP2
ST6GALNAC4
KLK11
TMEM255B
ELL
PSD
PMM2
VPS4B
NLRP7
SNPH
CDK2AP2
HOPX
MID2
MGC10814
ORMDL3
C9orf172
VSIG10L
LOC100289098
P2RX5
TOX2
NEDD9
PM20D2
CDH5
MEIS1
ACAP1

ODF3B
LY9
VIT
PPP1CB
TPRXL
PPP1R16B
TAGAP
PLAGL1
KIAA0513
HPCAL1
VNN2
BST1
ZCCHC5
CMTR2
NFE2L2
ENO2
C2orf88
BCL2L1
FOXN1
DCPS
FMNL1
ARRDC3
NSG1
CCL18
SRGN
CCR10

MANSC1
OLFML2B
LINC00582
LRMP
JSRP1
WWP1
AHCYL2
SASH3
FCRL5
ID2
ME1
NDUFAF3
SAMSN1
TNFRSF19
TP53AIP1
GALNT12
IL24
U2AF1L4
CD19
PTPN13
NFE2
LINC00467
TMEM40
KMO
SCNN1B
STX11

CASP6
LOC344887
EFCAB4A
FCGR3B
UNG
ARHGAP30
BSPRY
IL1B
HLA-DMB
SLC51A
CYTIP
GMPPA
PRPS1
MPV17
IFNAR2
LA16c-381G6.1
PIK3AP1
GLRX
MYO1F
IL4I1
DNAJB9
PRDM1
GSTA4
FAM107B
CYP11B2
P2RY6

RAC2
SLC7A7
HLF
KAT2B
HSD11B1
FAM84A
APOL6
BET1L
NME4
ERV9-1
LILRB1
MOGS
PLVAP
GPSM3
SHC1
MAF
GRAMD2
SDF4
SLC7A5
PAG1
TCIRG1
ASNS
HERPUD1
CRYBG3
ERP29
NMU

SPCS3
EFS
P2RY13
SRM
CCM2
EDEM1
GMFG
GPX7
WNT2
OSBPL6
FUOM
MEI1
CCR1
C4orf48
HSPA13
FPR1
LIN7C
MIAT
RRAGD
CXCR4
KRT15
LIME1
KLF8
DLX3
PROCR
RAB30

CXCL14
CHID1
SLC38A5
SERINC5
LOC100505664
SUSD4
FERMT3
TNFRSF1B
KLHL6
VAV3
ANKRD29
BMF
LANCL3
IRX4
IL2RG
GBP6
SIL1
CILP
TMEM176B
FUT8
UBE2J1
TRAPPC6A
PCDH17
GPR65
PTPN6
MEF2C

STXBP3
MNDA
CSF3R
SEL1L3
ANKRD36BP2
PIK3CG
STAP1
LY96
PAX9
CD180
SDF2L1
LOC80154
LOC101929272
ADAM28

**Table S7. GO enrichment results based on 278 PRG pattern-related DEGs**

| ONTOLOC | ID | Description | GeneRatic | BgRatio | pvalue | p.adjust | qvalue | geneID |
|---|---|---|---|---|---|---|---|---|
| BP | GO:0070661 | leukocyte proliferation | 21/227 | 318/18723 | 3.37E-10 | 4.64E-07 | 4.04E-07 | ERBB2/CD |
| BP | GO:0046651 | lymphocyte proliferation | 20/227 | 288/18723 | 3.75E-10 | 4.64E-07 | 4.04E-07 | ERBB2/CD |
| BP | GO:0032943 | mononuclear cell proliferation | 20/227 | 291/18723 | 4.50E-10 | 4.64E-07 | 4.04E-07 | ERBB2/CD |
| BP | GO:1903131 | mononuclear cell differentiation | 22/227 | 426/18723 | 1.22E-08 | 8.18E-06 | 7.12E-06 | SLAMF6/E |
| BP | GO:0002460 | adaptive immune response based on somatic rec | 20/227 | 356/18723 | 1.44E-08 | 8.18E-06 | 7.12E-06 | SLAMF6/C |
| BP | GO:0050670 | regulation of lymphocyte proliferation | 16/227 | 225/18723 | 1.64E-08 | 8.18E-06 | 7.12E-06 | ERBB2/CD |
| BP | GO:0032944 | regulation of mononuclear cell proliferation | 16/227 | 227/18723 | 1.85E-08 | 8.18E-06 | 7.12E-06 | ERBB2/CD |

| Table S8. KEGG enrichment results based on 278 PRG pattern-related DEGs | | | | | | | | |
|---|---|---|---|---|---|---|---|---|
| ID | Description | GeneRatio | BgRatio | pvalue | p.adjust | qvalue | geneID | Count |
| hsa04061 | action with cytokine and | 9/127 | 100/8159 | 2.33E-05 | 0.005204513 | 0.004869719 | /CCR10/IL24/CCR1/CXCR4/CXCL14/TNF | 9 |
| hsa04062 | emokine signaling pathw | 12/127 | 192/8159 | 4.13E-05 | 0.005204513 | 0.004869719 | CL18/CCR10/RAC2/SHC1/CCR1/CXCR4/ | 12 |
| hsa04060 | ne-cytokine receptor inte | 14/127 | 295/8159 | 0.0001874 | 0.014151452 | 0.013241125 | RSF19/IL24/IL1B/IFNAR2/CCR1/CXCR4/C | 14 |
| hsa04662 | ll receptor signaling path | 7/127 | 82/8159 | 0.0002708 | 0.014151452 | 0.013241125 | S/CD19/PIK3AP1/RAC2/LILRB1/VAV3/PT | 7 |
| hsa05340 | rimary immunodeficienc | 5/127 | 38/8159 | 0.0002808 | 0.014151452 | 0.013241125 | CD3E/CD8A/CD19/UNG/IL2RG | 5 |
| hsa03250 | Viral life cycle - HIV-1 | 6/127 | 63/8159 | 0.0004155 | 0.017450354 | 0.016327817 | CCR5/ELL/VPS4B/KAT2B/CXCR4/SERINC | 6 |
| hsa04640 | ematopoietic cell lineag | 7/127 | 99/8159 | 0.0008527 | 0.030697867 | 0.028723151 | 3/CD3E/CD8A/CD19/IL1B/HLA-DMB/C | 7 |

**Table S9. The 3 distinct gene patterns based on 278 PRG pattern-related DEGs.**

| ID | gene subtype |
|---|---|
| GSM261086 | gene cluster A |
| GSM261087 | gene cluster B |
| GSM261089 | gene cluster B |
| GSM261090 | gene cluster C |
| GSM261092 | gene cluster A |
| GSM261093 | gene cluster B |
| GSM261094 | gene cluster B |
| GSM261095 | gene cluster B |
| GSM261097 | gene cluster C |
| GSM261098 | gene cluster C |
| GSM261100 | gene cluster C |
| GSM261101 | gene cluster A |
| GSM261103 | gene cluster A |

| | |
|---|---|
| GSM261104 | gene cluster A |
| GSM261105 | gene cluster A |
| GSM261106 | gene cluster A |
| GSM261107 | gene cluster A |
| GSM261108 | gene cluster A |
| GSM261110 | gene cluster A |
| GSM261111 | gene cluster A |
| GSM261112 | gene cluster A |
| GSM261114 | gene cluster C |
| GSM261115 | gene cluster C |
| GSM261118 | gene cluster A |
| GSM261119 | gene cluster A |
| GSM261120 | gene cluster C |
| GSM261121 | gene cluster A |
| GSM261123 | gene cluster B |
| GSM261124 | gene cluster B |
| GSM261125 | gene cluster C |
| GSM261126 | gene cluster C |
| GSM261128 | gene cluster C |
| GSM261129 | gene cluster B |
| GSM261130 | gene cluster A |
| GSM261131 | gene cluster A |
| GSM261132 | gene cluster A |
| GSM261133 | gene cluster A |
| GSM261135 | gene cluster B |
| GSM261136 | gene cluster C |

| | |
|---|---|
| GSM261139 | gene cluster A |
| GSM261140 | gene cluster A |
| GSM261141 | gene cluster A |
| GSM261142 | gene cluster A |
| GSM261144 | gene cluster B |
| GSM261145 | gene cluster C |
| GSM261147 | gene cluster B |
| GSM261148 | gene cluster A |
| GSM261149 | gene cluster A |
| GSM261150 | gene cluster A |
| GSM261152 | gene cluster B |
| GSM261153 | gene cluster A |
| GSM261155 | gene cluster C |
| GSM261156 | gene cluster C |
| GSM261157 | gene cluster A |
| GSM261158 | gene cluster A |
| GSM261160 | gene cluster A |
| GSM261161 | gene cluster A |
| GSM261163 | gene cluster A |
| GSM261164 | gene cluster A |
| GSM261166 | gene cluster A |
| GSM261167 | gene cluster C |
| GSM261169 | gene cluster A |
| GSM261170 | gene cluster A |
| GSM261172 | gene cluster C |
| GSM261173 | gene cluster C |

| | |
|---|---|
| GSM261175 | gene cluster C |
| GSM261176 | gene cluster C |
| GSM261178 | gene cluster B |
| GSM261179 | gene cluster B |
| GSM261180 | gene cluster C |
| GSM261181 | gene cluster A |
| GSM261182 | gene cluster B |
| GSM261183 | gene cluster C |
| GSM261185 | gene cluster B |
| GSM261186 | gene cluster C |
| GSM261187 | gene cluster A |
| GSM261189 | gene cluster C |
| GSM261190 | gene cluster B |
| GSM261191 | gene cluster B |
| GSM261192 | gene cluster B |
| GSM261194 | gene cluster C |
| GSM261195 | gene cluster C |
| GSM261197 | gene cluster C |
| GSM261198 | gene cluster C |
| GSM261200 | gene cluster C |
| GSM261201 | gene cluster C |
| GSM261203 | gene cluster C |
| GSM261204 | gene cluster C |
| GSM261207 | gene cluster A |
| GSM261208 | gene cluster C |
| GSM261209 | gene cluster A |

| | |
|---|---|
| GSM261210 | gene cluster C |
| GSM261211 | gene cluster A |
| GSM261212 | gene cluster A |
| GSM261213 | gene cluster B |
| GSM261214 | gene cluster A |
| GSM261215 | gene cluster B |
| GSM261216 | gene cluster B |
| GSM261218 | gene cluster C |
| GSM261219 | gene cluster C |
| GSM261221 | gene cluster B |
| GSM261222 | gene cluster B |
| GSM261224 | gene cluster B |
| GSM261225 | gene cluster B |
| GSM261227 | gene cluster B |
| GSM261228 | gene cluster B |
| GSM261230 | gene cluster B |
| GSM261231 | gene cluster B |
| GSM261232 | gene cluster B |
| GSM261233 | gene cluster B |
| GSM261235 | gene cluster B |
| GSM261236 | gene cluster B |
| GSM261238 | gene cluster C |
| GSM261239 | gene cluster B |
| GSM261241 | gene cluster B |
| GSM261242 | gene cluster B |
| GSM261244 | gene cluster B |

| | |
|---|---|
| GSM261245 | gene cluster B |
| GSM261247 | gene cluster C |
| GSM261248 | gene cluster C |
| GSM261250 | gene cluster C |
| GSM261251 | gene cluster B |
| GSM261252 | gene cluster C |
| GSM261253 | gene cluster C |
| GSM261255 | gene cluster B |
| GSM261256 | gene cluster A |
| GSM261258 | gene cluster A |
| GSM261259 | gene cluster C |
| GSM261260 | gene cluster C |
| GSM261261 | gene cluster C |
| GSM261262 | gene cluster C |
| GSM261263 | gene cluster C |
| GSM261264 | gene cluster C |
| GSM261265 | gene cluster C |
| GSM261267 | gene cluster C |
| GSM261268 | gene cluster B |
| GSM261269 | gene cluster B |
| GSM261270 | gene cluster B |
| GSM261271 | gene cluster B |
| GSM261273 | gene cluster B |
| GSM261274 | gene cluster B |
| GSM261275 | gene cluster B |
| GSM261276 | gene cluster C |

| | |
|---|---|
| GSM261278 | gene cluster A |
| GSM261279 | gene cluster A |
| GSM261281 | gene cluster B |
| GSM261282 | gene cluster B |
| GSM261284 | gene cluster C |
| GSM261285 | gene cluster B |
| GSM261287 | gene cluster B |
| GSM261288 | gene cluster B |
| GSM261289 | gene cluster B |
| GSM261290 | gene cluster B |
| GSM261291 | gene cluster B |
| GSM261292 | gene cluster B |
| GSM261294 | gene cluster C |
| GSM261295 | gene cluster B |
| GSM261297 | gene cluster C |
| GSM261298 | gene cluster B |
| GSM261299 | gene cluster B |
| GSM261300 | gene cluster B |
| GSM261301 | gene cluster C |
| GSM261302 | gene cluster C |
| GSM261304 | gene cluster C |
| GSM261305 | gene cluster B |
| GSM261307 | gene cluster C |
| GSM261308 | gene cluster C |
| GSM261310 | gene cluster B |
| GSM261311 | gene cluster B |

| | |
|---|---|
| GSM261312 | gene cluster A |
| GSM261313 | gene cluster A |
| GSM261315 | gene cluster B |
| GSM261316 | gene cluster C |
| GSM261318 | gene cluster B |
| GSM261319 | gene cluster C |
| GSM261321 | gene cluster B |
| GSM261322 | gene cluster B |
| GSM261324 | gene cluster C |
| GSM261325 | gene cluster C |
| GSM261327 | gene cluster C |
| GSM261328 | gene cluster C |
| GSM261330 | gene cluster B |
| GSM261331 | gene cluster C |
| GSM404005 | gene cluster A |
| GSM404006 | gene cluster B |
| GSM404008 | gene cluster C |
| GSM404009 | gene cluster C |
| GSM404011 | gene cluster A |
| GSM404012 | gene cluster C |
| GSM404014 | gene cluster C |
| GSM404015 | gene cluster C |
| GSM404018 | gene cluster A |
| GSM404019 | gene cluster C |
| GSM404020 | gene cluster A |
| GSM404021 | gene cluster C |

| | |
|---|---|
| GSM404022 | gene cluster C |
| GSM404023 | gene cluster C |
| GSM404024 | gene cluster A |
| GSM404026 | gene cluster A |
| GSM404027 | gene cluster A |
| GSM404029 | gene cluster C |
| GSM404030 | gene cluster C |
| GSM404032 | gene cluster A |
| GSM404033 | gene cluster A |
| GSM404034 | gene cluster B |
| GSM404035 | gene cluster B |
| GSM404037 | gene cluster C |
| GSM404038 | gene cluster C |
| GSM404040 | gene cluster C |
| GSM404041 | gene cluster A |
| GSM404043 | gene cluster A |
| GSM404044 | gene cluster A |
| GSM404045 | gene cluster A |
| GSM404046 | gene cluster A |
| GSM404047 | gene cluster A |
| GSM404048 | gene cluster A |
| GSM404050 | gene cluster A |
| GSM404051 | gene cluster A |
| GSM404052 | gene cluster A |
| GSM404055 | gene cluster A |
| GSM404056 | gene cluster A |

| | |
|---|---|
| GSM404057 | gene cluster C |
| GSM404058 | gene cluster A |
| GSM404060 | gene cluster B |
| GSM404061 | gene cluster B |
| GSM404062 | gene cluster C |
| GSM404063 | gene cluster C |
| GSM404065 | gene cluster C |
| GSM404066 | gene cluster C |
| GSM404067 | gene cluster A |
| GSM404068 | gene cluster A |
| GSM404069 | gene cluster A |
| GSM404070 | gene cluster A |
| GSM404072 | gene cluster A |
| GSM404073 | gene cluster C |
| GSM404076 | gene cluster A |
| GSM404077 | gene cluster A |
| GSM404078 | gene cluster A |
| GSM404079 | gene cluster A |
| GSM404081 | gene cluster A |
| GSM404082 | gene cluster A |
| GSM404083 | gene cluster A |
| GSM404084 | gene cluster A |
| GSM404086 | gene cluster B |
| GSM404087 | gene cluster A |
| GSM404089 | gene cluster C |
| GSM404090 | gene cluster C |

| | |
|---|---|
| GSM404091 | gene cluster A |
| GSM404092 | gene cluster A |
| GSM404094 | gene cluster A |
| GSM404095 | gene cluster A |
| GSM404097 | gene cluster A |
| GSM404098 | gene cluster A |
| GSM404100 | gene cluster A |
| GSM404101 | gene cluster C |
| GSM404103 | gene cluster A |
| GSM404104 | gene cluster A |
| GSM404106 | gene cluster C |
| GSM404107 | gene cluster C |
| GSM404109 | gene cluster B |
| GSM404110 | gene cluster B |
| GSM404111 | gene cluster C |
| GSM404112 | gene cluster A |
| GSM404113 | gene cluster B |
| GSM404114 | gene cluster C |
| GSM404116 | gene cluster A |
| GSM404118 | gene cluster C |
| GSM404119 | gene cluster B |
| GSM404120 | gene cluster B |
| GSM404121 | gene cluster B |
| GSM404123 | gene cluster C |
| GSM404124 | gene cluster C |
| GSM404126 | gene cluster C |

| | |
|---|---|
| GSM404127 | gene cluster C |
| GSM404129 | gene cluster C |
| GSM404130 | gene cluster C |
| GSM404132 | gene cluster C |
| GSM404133 | gene cluster C |
| GSM404135 | gene cluster A |
| GSM404136 | gene cluster C |
| GSM404137 | gene cluster A |
| GSM404138 | gene cluster A |
| GSM404139 | gene cluster B |
| GSM404140 | gene cluster A |
| GSM404141 | gene cluster B |
| GSM404142 | gene cluster A |
| GSM404144 | gene cluster C |
| GSM404145 | gene cluster C |
| GSM404147 | gene cluster B |
| GSM404148 | gene cluster C |
| GSM404150 | gene cluster B |
| GSM404151 | gene cluster B |
| GSM404153 | gene cluster B |
| GSM404154 | gene cluster B |
| GSM404156 | gene cluster A |
| GSM404157 | gene cluster B |
| GSM404158 | gene cluster B |
| GSM404159 | gene cluster B |
| GSM404161 | gene cluster B |

| | |
|---|---|
| GSM404162 | gene cluster B |
| GSM404164 | gene cluster C |
| GSM404165 | gene cluster C |
| GSM404167 | gene cluster B |
| GSM404168 | gene cluster B |
| GSM404170 | gene cluster B |
| GSM404171 | gene cluster B |
| GSM404173 | gene cluster C |
| GSM404174 | gene cluster C |
| GSM404176 | gene cluster C |
| GSM404177 | gene cluster B |
| GSM404178 | gene cluster C |
| GSM404179 | gene cluster C |
| GSM404181 | gene cluster B |
| GSM404182 | gene cluster A |
| GSM404184 | gene cluster C |
| GSM404185 | gene cluster C |
| GSM404186 | gene cluster C |
| GSM404187 | gene cluster C |
| GSM404188 | gene cluster C |
| GSM404189 | gene cluster C |
| GSM404191 | gene cluster C |
| GSM404192 | gene cluster C |
| GSM404193 | gene cluster B |
| GSM404194 | gene cluster B |
| GSM404196 | gene cluster B |

| | |
|---|---|
| GSM404197 | gene cluster B |
| GSM404198 | gene cluster B |
| GSM404199 | gene cluster C |
| GSM404201 | gene cluster A |
| GSM404202 | gene cluster A |
| GSM404204 | gene cluster B |
| GSM404205 | gene cluster B |
| GSM404207 | gene cluster B |
| GSM404208 | gene cluster B |
| GSM404209 | gene cluster B |
| GSM404210 | gene cluster B |
| GSM404211 | gene cluster B |
| GSM404212 | gene cluster B |
| GSM404213 | gene cluster C |
| GSM404214 | gene cluster B |
| GSM404216 | gene cluster C |
| GSM404217 | gene cluster C |
| GSM404218 | gene cluster B |
| GSM404219 | gene cluster B |
| GSM404220 | gene cluster C |
| GSM404221 | gene cluster C |
| GSM404223 | gene cluster C |
| GSM404224 | gene cluster B |
| GSM404226 | gene cluster C |
| GSM404227 | gene cluster C |
| GSM404229 | gene cluster B |

| | |
|---|---|
| GSM404230 | gene cluster B |
| GSM404231 | gene cluster B |
| GSM404232 | gene cluster C |
| GSM404234 | gene cluster B |
| GSM404235 | gene cluster C |
| GSM404237 | gene cluster B |
| GSM404238 | gene cluster B |
| GSM404240 | gene cluster C |
| GSM404241 | gene cluster C |
| GSM404243 | gene cluster C |
| GSM404244 | gene cluster C |
| GSM404246 | gene cluster B |
| GSM404247 | gene cluster C |
| GSM404249 | gene cluster B |
| GSM404250 | gene cluster B |
| GSM404252 | gene cluster C |
| GSM404253 | gene cluster C |
| GSM404254 | gene cluster C |
| GSM404255 | gene cluster B |
| GSM404256 | gene cluster C |
| GSM404257 | gene cluster B |
| GSM404258 | gene cluster B |
| GSM404259 | gene cluster B |
| GSM404261 | gene cluster C |
| GSM404262 | gene cluster C |
| GSM404263 | gene cluster B |

| | |
|---|---|
| GSM404264 | gene cluster B |
| GSM404265 | gene cluster C |
| GSM404266 | gene cluster C |
| GSM404268 | gene cluster B |
| GSM404269 | gene cluster B |
| GSM404271 | gene cluster B |
| GSM404272 | gene cluster B |
| GSM404274 | gene cluster B |
| GSM404275 | gene cluster C |
| GSM404276 | gene cluster B |
| GSM404277 | gene cluster B |
| GSM404278 | gene cluster C |
| GSM404279 | gene cluster C |
| GSM404280 | gene cluster B |
| GSM404281 | gene cluster B |
| GSM404282 | gene cluster B |
| GSM404283 | gene cluster B |
| GSM404285 | gene cluster B |
| GSM404286 | gene cluster B |
| GSM404287 | gene cluster B |
| GSM404288 | gene cluster B |
| GSM404289 | gene cluster B |
| GSM404290 | gene cluster B |
| GSM404291 | gene cluster B |
| GSM404292 | gene cluster B |
| GSM404293 | gene cluster B |

| | |
|---|---|
| GSM404294 | gene cluster C |
| GSM404295 | gene cluster B |
| GSM404296 | gene cluster B |
| GSM404297 | gene cluster B |
| GSM404298 | gene cluster B |
| GSM404299 | gene cluster C |
| GSM404300 | gene cluster B |
| GSM404301 | gene cluster B |
| GSM404302 | gene cluster C |
| GSM404303 | gene cluster C |
| GSM404304 | gene cluster C |
| GSM404305 | gene cluster C |
| GSM404306 | gene cluster C |
| GSM404307 | gene cluster C |
| GSM404308 | gene cluster B |
| GSM404309 | gene cluster C |
| GSM404310 | gene cluster B |
| GSM404311 | gene cluster C |
| GSM404312 | gene cluster B |
| GSM404313 | gene cluster C |
| GSM404314 | gene cluster C |
